# Endeavor toward Redox-Responsive Transition Metal Contrast Agents Based on the Cross-Bridge Cyclam Platform

**DOI:** 10.1021/acs.inorgchem.3c03486

**Published:** 2024-01-10

**Authors:** Rocío Uzal-Varela, Aurora Rodríguez-Rodríguez, Daniela Lalli, Laura Valencia, Marcelino Maneiro, Mauro Botta, Emilia Iglesias, David Esteban-Gómez, Goran Angelovski, Carlos Platas-Iglesias

**Affiliations:** † Centro Interdisciplinar de Química e Bioloxía (CICA) and Departamento de Química, Facultade de Ciencias, Universidade da Coruña, A Coruña 15071, Galicia, Spain; ‡ Dipartimento di Scienze e Innovazione Tecnologica, Magnetic Resonance Platform (PRISMA-UPO), Universitá del Piemonte Orientale, Viale T. Michel 11, Alessandria 15121, Italy; § Departamento de Química Inorgánica, Facultad de Ciencias, Universidade de Vigo, As Lagoas, Marcosende 36310, Pontevedra, Spain; ∥ Departamento de Química Inorgánica, Facultade de Ciencias, Campus Terra, Universidade de Santiago de Compostela, Lugo 27002, Galicia, Spain; ⊥ Laboratory of Molecular and Cellular Neuroimaging, International Center for Primate Brain Research (ICPBR), Center for Excellence in Brain Science and Intelligence Technology (CEBSIT), Chinese Academy of Sciences (CAS), Shanghai 201602, PR China

## Abstract

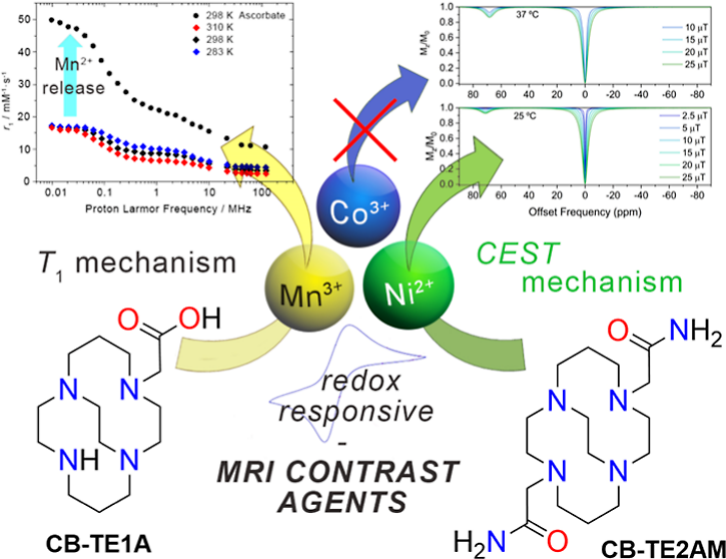

We present the synthesis and characterization of a series of Mn(III), Co(III), and Ni(II) complexes with cross-bridge cyclam derivatives (CB-cyclam = 1,4,8,11-tetraazabicyclo[6.6.2]hexadecane) containing acetamide or acetic acid pendant arms. The X-ray structures of [Ni(CB-TE2AM)]Cl_2_·2H_2_O and [Mn(CB-TE1AM)(OH)](PF_6_)_2_ evidence the octahedral coordination of the ligands around the Ni(II) and Mn(III) metal ions, with a terminal hydroxide ligand being coordinated to Mn(III). Cyclic voltammetry studies on solutions of the [Mn(CB-TE1AM)(OH)]^2+^ and [Mn(CB-TE1A)(OH)]^+^ complexes (0.15 M NaCl) show an intricate redox behavior with waves due to the Mn^III^/Mn^IV^ and Mn^II^/Mn^III^ pairs. The Co(III) and Ni(II) complexes with CB-TE2A and CB-TE2AM show quasi-reversible features due to the Co^III^/Co^II^ or Ni^II^/Ni^III^ pairs. The [Co(CB-TE2AM)]^3+^ complex is readily reduced by dithionite in aqueous solution, as evidenced by ^1^H NMR studies, but does not react with ascorbate. The [Mn(CB-TE1A)(OH)]^+^ complex is however reduced very quickly by ascorbate following a simple kinetic scheme (*k*_0_ = *k*_1_[AH^–^], where [AH^–^] is the ascorbate concentration and *k*_1_ = 628 ± 7 M^–1^ s^–1^). The reduction of the Mn(III) complex to Mn(II) by ascorbate provokes complex dissociation, as demonstrated by ^1^H nuclear magnetic relaxation dispersion studies. The [Ni(CB-TE2AM)]^2+^ complex shows significant chemical exchange saturation transfer effects upon saturation of the amide proton signals at 71 and 3 ppm with respect to the bulk water signal.

## Abstract 2

Cross-bridge derivatives of Mn(III), Co(III), and Ni(II) show potential as MRI contrast agents, taking advantage of both the classical T_1_ relaxation and chemical exchange saturation transfer (CEST) mechanisms.

## Introduction

Paramagnetic contrast agents have been used to enhance the image contrast in magnetic resonance imaging (MRI) since the late 1980s, when the first gadolinium-based contrast agent (GBCA), Magnevist, was approved for clinical use.^1−4^ Soon after, different GBCAs based on both macrocyclic and acyclic ligands entered clinical practice, with macrocyclic derivatives providing more inert complexes.^3^ After being injected into the bloodstream, GBCAs nonspecifically distribute into the extracellular space, where they magnetically interact with surrounding water molecules.^5^ These interactions induce relaxation enhancement of the water nuclei, which can be exploited to modulate their ^1^H NMR signal intensity and thus to improve the image contrast. In spite of the very successful use of GBCAs in clinical practice due to the valuable diagnostic and prognostic information they provide, some limitations have also been encountered. Indeed, traditional GBCAs allow us to visualize a single cell population in a specific anatomical region,^6,7^ and thus, they do not provide functional information on the area of interest. Therefore, huge efforts have been devoted to develop smart contrast agents, that is, compounds providing response to specific stimuli *in vivo*, such as changes in pH, concentration of cations and neurotransmitters, or enzymatic activity.^8−15^

Furthermore, despite being among the safest drugs in clinical use, some concerns have recently been raised about GBCAs due to some toxicity issues associated with the release of Gd(III) ions *in vivo* or their long-term deposition in the body.^16−18^ This triggered new research programs devoted to finding effective alternatives to the classical GBCAs based on transition metal ions involved in biological processes.^19−28^ Indeed, metal ions such as Mn(II) and Fe(III) are valuable *T*_1_ relaxation agents displaying efficiencies comparable to those of the traditional GBCAs.^29−32^ Transition metal ions like Fe(II), Co(II), and Ni(II) have also shown promise as paramagnetic chemical exchange saturation transfer (CEST) MRI contrast agents.^33−38^ These substances need pools of protons (generally –NH_2_ or –OH groups) in slow exchange, on the NMR time scale, with the bulk water.^39−41^ Application of a selective pulse at the resonance frequency of the exchangeable protons provokes saturation of the water resonance, resulting in decreased intensity of the bulk water signal. This allows switching the contrast on and off at will.^42^

**Chart 1:**
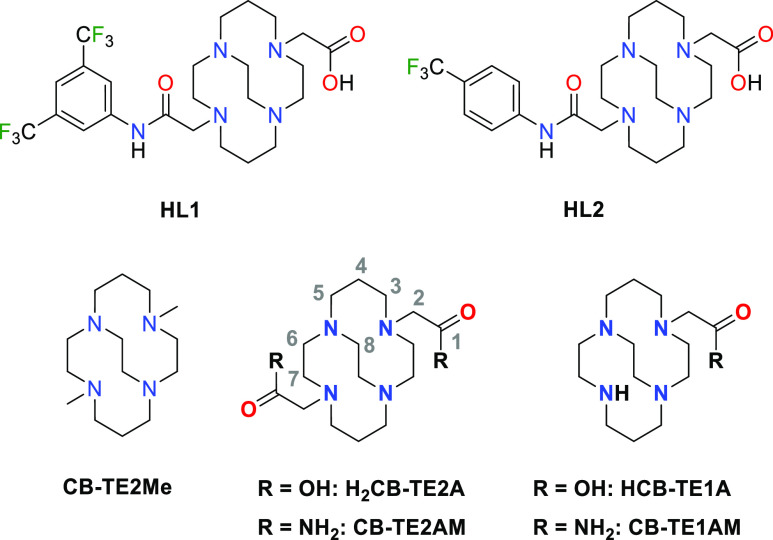
Ligands Discussed in This Work.

The macrocyclic cyclam platform (cyclam = 1,4,8,11-tetraazacyclotetradecane) has been widely used for stable complexation of transition metal ions with different purposes.^43−45^ A rigidified version of cyclam that contains an ethyl chain connecting two opposite N atoms of the macrocycle (CB-cyclam) has also found widespread use as a scaffold for transition metal complexation.^46−49^ In particular, this macrobicyclic platform improves the kinetic inertness of transition metal complexes for radiopharmaceutical applications. For instance, H_2_CB-TE2A and HCB-TE1A (Chart 1) and the related ligands form extremely inert complexes with Cu(II),^50−52^ which have been used to develop ^64^Cu-based probes for positron emission tomography.^53−56^ Furthermore, the cross-bridge cyclam platform stabilizes unusual oxidation states in some cases, like the Mn(IV) complex having two terminal hydroxo ligands [Mn(CB-TE2Me)(OH)_2_]^2+^.^57^ Cross-bridge derivatives also stabilize Mn(III) and Fe(III) over the corresponding divalent oxidation states.^58,59^ Closely related Mn and Fe cross-bridge derivatives were recently explored as oxidation catalysts, specifically for the bleaching of organic dyes, thanks to the stabilization of the +III and +IV oxidation states of the metal.^60^ The Ni(II) complexes of ligands H**L1** and H**L2** are also extremely inert and provide dual MRI response: ^1^H paraCEST signal due to the presence of exchangeable amide protons and ^19^F NMR signal.^61^

Most of the redox responsive paraCEST contrast agents reported in the literature are based on lanthanide metal ions like Eu(III) and Yb(III).^6,62^ In recent years, a few redox paraCEST agents based on transition metal centers [*i.e.*, Fe(II), Co(II), and Ni(II)] have been developed, as their response can be modulated by switching the II/III oxidation states.^6,33,62^ Furthermore, a limited number of *T*_1_ responsive agents based on the Mn(II)/Mn(III) pair were also reported.^63−65^ Developing MRI probes responsive to their redox environment necessitates a significant contribution from coordination chemistry.

In this work, we report a detailed study of the coordination chemistry of the CB-cyclam platform to assess its potential to develop redox-responsive MRI agents. We report the Mn(III) complexes with the pentadentate ligands HCB-TE1A and CB-TE1AM, which were designed to leave a vacant coordination position. We envisaged that reduction of these complexes to the Mn(II) analogues allows the coordination of a water molecule, thus providing *T*_1_ MRI response. Moreover, we prepared the Co(III) and Ni(II) complexes of the known amide ligand CB-TE2AM and explored their redox properties in aqueous solution with cyclic voltammetry experiments, as well as their paraCEST response. The X-ray structures of the [Ni(CB-TE2AM)]^2+^ and [Mn(CB-TE1AM)(OH)]^2+^ complexes are also reported. We note that cyclam cross-bridge derivatives for MRI applications have been described in the patent literature,^66,67^ but their coordination chemistry remains largely unexplored.

## Results and Discussion

### Synthesis

The cross-bridge cyclam derivative CB-TE2AM was prepared by alkylation of the parent cross-bridge cyclam using a variation of the synthesis described by Wong.^68^ We used diisopropylethylamine (DIPEA) rather than K_2_CO_3_ as a base, which allowed a straightforward isolation of the ligand that precipitated in the reaction medium. The diacetate analogue H_2_CB-TE2A, which was synthesized previously by alkylation of cross-bridge cyclam with ethylbromoacetate,^68^ was obtained here by hydrolysis of CB-TE2AM in aqueous 6 M HCl. The [Ni(CB-TE2AM)]Cl_2_ complex was synthesized in 85% yield by the reaction at 112 °C of the ligand and NiCl_2_ in 1-butanol in the presence of DIPEA as a base. The acetate derivative [Ni(CB-TE2A)] was obtained for comparative purposes. The use of DIPEA allows decreasing significantly the reaction time, presumably due to the proton sponge character of cross-bridge cyclam derivatives.^69,70^ The [Co(CB-TE2AM)]Cl_3_ complex was initially prepared using similar conditions, by reacting CoCl_2_·6H_2_O and the ligand in 1-butanol and in the presence of DIPEA, under an inert atmosphere. Subsequent oxidation of the Co(II) complex with oxygen in the presence of 1.5 equiv of aqueous HCl produced a blue solid that provided a single peak in the mass spectrum with *m*/*z* = 397.1755, corresponding to the [Co(C_16_H_30_N_6_O_2_)]^+^ entity. However, the ^1^H and ^13^C NMR spectra recorded in D_2_O evidenced the presence of two species in solution, one characterized by an effective *C*_2_ symmetry (eight ^13^C NMR signals) and the second characterized by a *C*_1_ symmetry. For the latter species, the ^13^C NMR spectrum displayed 13 of the 16 signals expected for a *C*_1_ symmetry (Figure S1, Supporting Information). We hypothesized that the asymmetric species could correspond to a complex in which one of the amide groups is deprotonated and likely coordinated to the metal ion through the N atom, as N-coordination of deprotonated amide groups to Co(III) was reported previously.^71,72^ We therefore carried out the synthesis using the same conditions in the absence of a base. This afforded a blue solid that corresponds to the [Co(CB-TE2AM)]Cl_3_ complex, as confirmed by the mass spectrum and ^1^H and ^13^C NMR studies (see below).

The monoalkylated cross-bridge cyclam derivative HCB-TE1A was prepared following the literature procedure, which involves the direct alkylation of cross-bridge cyclam with *t*-butyl bromoacetate in acetonitrile in the absence of a base.^73^ The use of a base (Na_2_CO_3_) afforded the given compound with a significantly lower yield.^74^ The proton sponge character of cross-bridge cyclam^70^ appears to be responsible for this effect as the presence of a proton in the macrobicyclic cavity likely hiders the alkylation of the second secondary amine N atom. A similar procedure afforded CB-TE1AM in good yield (89%).

The chloride salts of the Mn(III) complexes [Mn(CB-TE1AM)(OH)]Cl_2_ and [Mn(CB-TE1A)(OH)]Cl were obtained by the reaction of the ligand with Mn(NO_3_)_2_·4H_2_O in 1-butanol in the presence of DIPEA as a base, followed by oxidation of the Mn(II) complex with oxygen at room temperature.

### X-ray Structures

**Figure 1:**
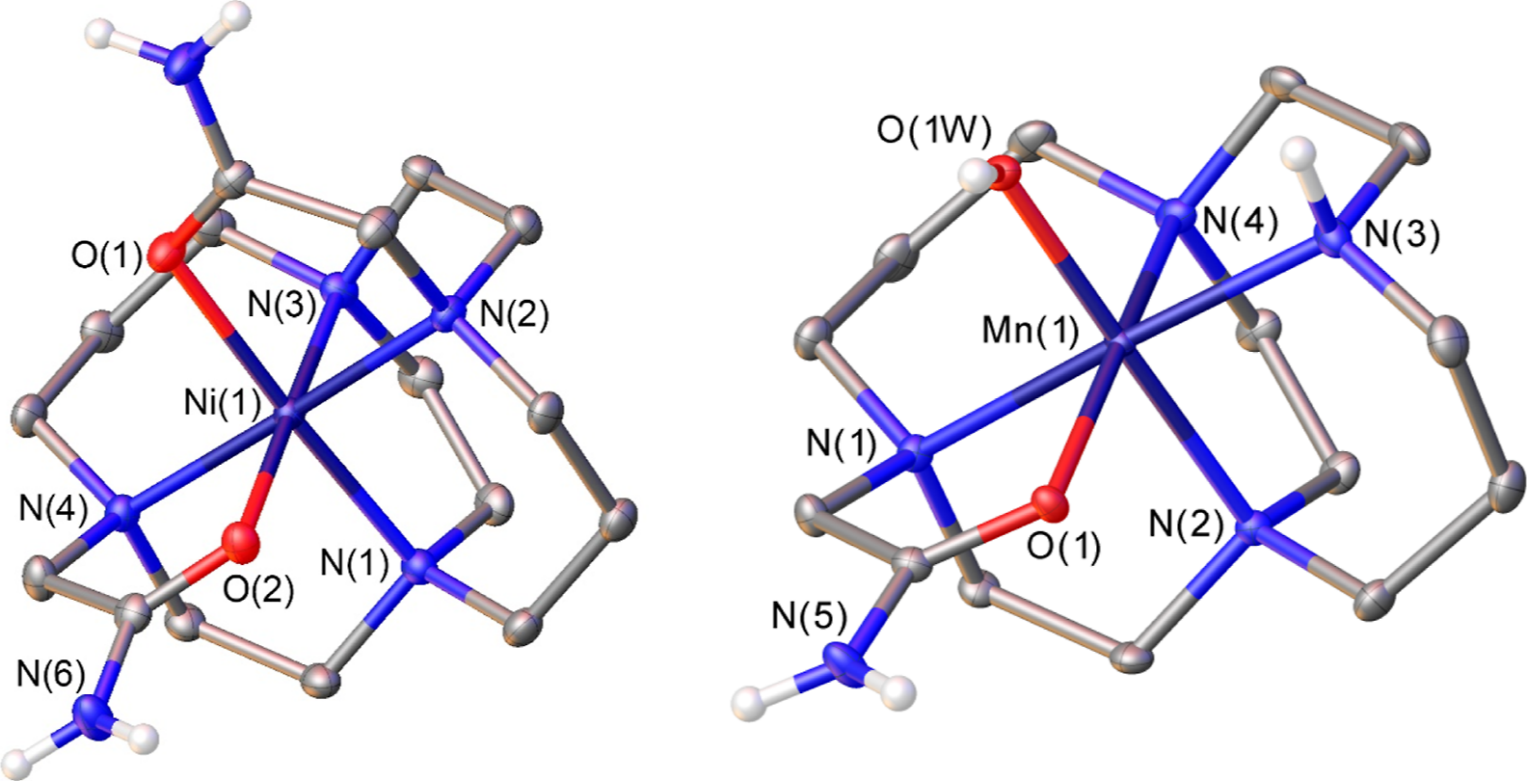
ORTEP^75^ view of the structure of the [Ni(CB-TE2AM)]^2+^ (left) and [Mn(CB-TE1AM)(OH)]^2+^ (right) complexes (50% ellipsoid probability). Hydrogen atoms and water molecules are omitted for simplicity.

The crystal structure of [Co(CB-TE2A)](PF_6_) was reported previously by Hubin *et al.*(76) The X-ray structure of [Ni(CB-TE2AM)]Cl_2_·2H_2_O contains the [Ni(CB-TE2AM)]^2+^ complex and water molecules involved in hydrogen bonding interactions with the amide NH_2_ groups and chloride anions. The Ni(II) ion in [Ni(CB-TE2AM)]^2+^ is directly coordinated to the four N atoms of the macrocyclic unit and the two oxygen atoms of the amide groups (Figure 1). The Ni–N distances fall in the range 2.06–2.08 Å (Table 1). These distances are similar to those observed previously for the related [NiL1]^+^ and [NiL2]^+^ complexes.^61^ However, longer Ni–N distances were observed for cross-bridge cyclam Ni(II) derivatives that either lack coordinating pendant arms or contain a single pendant arm coordinated to the metal ion (2.09–2.22 Å).^77,78^ Thus, the presence of two coordinating pendant arms appears to push the metal ion further into the interior of the macrobicyclic cleft. The Ni–O bonds are similar to those observed for six-coordinate Ni(II) complexes containing amide groups.^37,79,80^ The metal coordination environment is distorted octahedral, with *trans* angles >176° and *cis* angles in the range 83.7–95.2°.

**Table 1:** Bond Distances (Å) of the Metal Coordination Environments in [Ni(CB-TE2AM)]^2+^ and [Mn(CB-TE1AM)(OH)]^2+^ Obtained from X-ray Crystallographic Studies

Ni(1)–N(4)	2.0618(13)	Mn(1)–O(1W)	1.8397(16)
Ni(1)–O(1)	2.0618(11)	Mn(1)–N(3)	2.0762(19)
Ni(1)–N(2)	2.0662(13)	Mn(1)–N(2)	2.0804(18)
Ni(1)–N(1)	2.0675(13)	Mn(1)–O(1)	2.1366(15)
Ni(1)–N(3)	2.0801(13)	Mn(1)–N(1)	2.1375(18)
Ni(1)–O(2)	2.0832(11)	Mn(1)–N(4)	2.2262(18)

The Mn(III) complex with CB-TE1AM was crystallized as the hexafluorophosphate salt [Mn(CB-TE1AM)(OH)](PF_6_)_2_, which contains the [Mn(CB-TE1AM)(OH)]^2+^ complex (Figure 1). The metal ion is coordinated to the four amine N atoms of the ligand, the oxygen atom of the acetamide pendant, and a hydroxide anion. The structure of the complex resembles that of the acetate adduct [Mn(CB-TE2Me)(OH)(OAc)]^+^ reported by Hubin.^58^ The metal ion displays a distorted octahedral coordination characteristic of high-spin *d*^4^ complexes with Jahn–Teller distortion.^81^ The distance involving the hydroxide ligand is particularly short [Mn(1)–O(1W) = 1.8397(16) Å] compared with the distance from the metal ion to the oxygen atom of the acetamide group [Mn(1)–O(1) = 2.1366(15) Å] (Table 1). Similar Mn–O distances were found for Mn(III) complexes containing terminal OH ligands (typically 1.78–1.88 Å).^58,82−84^ The *trans* position with respect to O(1) is occupied by N(4), which provides a long Mn(1)–N(4) bond of 2.2262(18) Å, resulting in an axially elongated octahedral coordination with equatorial Mn(1)-N bond distances in the range ∼2.08–2.14 Å.

The macrocyclic units in [Ni(CB-TE2AM)]^2+^ and [Mn(CB-TE1AM)(OH)]^2+^ adopt the typical *cis*-V conformation observed for complexes of cross-bridge derivatives of small metal ions,^61,85−87^ with the bicyclo[6.6.2] fragments adopting [2323] conformations^88,89^ and the six-membered chelate rings showing chair conformations. This is in contrast with the Cu(II) and Zn(II) complexes of CB-TE2AM, in which the macrocycle adopts an unusual [2233]/[2233] bicyclic conformation.^90^ However, the bicyclo[6.6.2] fragments in the acetate derivative [Cu(CB-TE2A)] adopt rectangular [2323] conformations.^68^

### Absorption Spectra

The absorption spectra of the Ni(II) complexes display three weak absorption band envelopes (ε < ∼20 M^–1^ cm^–1^, Table 2) due to Ni(II) d–d transitions, as expected for octahedral complexes (Figure S2, Supporting Information).^91^ The lowest energy band is asymmetrical on the lower energy side, which can be ascribed to the spin-forbidden transition involving the excited ^1^E_g_ excited state.^92^ The maximum of the absorption band with the lowest energy provides estimates of the crystal field splitting energy of Δ_o_ = 12,470 and 12,390 cm^–1^ for [Ni(CB-TE2A)] and [Ni(CB-TE2AM)]^2+^, respectively. A Δ_o_ value of 10,215 cm^–1^ was reported for [Ni(CB-TE2Me)Cl_2_] in acetonitrile solution.^78^ This indicates that the acetate and acetamide groups provide similar ligand field strengths, which in turn are higher than those caused by chloride ligands. The absorption spectra of the Co(III) complexes recorded in acetonitrile solution were reported previously.^76^ The spectra recorded in water (Figure S3, Supporting Information) are very similar to those observed in acetonitrile. Two relatively strong absorption bands due to d–d transitions are observed with maxima at ∼495 and 355 nm, providing Δ_o_ values of 24,080 and 24,000 cm^–1^ for [Co(CB-TE2A)]^+^ and [Co(CB-TE2AM)]^3+^, respectively.

**Table 2:** Electronic Spectral Data Recorded from Aqueous Solutions of the Complexes with Cross-Bridge Derivatives

	λ_max_/nm	ε/M^–^^1^ cm^–^^1^
[Ni(CB-TE2A)]	802	4
	516	3
	390 (s)^a^	6
	326	13
[Ni(CB-TE2AM)]^2+^	807	17
	514	14
	403 (s)^a^	4
	329	9
[Co(CB-TE2A)]^+^	493	82
	356	134
[Co(CB-TE2AM)]^3+^	495	113
	358	156
[Mn(CB-TE1A)(OH)]^+^	511	131
	411	204
	377	211
[Mn(CB-TE1AM)(OH)]^2+^	500	31
	387	83

a Shoulder.

**Figure 2:**
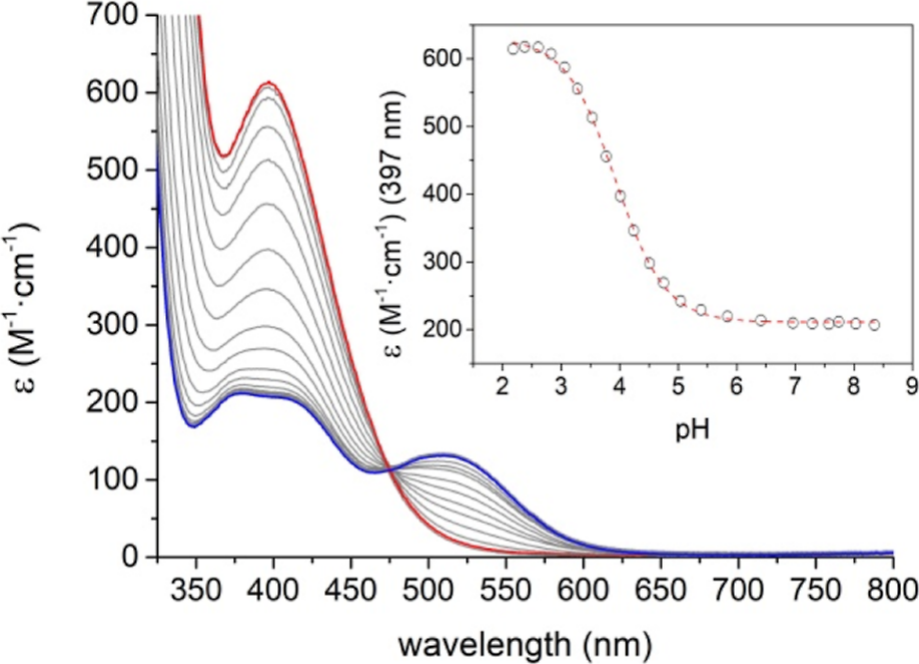
Absorption spectra of the [Mn(CB-TE1A)(OH)]^+^ complex recorded as a function of pH. The blue trace corresponds to pH 8.35 and the red trace corresponds to pH 2.18. The inset shows the variation of the molar extinction coefficient at 397 nm with pH.

The absorption spectra of [Mn(CB-TE1A)(OH)]^+^ recorded at pH values of ca. 6.0–8.3 present a maximum at 511 nm whose extinction coefficient is consistent with a d–d absorption transition. Two additional maxima are observed at 411 and 377 nm (Figure 2, see also Table 2). The absorption spectrum of [Mn(CB-TE2Me)(OH)(OAc)]^+^ shows similar features, with weak absorptions at 478 and 409 nm.^58^ The absorption spectrum experiences important changes below pH ∼ 6 with a well-defined isosbestic point at 475 nm. The intensity of the absorption maximum at 511 nm decreases on lowering the pH as a new more intense band with a maximum at 397 nm arises. These spectral changes are attributed to the protonation of the hydroxide ligand, which is characterized by a p*K*_a_ of 3.93(3). This value is somewhat lower than that determined for [Mn(CB-TE2Me)Cl_2_]^+^ (p*K*_a_ = 5.87).^58^ The absorption spectrum of the amide derivative [Mn(CB-TE1AM)(OH)]^2+^ recorded at pH 8.30 shows similar features, though the maximum in the visible region is observed at a higher energy (500 nm, Table 2). The spectrum changes below pH ∼ 6, though the lack of well-defined isosbestic points indicates the presence of more than one process taking place upon lowering pH (Figure S4, Supporting Information).

### NMR Studies

The ^1^H NMR spectra of the [Co(CB-TE2AM)]^3+^ and [Co(CB-TE2A)]^+^ complexes recorded in D_2_O solution (pH ∼ 7.0) are compatible with an effective *C*_2_ symmetry in solution. This is confirmed by the ^13^C NMR spectra, which show the eight signals expected for *C*_2_ symmetry. The ^1^H NMR spectra point to particularly rigid complexes. The methylenic protons of the pendant arms are characterized by AB spin systems with ^2^*J* values of ∼17 Hz. A full assignment of the ^1^H and ^13^C spectra was achieved with the aid of COSY, HSQC, and HMBC spectra (Tables S1 and S2 and Figures S5–S10).

**Figure 3:**
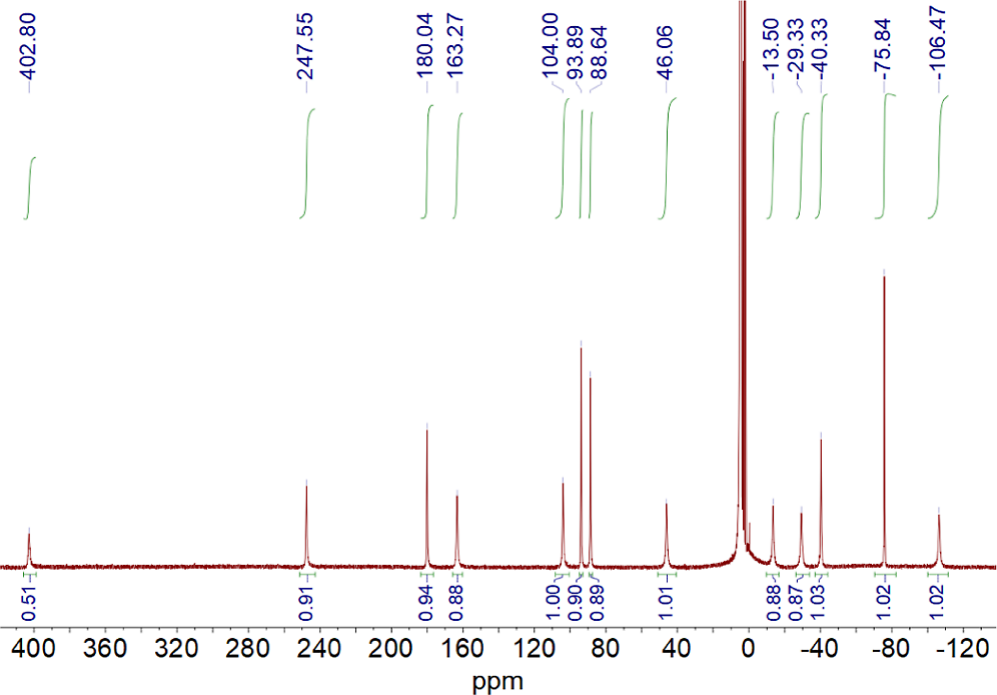
^1^H NMR spectrum (500 MHz) of the [Co(CB-TE2AM)]^2+^ complex recorded in D_2_O solution, pH 3.76. The Co(II) complex was generated by adding an ascorbate excess to a solution of the Co(III) complex in a glovebox, employing a screw-cap NMR tube. The presence of traces of oxygen results in very fast oxidation to the Co(III) complex. The sharp signals close to the residual HDO solvent peak are due to partial oxidation of the complex.

Addition of sodium dithionite to a solution of [Co(CB-TE2AM)]^3+^ in D_2_O provokes a perceptible color change in the solution from pink to pale pink. The ^1^H NMR spectrum (Figure 3) shows drastic changes, with the signals due to the diamagnetic Co(III) complex disappearing, while a set of paramagnetically shifted signals arises in the range ∼403 to −106 ppm. This spectrum is typical of paramagnetic Co(II) complexes. The reduced complex is very sensitive to oxidation in the presence of oxygen.

**Figure 4:**
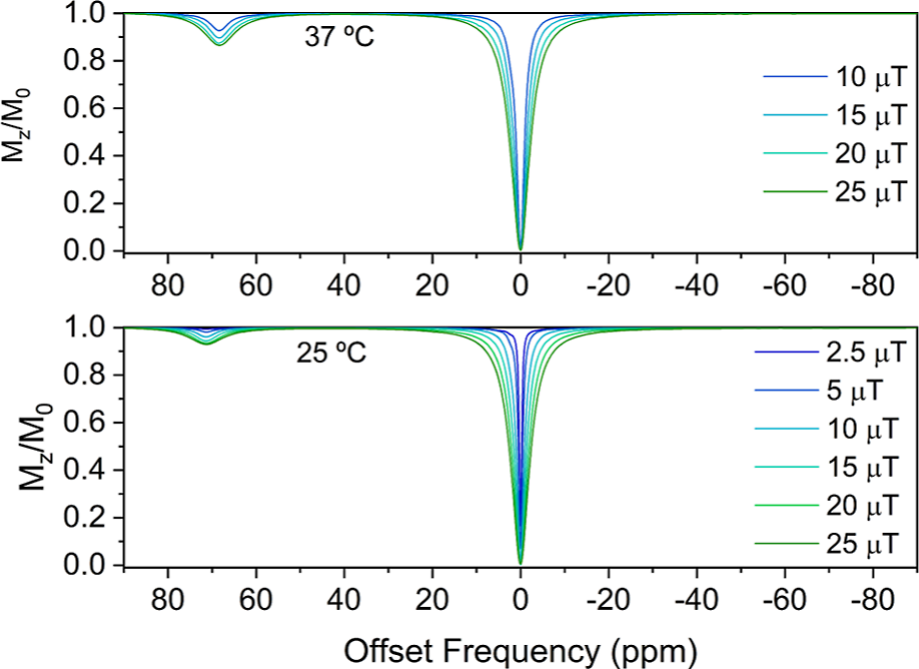
Z-spectra of the 7 mM [Ni(CB-TE2AM)]^2+^ complex acquired at 298 K (top) and 310 K (bottom) recorded using different saturation powers *B*_1_ (11.75 T, saturation time 2 s, pH 7.0).

The ^1^H NMR spectrum (500 MHz) of the [Ni(CB-TE2AM)]^2+^ complex recorded in D_2_O solution shows very broad signals between 160 and −30 ppm, with line widths in the range of 700–4000 Hz, indicating very short ^1^H *T*_2_ values (Figure S11). This is likely related to the octahedral coordination environment around the metal ion, which results in a slow electronic relaxation. Well-resolved ^1^H NMR spectra of Ni(II) complexes were reported for the severely distorted octahedral coordination.^37^ The eight resonances observable in the ^1^H NMR spectrum recorded in D_2_O are compatible with an expected *C*_2_ symmetry. The two additional signals resonating at 71 and 3 ppm (with respect to the bulk water signal) showing up in pure water are ascribable to protons in slow exchange with the bulk water (Figure S11, Supporting Information). A water solution of the [Ni(CB-TE2AM)]^2+^ complex (7 mM) displays weak CEST features at these δ values when applying a low saturation power of 2.5 μT (Figure 4), with the signal at 3 ppm being barely visible below the bulk water signal. These CEST effects are typical of Ni(II) complexes containing primary amide exchangeable protons.^36,37,80^ The signal at 71 ppm can be attributed to the amide protons in the *trans* position with respect to the carbonyl C=O bond, while the signal at 3 ppm corresponds to *cis* amide NH protons.^38^ The CEST effect at 71 ppm increases in intensity as *B*_1_ increases, reaching 7% at 25 μT. A further raise in intensity of the CEST effect was observed when heating to 37 °C, reaching 14% at 25 μT, but remains modest compared to other paraCEST agents.^36^

The CEST spectra acquired with different saturation pulses were analyzed using the Bloch–McConnell equations to derive the exchange rates of amide protons (Figures S12 and S13, Supporting Information).^42,93^ The fits of the data recorded at 25 °C to a 3-pool model afforded amide exchange rates of 1460 ± 130 and 1384 ± 151 Hz for the signals at 71 and 3 ppm, respectively. These values are one order of magnitude higher than those reported for Ni(II) complexes containing acetamide pendants (0.24–0.36 kHz).^36^ At 37 °C, the CEST feature at 3 ppm is not visible, and thus, the spectra were analyzed using a 2-pool model, which afforded an amide exchange rate of 2064 ± 348 Hz for the signal at 71 ppm.

The amide exchange rates determined from the analysis of the CEST spectra are not far from the ideal value (∼2700 Hz with *B*_1_ = 10 μT).^94^ Thus, the relatively weak CEST effects obtained here appear to be related to the fast relaxation of the bulk water signal induced by the paramagnetic Ni(II) ion. Indeed, the fits of the CEST data afford *T*_2_ values for the bulk water signal of 0.10 and 0.15 s at 25 and 37 °C, respectively, while typically, longer *T*_2_ values of ∼0.5 s are observed.^95^ The very broad signals observed in the ^1^H high-resolution NMR spectrum of the complex (Figure S11, Supporting Information) and the broad 71 ppm signal in the CEST spectrum (Figure 4) point to fast paramagnetic relaxation, supporting this analysis. Of note, paramagnetic relaxation in Ni(II) complexes increases sharply with the applied magnetic field,^96^ and thus, it is likely that the detrimental effect of relaxation diminishes at lower magnetic fields (*i.e.*, 7 T).

### Cyclic Voltammetry

Cyclic voltammetry measurements were carried out to analyze the redox properties of the [Co(CB-TE2AM)]^3+^ and [Ni(CB-TE2AM)]^2+^ complexes. The acetate derivatives [Co(CB-TE2A)]^+^ and [Ni(CB-TE2A)] were also investigated for comparative purposes. Electrochemical experiments were recorded using solutions of the complexes in 0.15 M NaCl employing a Ag/AgCl reference electrode. Electrochemical data are summarized in Table 3.

**Table 3:** Redox Potentials (*vs* Ag/AgCl) Obtained for Co(III), Ni(II) (0.15 M NaCl, pH 7.1, 0.01 V s^–1^), and Mn(III) (0.15 M NaCl, 0.05 V s^–1^) Complexes Using Cyclic Voltammetry

	*E*_c_/mV	*E*_a_/mV	*E*_1/2_/mV	*E*_a_–*E*_c_/mV	assignment
[Co(CB-TE2AM)]^3+^	–239	–151	–195	88	Co^III^/Co^II^
[Co(CB-TE2A)]^+^	–518	–388	–453	130	Co^III^/Co^II^
[Ni(CB-TE2AM)]^2+^	1030	1125	1078	95	Ni^II^/Ni^III^
[Ni(CB-TE2A)]	781	861	821	80	Ni^II^/Ni^III^
[Mn(CB-TE1AM)(OH)]^2+^^a^	45	424	235	379	Mn^II^/Mn^III^
	722	1022	872	300	Mn^III^/Mn^IV^
[Mn(CB-TE1A)(OH)]^+^^b^	–166	288	61	454	Mn^II^/Mn^III^
	612	749	680	137	Mn^III^/Mn^IV^
[Mn(CB-TE1A)(H_2_O)]^2+^^c^	156	573	365	417	Mn^II^/Mn^III^

a Recorded at pH 7.01.

b Recorded at pH 8.4.

c Recorded at pH 2.18.

**Figure 5:**
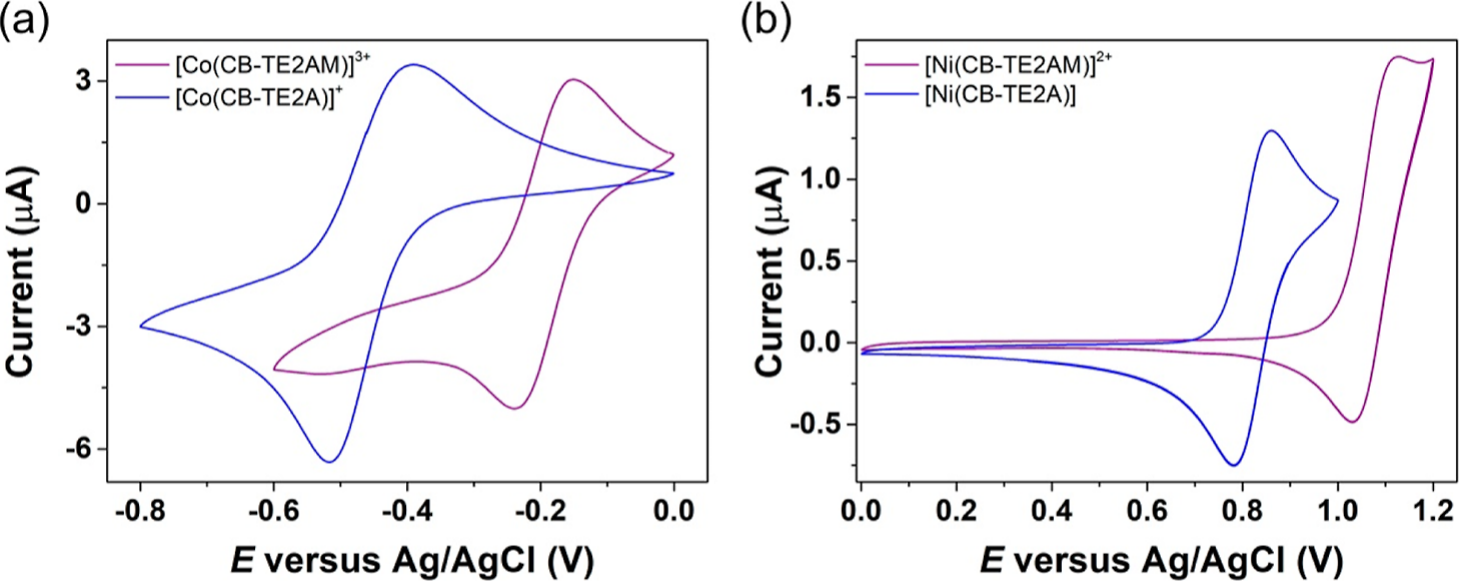
Cyclic voltammograms of the Co(III) (a) and Ni(II) (b) complexes recorded from ca. 2 mM aqueous solutions (0.15 M NaCl, pH 7.1, scan rate 0.01 V s^–1^).

The Co(II) complexes display cyclic voltammograms characteristic of the Co(III)/Co(II) redox system.^97^ The cyclic voltammogram recorded for [Co(CB-TE2AM)]^3+^ using a scan rate of 10 mV s^–1^ is characteristic of a quasi-reversible process with *E*_1/2_ = −195 mV (Δ*E*_p_ = 88 mV) (*vs* Ag/AgCl, Figure 5). The carboxylate analogue displays a more negative *E*_1/2_ value of −453 mV characterized by a larger separation of the anodic and cathodic waves (Δ*E*_p_ = 130 mV). The separation between the anodic and cathodic peaks increases upon increasing the scan rate, a situation typical of quasi-reversible systems. This is because the rate of mass transport becomes similar or quicker than the rate of electron transfer.^98^ The peak currents of the anodic and cathodic waves show linear dependence with the square root of the scan rate (Figure S14, Supporting Information), suggesting a diffusion controlled process.^99^ The more negative *E*_1/2_ value observed for [Co(CB-TE2A)]^+^ than for the amide analogue implies a more important stabilization of Co(III) by the hard carboxylate donor atoms than by the neutral amide groups.

The redox potentials of *E*_1/2_ = −195 and −453 mV measured *vs* Ag/AgCl for [Co(CB-TE2AM)]^3+^ and [Co(CB-TE2A)]^+^, respectively, correspond to *E*_1/2_ = +15 mV and *E*_1/2_ = −243 mV with respect to the NHE.^100^ These reduction potentials are clearly above the threshold for common bioreductants (−0.4 V *vs* NHE), which makes these complexes potential candidates for the design of redox bioresponsive agents. The reduction potential obtained for [Co(CB-TE2AM)]^3+^ compares well to that measured in acetonitrile solution (*E*_1/2_ = +13 mV). However, the solvent has an important influence in the case of [Co(CB-TE2A)]^+^, for which a half-potential value of *E*_1/2_ = −565 mV was reported in acetonitrile.^76^ Interestingly, cross-bridge derivatives containing noncoordinating pendant arms are characterized by *E*_1/2_ values measured in acetonitrile solution > + 240 mV.^101^ The presence of the two acetate or acetamide pendant arms likely pushes the metal ion to the interior of the macrocyclic cavity, thereby stabilizing the small Co(III) ion. This is in line with the Ni–N distances discussed above.

The cyclic voltammograms recorded for the Ni(II) complexes are characteristic of reversible systems involving the Ni(II)/Ni(III) pair,^78^ with the peak currents of the anodic and cathodic peaks displaying linear dependence with the square root of the scan rate (Figure S15, Supporting Information). As observed for the Co(III) analogues, the *E*_1/2_ values reflect a more important stabilization of Ni(III) by the hard acetate groups than for the amide derivative. The *E*_1/2_ values measured here in aqueous solution are comparable to those determined in acetonitrile for cross-bridge Ni(II) derivatives lacking coordinating pendant arms.^78,101^

**Figure 6:**
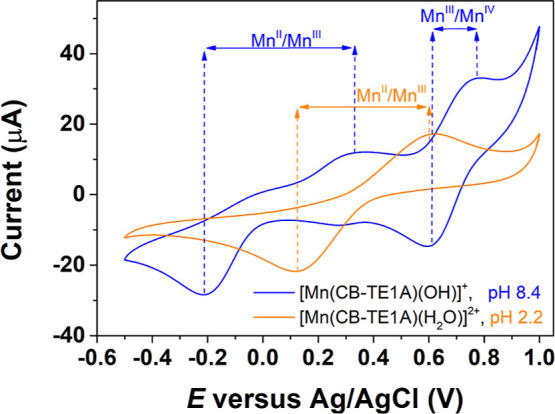
Cyclic voltammograms of the Mn(III) complex of CB-TE1A^–^ recorded at different pH values (0.15 M NaCl, scan rate 0.25 V s^–1^).

The Mn(III) complexes display a more complicated redox behavior. The cyclic voltammograms recorded close to neutral pH (Figure 6, see also Figure S16, Supporting Information), present waves with *E*_1/2_ values of 872 and 680 mV for [Mn(CB-TE1AM)(OH)]^2+^ and [Mn(CB-TE1A)(OH)]^+^, respectively, which are associated with the Mn^III^/Mn^IV^ couple. The *E*_1/2_ values indicate that the hard acetate group is more efficient in stabilizing Mn(IV) than the acetamide pendant, as would be expected. The Mn^III^/Mn^IV^ redox couple is irreversible for [Mn(CB-TE1AM)(OH)]^2+^, as demonstrated by the large separation of the anodic and cathodic waves, which further increases upon increasing the scan rate (Figure S16, Supporting Information). The acetate derivative [Mn(CB-TE1A)(OH)]^+^ displays a quasi-reversible Mn^III^/Mn^IV^ redox couple (Figure 6). The two complexes present Mn^III^/Mn^II^ features characterized by large *E*_a_ – *E*_c_ values, which indicates that the complexes experience large rearrangements of the metal coordination environments upon reduction to Mn(II). The cathodic and anodic currents are however similar, indicating a certain degree of reversibility.

The cyclic voltammogram of [Mn(CB-TE1A)(H_2_O)]^2+^ was recorded at pH 2.2, well below the p*K*_a_ of 3.93(3) determined by spectrophotometry (Figure 6). Under these conditions, the waves associated with the Mn^III^/Mn^IV^ couple are no longer observed, which evidence that the presence of a hydroxide ligand is responsible for Mn^IV^ stabilization. Likewise, the *E*_1/2_ value corresponding to the Mn^II^/Mn^III^ couple shifts to more positive potentials, which indicates a stabilization of Mn^II^ upon protonation of the hydroxide ligand. The Mn^II^/Mn^III^ redox couple is characterized by a large separation of the anodic and cathodic waves, which further increases on decreasing the scan rate, but similar currents of the anodic and cathodic waves.

### Reactivity in the Presence of Ascorbate

The reactivity of the [Mn(CB-TE1A)(OH)]^+^ and [Co(CB-TE2AM)]^3+^ complexes with ascorbate was analyzed using spectrophotometric studies. Ascorbate is a common bioreducing agent with important biological functions.^102^ The absorption spectrum of the [Co(CB-TE2AM)]^3+^ complex remains unchanged in the presence of excess ascorbate (Figure S17, Supporting Information). The lack of changes observed for the *d*–*d* absorption band at 493 nm indicates that the complex is not reduced by ascorbate. However, [Mn(CB-TE1A)(OH)]^+^ experiences a fast reaction in the presence of ascorbate, which takes place in the stopped-flow time scale. The reaction was followed spectrophotometrically by monitoring the disappearance of the absorption band of the complex at 320 nm (Figure S18, Supporting Information). The observed spectral changes are compatible with the following reaction
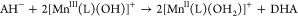
Here, AH^–^ and DHA represent ascorbate and its oxidation product dehydroascorbic acid,^103^ respectively, and L denotes the CB-TE1A^–^ ligand. The reaction is accompanied by the loss of the red color characteristic of the Mn(III) complex. At the pH values used for this study, ascorbic acid is present in solution as the ascorbate anion AH^–^ (p*K*_a_ = 4.04).^104^ The formation of DHA is likely the result of the well-established disproportionation of the monodehydroascorbate radical anion A^–^.^105^

**Figure 7:**
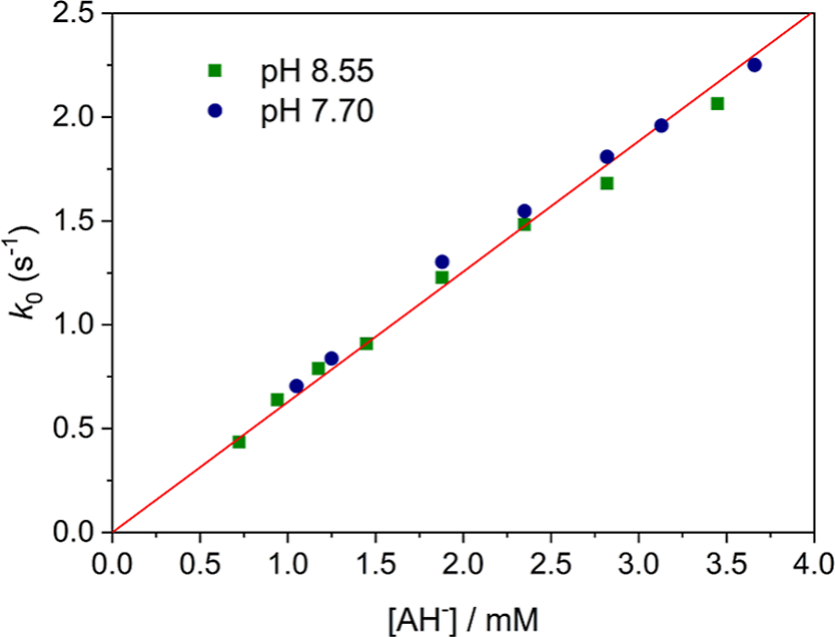
Pseudo-first-order rate constants for the reaction of [Mn(CB-TE1A)(OH)]^+^ with ascorbate ([Tris]_tot_ = 0.050 M; *I* = 0.15 M NaCl) at pH 8.55 (green squares) and pH 7.70 (blue circles). The red line corresponds to the linear fit of the data to *k*_0_ = *k*_1_[AH^–^] with *k*_1_ = 628 ± 7 M^–1^ s^–1^.

The reduction reaction was first investigated using 0.1 M phosphate buffer in the pH range 5.46 < pH < 7.86 (*I* = 0.12 M NaCl). A large excess of ascorbate (at least 18-fold) was used to ensure pseudo-first-order conditions. The pseudo-first-order rate constants *k*_0_ were found to vary significantly with pH. The values of *k*_0_ remain fairly constant below pH 6.5 and increase sharply above this pH (Figure S19, Supporting Information). Furthermore, the rate constant measured in phosphate buffer is 2 orders of magnitude lower than those obtained using Tris buffer (0.050 M, *I* = 0.15 M NaCl). Conversely, the rate constants do not vary with pH within experimental error in Tris buffer (Figure 7). These results indicate that phosphate interferes in the reaction in some way. The values of *k*_0_ show a linear dependence with the ascorbate concentration with a slope of *k*_1_ = 628 ± 7 M^–1^ s^–1^ and a negligible intercept. Thus, the reduction process follows a simple kinetic scheme according to *k*_0_ = *k*_1_[AH^–^].

**Figure 8:**
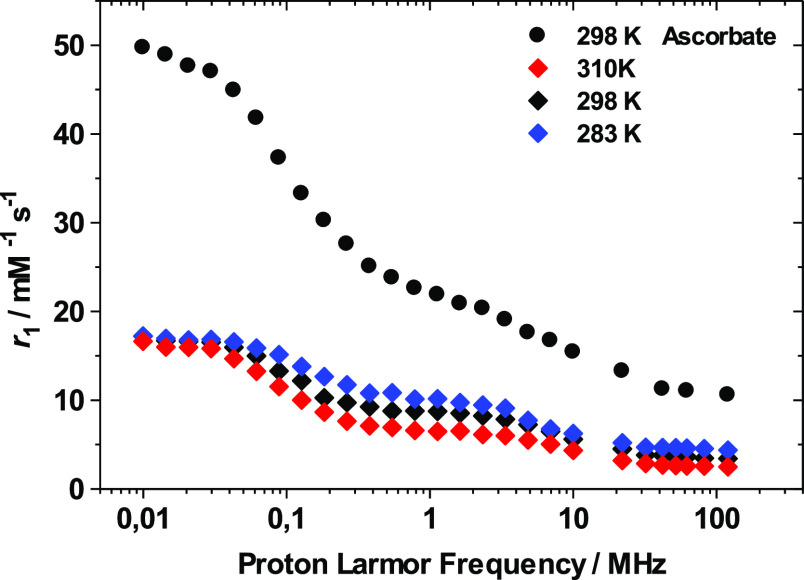
^1^H NMRD profiles recorded for the [Mn(CB-TE1A)(OH)]^+^ complex in the absence of ascorbic acid at different temperatures [283 K (blue ◆), 298 K (◆), and 310 K (red ◆) and in the presence of an excess (5 equiv) of ascorbate (298 K (●))].

The reduction of the [Mn(CB-TE1A)(OH)]^+^ complex by ascorbate was further examined using ^1^H nuclear magnetic relaxation dispersion (NMRD) measurements. In these experiments, the paramagnetic relaxation enhancement of the water proton nuclei is measured as a function of the ^1^H Larmor frequency, ranging between 0.01 and 120 MHz in the present case. In the presence of a paramagnetic solute, the relaxation enhancement induced by the paramagnetic species, normalized to a 1 mM concentration of the agent, is called relaxivity, *r*_1_. Surprisingly, the ^1^H NMRD profile recorded immediately after the dissolution of the complex in H_2_O (pH = 5.0) displays a first dispersion in the range 4–32 MHz together with a second dispersion at a low field (0.02–0.4 MHz). The latter is characteristic of a scalar contribution to ^1^H relaxivity, which is a feature of the aqueous complex [Mn(H_2_O)_6_]^2+^.^106,107^ This second dispersion was systematically observed in spite of the careful purification of the complex with high-performance liquid chromatography (HPLC). Furthermore, the X-band electron paramagnetic resonance (EPR) spectrum recorded at 298 K of the solution displays the typical six lines of [Mn(H_2_O)_6_]^2+^ spectra (Figure S20, Supporting Information), rather than a very broad X-band EPR signal typical of Mn(II) complexes with polyaminocarboxylate ligands.^108,109^ This suggests the presence of a thermodynamic equilibrium between the oxidized [Mn(III)(CB-TE1A)(OH)]^+^ and the reduced form of the complex [Mn(II)(CB-TE1A)(OH_2_)]^+^. The latter is thermodynamically unstable in aqueous solution, and thus, it dissociates with the formation of the Mn(II) aqueous ion. Monitoring the relaxivity values at low fields (0.01–10 MHz) as a function of time, a progressive relaxivity increase is observed over a period of 2 h, indicating that the complex dissociation is relatively slow. Addition of excess ascorbate (5 equiv) causes a further increase of relaxivity, confirming that the complex dissociates upon reduction (Figure 8).

The dissociation of the Mn(II) complex implies that the thermodynamic stability of the complex is low, which together with the high basicity of cross-bridge derivatives causes complex dissociation in water at neutral pH. The stability constant of the complex can be estimated using the structural descriptors published recently.^110^ Assuming a contribution of Δlog *K* = 4.1 for cyclam and a contribution of Δlog *K* = 2.7 for a carboxylate, we estimate a stability constant of log *K* = 6.8. These data must be taken with great caution as the presence of the bridging unit in the ligand is neglected for this estimation. However, this estimation supports that the Mn(II) complex has a low thermodynamic stability, in line with the dissociation of the complex observed in water. Noteworthily, the Mn(II) complex was prepared, though not isolated, to obtain the [Mn(III)(CB-TE1A)(OH)]^+^ complex using an organic solvent (*n*-BuOH).

Metal complexes with cross-bridge derivatives are often very inert, and thus, they can remain intact in aqueous media even if the complexes are not thermodynamically stable. A clear case of this behavior was observed for lanthanide(III) complexes with cyclam and cross-bridge cyclam ligands containing picolinate pendants. The cross-bridge derivative was found to be extremely inert, even in solutions with a high acid concentration (2 M HCl).^111^ However, the complexes with the nonreinforced ligand dissociated spontaneously in water, though they could be prepared in nonaqueous media.^112^ The results reported here demonstrate that the [Mn(II)(CB-TE1A)(OH_2_)]^+^ complex displays relatively fast dissociation kinetics, a behavior that is unprecedented for cross-bridge derivatives with transition metal ions. High-spin Mn(II) complexes lack any ligand field stabilization energy and often show fast ligand dissociation kinetics, though some examples of inert complexes have also been reported.^19^

## Conclusions

In this work, we have investigated a series of transition metal complexes with ligands based on the cross-bridge cyclam platform. This study evidenced that the small ligand cleft stabilizes high oxidation numbers. The Mn(III) derivatives investigated here display a rather intricate electrochemical behavior, with waves arising from the Mn^II^/Mn^III^ and Mn^III^/Mn^IV^ redox couples. The Mn(III) complexes are stabilized by the presence of a terminal hydroxide ligand. Reduction of the [Mn(III)(CB-TE1A)(OH)]^+^ complex to the Mn(II) derivative by ascorbate results in complex dissociation, as evidenced by relaxometric and EPR investigations. Cyclic voltammetry experiments also show that the ligands studied here stabilize Co(III), while the Ni(III) analogues can be detected using cyclic voltammetry at relatively low potentials in aqueous solutions. The [Ni(CB-TE2AM)]^2+^ complex provides significant CEST effects associated with the exchange of amide protons with bulk water. Overall, this study demonstrated that rigidified CB-cyclam derivatives represent an interesting platform to stabilize high oxidation states for transition metals (+III/+IV), a feature with great interest for developing redox-responsive agents or new catalysts. However, ligand design should be improved in order to increase the stability of the Mn(II) complex generated upon reduction of the parent Mn(III) derivative with ascorbate. The CEST response of the Ni(II) complex is also modest, most likely due to the rather symmetrical octahedral coordination environment, which results in slow electron relaxation.

## Experimental Section

### Materials and Methods

Reagents and solvents were commercial and used without further purification. 1,4,8,11-Tetraazabicyclo[6.6.2]hexadecane was purchased from CheMatech (Dijon, France). ^1^H and ^13^C NMR spectra were recorded at 25 °C on a Bruker Avance 300 MHz spectrometer or a Bruker Avance 500 MHz spectrometer. High-resolution electrospray-ionization time-of-flight (ESI-TOF) mass spectra were obtained in the positive mode using an LTQ-Orbitrap Discovery Mass Spectrometer coupled to a Thermo Accela HPLC system. Medium-performance liquid chromatography (MPLC) was carried out using a Puriflash XS 420 InterChim Chromatographer equipped with a UV-DAD detector using a reverse phase 15C18AQ column (60 Å, spherical 15 μm, 6 g) operating at a flow rate of 5 mL/min. Aqueous solutions were lyophilized using a Biobase BK-FD10 series apparatus. HPLC was carried out using a Jasco LC-4000 instrument equipped with a UV detector in the reverse phase using a Hypersil GOLD aQ column. Purity of complexes has been determined by analytical HPLC following the method described in Table S3 (Supporting Information).

The EPR spectra of the [Mn(CB-TE1A)(OH)]^+^ complex were measured on a JEOL FA200 ESR spectrometer, operating in the 9.45 GHz range in the X-band, with a modulation frequency of 100 kHz. The spectra were acquired at room temperature.

Electrochemical measurements were performed using an Autolab PGSTAT101 potentiostat working with a three-electrode configuration. The working electrode was a glassy carbon disc (Metrohm 6.1204.300), a Ag/AgCl reference electrode filled with 3 M KCl (Metrohm 6.0728.000) was used as the reference electrode, while a Pt wire was used as the counter electrode. Measurements were made with ca. 2 × 10^–3^ M solutions of complexes containing 0.15 M NaCl as the supporting electrolyte. The solutions were deoxygenated before each measurement by bubbling N_2_. The glassy carbon disc working electrode was polished before each experiment using a polishing kit (Metrohm 6.2802.010), first with α-Al_2_O_3_ (0.3 μm), and afterward washed with distilled water.

### CB-TE1AM

A solution of 2-chloroacetamide (0.0712 g, 0.761 mmol, 1.1 equiv) in CH_3_CN (20 mL) was added dropwise to a solution of 1,4,8,11-tetraazabicyclo[6.6.2]hexadecane (0.1567 g, 0.692 mmol, 1 equiv) in CH_3_CN (125 mL). The mixture was stirred at room temperature for 5 h. The solvent was evaporated, giving a yellow oil which was purified by MPLC using a reverse-phase C18AQ (6 g) column and H_2_O (0.1% HCOOH) and CH_3_CN (0.1% HCOOH) as the mobile phase (compound eluted at 100% H_2_O). The compound was lyophilized to afford a white solid (0.2195 g, 0.616 mmol, 89%). ^1^H NMR (400 MHz, D_2_O, pH 10.73): δ_H_ (ppm): 3.32–2.78 (m, 19H), 2.73–2.63 (m, 2H), 2.15–1.97 (m, 2H), 1.71–1.52 (m, 2H). ^13^C NMR (101 MHz, D_2_O, pH 10.73): δ_C_ (ppm) 175.31, 57.90, 57.84, 55.02, 54.97, 53.83, 49.84, 49.46, 47.58, 42.83, 22.40, 22.23. HR-MS (ESI^+^): *m*/*z* calcd for C_14_H_30_N_5_O [M + H]^+^, 284.2445; found, 284.2437. Elemental analysis: Calcd for C_14_H_29_N_5_O·2HCl: C, 47.19; H, 8.77; N, 19.65. Found: C, 47.84; H, 8.40; N, 19.04. IR (ATR, ν̃[cm^–1^]): 3351 ν (N–H), 1681 ν (C=O).

### CB-TE2AM

This compound was prepared following a slight modification of the synthesis reported in the literature.^68^ A solution of 1,4,8,11-tetraazabicyclo[6.6.2]hexadecane (0.2813 g, 1.243 mmol, 1 equiv) containing DIPEA (1.083 mL, 6.213 mmol, 5 equiv) in CH_3_CN (7 mL) was heated at 80 °C, and 2-chloroacetamide (0.2324 g, 2.485 mmol, 2 equiv) was added. The mixture was stirred and heated for 25 h. The solid was filtrated and washed with CH_3_CN (4 × 2 mL) and diethyl ether (2 × 2 mL), giving a beige solid (0.2475 g, 0.7269 mmol, 58%) ^1^H NMR (300 MHz, D_2_O, pH 10.13): δ_H_ (ppm): 3.55–3.30 (m, 4H), 3.21–2.90 (m, 12H), 2.80–2.56 (m, 8H), 2.13–1.92 (m, 2H), 1.6 (d, *J* = 15.6 Hz, 2H); ^13^C NMR (75 MHz, D_2_O, pH 10.13): δ_C_ (ppm) 177.91, 61.16, 58.27, 57.42, 55.74, 50.49, 50.19, 24.64. HR-MS (ESI^+^): *m*/*z* calcd for C_16_H_33_N_6_O_2_ [M + H]^+^, 341.2660; found, 341.2659. Elemental analysis: Calcd for C_16_H_32_N_6_O_2_·HCl: C, 50.98; H, 8.82; N, 22.30. Found: C, 50.45; H, 8.69; N, 22.49. IR (ATR, ν̃[cm^–1^]): 1674 ν (C=O).

### HCB-TE1A

This compound was prepared following the procedure described in the literature.^73^^1^H NMR (500 MHz, D_2_O, pH 2.90): δ_H_ (ppm): 4.85 (d, 1H), 3.72 (m, 1H), 3.75–3.10 (m, 12H), 2.96 (m, 5H), 2.80–2.50 (m, 4H), 2.40 (m, 2H), 1.73 (m, 2H); ^13^C NMR (126 MHz, D_2_O, pH 2.90): δ_C_ (ppm) 171.26, 58.01, 57.65, 57.56, 55.94, 55.48, 53.88, 49.41, 48.63, 48.30, 47.11, 41.49, 19.22, 18.23. HR-MS (ESI^+^): *m*/*z* calcd for C_14_H_29_N_4_O_2_ [M + H]^+^, 285.2285; found, 285.2279. Elemental analysis: Calcd for C_14_H_28_N_4_O_2_·2HBr: C, 37.68; H, 6.78; N, 12.56. Found: C, 38.38; H, 6.86; N, 12.61. IR (ATR, ν̃[cm^–1^]): 3398 ν (N–H), 1729 ν (C=O).

### H_2_CB-TE2A

**CB-TE2AM** (0.0440 g, 0.129 mmol) was dissolved in HCl 6 M (15 mL), and the mixture was refluxed for 4 days. The acid was removed, and water (3 mL) was added and evaporated. This procedure was repeated twice to remove most of the hydrochloric acid. The product was lyophilized, giving a yellowish oil (0.0421 g, 0.123 mmol, 95%). ^1^H NMR (300 MHz, D_2_O, pH 0.61): δ_H_ (ppm): 3.96 (d, *J* = 17.6 Hz, 2H), 3.65–2.87 (m, 22H), 2.48–2.28 (m, 2H), 1.78 (d, *J* = 16.9 Hz, 2H); ^13^C NMR (75 MHz, D_2_O, pH 0.61): δ_C_ (ppm) 172.68, 59.75, 57.70, 55.12, 53.19, 47.62, 47.35, 19.55. HR-MS (ESI^+^): *m*/*z* calcd for C_16_H_31_N_4_O_4_ [M + H]^+^, 343.2340; found, 343.2342.

### [Mn(CB-TE1AM)(OH)]Cl_2_

The **CB-TE1AM** ligand (0.0341 g, 0.120 mmol, 1 equiv) was dissolved in *n*-BuOH (3 mL) in the presence of DIPEA (21.0 μL, 0.120 mmol, 1 equiv). The solution was purged with an argon flow (about 10 min), and afterward, a solution of Mn(NO_3_)_2_·4H_2_O (0.0302 g, 0.120 mmol, 1 equiv) dissolved in *n*-BuOH (2 mL) was added. The reaction mixture was heated at 75 °C for 16 h. The solvent was evaporated under vacuum. Water (3 mL) was added, the solution was bubbled with O_2_ until it turned orange, and it was lyophilized. The orange oil was purified by MPLC using a reverse-phase C18AQ (6 g) column and H_2_O and CH_3_CN as the mobile phase (compound eluted at 100% H_2_O). The compound was lyophilized to afford an orange oil (0.0452 g, 0.094 mmol, 78%). HR-MS (ESI^+^): *m*/*z* calcd for [C_14_H_28_MnN_5_O]^+^, 337.1669; found, 337.1666. HPLC retention time: 3.22 min.

### [Mn(CB-TE1A)(OH)]Cl

The **HCB-TE1A** ligand (0.0504 g, 0.177 mmol, 1 equiv) was dissolved in *n*-BuOH (4 mL) in the presence of DIPEA (30.9 μL, 0.177 mmol, 1 equiv). The solution was purged with an argon flow, and afterward, a solution of Mn(NO_3_)_2_·4H_2_O (0.0448 g, 0.177 mmol, 1 equiv) dissolved in *n*-BuOH (2 mL) was added. The reaction mixture was heated at 90 °C for 24 h. The solvent was evaporated under vacuum. Water (3 mL) was added, the solution was bubbled with O_2_ until it turns orange, and it was lyophilized. The orange oil was purified by MPLC using a reverse-phase C18AQ (6 g) column and H_2_O and CH_3_CN as the mobile phase (compound eluted at 100% H_2_O). The compound was lyophilized to afford an orange oil (0.0167 g, 0.041 mmol, 23%). HR-MS (ESI^+^): *m*/*z* calcd for [C_14_H_26_MnN_4_O_2_]^+^, 337.1431; found, 337.1431. HPLC retention time: 3.13 min.

### [Ni(CB-TE2AM)]Cl_2_

The **CB-TE2AM** ligand (0.0552 g, 0.162 mmol, 1 equiv) was dissolved in *n*-BuOH (6 mL) in the presence of DIPEA (42.4 μ, 0.243 mmol, 1.5 equiv) with the assistance of an ultrasound bath. The solution was purged with an argon flow (about 10 min), and afterward, a solution of NiCl_2_ (0.0315 g, 0.243 mmol, 1.5 equiv) dissolved in *n*-BuOH (2 mL) was added. The reaction was maintained at 112 °C for 2 h. The reaction was stopped and allowed to cool down to room temperature. The reaction mixture was filtered through a cellulose filter (0.25 μm pore size), and the filtrate was concentrated in vacuum, giving a pink solid. The solid was purified by MPLC using a reverse-phase C18AQ (6 g) column. Eluting conditions: H_2_O/CH_3_CN, *v*/*v*, containing 0.1% TFA. The purification method was carried out in gradients of solvent B (CH_3_CN, 0 to 100%). The fractions containing the complex were combined, and they were lyophilized to furnish a pink solid (0.0862 g, 0.138 mmol, 85%). HR-MS (ESI^+^): *m*/*z* calcd for [C_16_H_32_N_6_NiO_2_]^2+^, 199.0965; found, 199.0965. HPLC retention time: 3.96 min.

### [Ni(CB-TE2A)]

The **H**_**2**_**CB-TE2A** ligand (0.0688 g, 0.201 mmol, 1 equiv) was dissolved in *n*-BuOH (6 mL) in the presence of DIPEA (42.0 μ, 0.241 mmol, 1.2 equiv). The solution was purged with an argon flow, and afterward, a solution of NiCl_2_ (0.0260 g, 0.201 mmol, 1 equiv) dissolved in *n*-BuOH (2 mL) was added. The reaction was maintained at 110 °C for 23 h. The reaction was stopped and allowed to cool down to room temperature. The reaction mixture was filtered, and the precipitate was washed with CH_2_Cl_2_ (2 × 2 mL), THF (1 × 2 mL), and diethyl ether (1 × 2 mL) giving a purple solid (0.0192 g, 0.048 mmol, 24%). HR-MS (ESI^+^): *m*/*z* calcd for [C_16_H_29_N_4_NiO_4_]^+^, 399.1537; found, 399.1538. HPLC retention time: 3.60 min.

### [Co(CB-TE2AM)]Cl_3_

Under an inert atmosphere, CoCl_2_ (0.0741 g, 0.3133 mmol, 2 equiv) was added over a deoxygenated suspension of the **CB-TE2AM** ligand (0.0530 g, 0.1557 mmol, 1 equiv) in *n*-BuOH (10 mL). The reaction mixture was heated to 112 °C for 2 h and allowed to cool down to room temperature, leading to a suspension. The addition of HCl (1.5 equiv) in H_2_O (3 mL) dissolved the blue precipitate and led to a red solution. Finally, the solvent was removed under reduced pressure, giving a blue solid, which was purified by MPLC using a C18AQ (6 g) column and H_2_O (0.1% TFA) and CH_3_CN (0.1% TFA) as the mobile phase (compound eluted at 100% H_2_O). The compound was lyophilized, giving a blue solid (0.0680 g, 0.134 mmol, 86%). ^1^H NMR (500 MHz, D_2_O, pH 3.76): δ_H_ (ppm): 4.29 (d, *J* = 17.9 Hz, 2H), 3.89 (d, *J* = 17.9 Hz, 2H), 3.44–3.31 (m, 2H), 3.30–3.21 (m, 2H), 3.02 (b, 1H), 2.78 (dt, *J* = 15.4, 3.2 Hz, 2H), 2.64 (dd, *J* = 14.4, 5.5 Hz, 2H), 2.57–2.41 (m, 8H), 2.07–1.99 (m, 4H), 1.90–1.81 (m, 2H), 1.78–1.69 (m, 2H). ^13^C NMR (101 MHz, D_2_O, pH 3.76): δ_C_ (ppm) 181.66, 69.10, 65.31, 58.26, 57.97, 57.07, 56.96, 22.24. HR-MS (ESI^+^): *m*/*z* calcd for [C_16_H_30_CoN_6_O_2_]^+^, 397.1757; found, 397.1755. HPLC retention time: 3.24 min.

### [Co(CB-TE2A)]Cl

The **H**_**2**_**CB-TE2A** ligand (0.0369 g, 0.108 mmol, 1 equiv) was dissolved in *n*-BuOH (6 mL) in the presence of DIPEA (29.0 μL, 0.162 mmol, 1.5 equiv). The yellow pale solution was purged with an argon flow and a solution of CoCl_2_·6H_2_O (0.0282 g, 0.118 mmol, 1.1 equiv) dissolved in *n*-BuOH (3 mL) was added. The reaction was maintained at 112 °C for 28 h, giving a blue precipitate. The reaction was stopped and allowed to cool down to room temperature. The reaction mixture was filtered, and the precipitate was washed with CHCl_3_ (2 × 2 mL). The addition of HCl (1.5 equiv) in H_2_O (3 mL) dissolved the blue precipitate and led to a red solution. Finally, the solvent was removed under reduced pressure, giving a blue solid, which was purified by MPLC using a C18AQ (6 g) column and H_2_O (0.1% TFA) and CH_3_CN (0.1% TFA) as the mobile phase (compound eluted at 100% H_2_O). The compound was lyophilized, giving a blue solid (0.0291 g, 0.067 mmol, 62%). ^1^H NMR (500 MHz, D_2_O, pH 7.40): δ_H_ (ppm): 4.21 (d, *J* = 17.6 Hz, 2H), 3.87–3.68 (m, 6H), 3.17 (t, *J* = 10.7 Hz, 2H), 3.08–2.73 (m, 11H), 2.61 (t, *J* = 12.0 Hz, 2H), 2.50–2.36 (m, 4H), 2.13 (d, *J* = 17.2 Hz, 2H); ^13^C NMR (101 MHz, D_2_O, pH 7.40): δ_C_ (ppm) 182.13, 68.92, 65.17, 58.14, 57.98, 57.30, 56.63, 22.07. HR-MS (ESI^+^): *m*/*z* calcd for [C_16_H_28_CoN_4_O_4_]^+^, 399.1437; found, 399.1438. HPLC retention time: 3.13 min.

### Crystal Structure Determinations

Crystallographic data and the structure refinement parameters are given in Tables S4 and S5. The single crystals of [Ni(CB-TE2AM)]Cl_2_·2H_2_O and [Mn(CB-TE1AM)(OH)](PF_6_)_2_ were analyzed by X-ray diffraction. Measurements were performed at 100 K, using a Bruker D8 Venture Photon II CMOS detector and Cu-Kα radiation (λ = 1.54178 Å) for the nickel complex and Mo-Kα radiation (λ = 0.71073 Å) in the case of the manganese complex generated by an Incoatec high brilliance microfocus source equipped with Incoatec Helios multilayer optics. The software APEX3^113^ was used for collecting frames of data, indexing reflections, and the determination of lattice parameters; SAINT^114^ was used for integration of the intensity of reflections; and SADABS^115^ was used for scaling and empirical absorption correction. The structure was solved by dual-space methods using the SHELXT program.^116^ All non-hydrogen atoms were refined with anisotropic thermal parameters by full-matrix least-squares calculations on *F*^2^ with the SHELXL-2018/3^117^ program. Most hydrogen atoms of the compound were inserted at calculated positions and constrained with isotropic thermal parameters, except for the H atoms of the water molecules in both complexes and the hydrogen atom of the hydroxyl group for the Mn complex, which were located from a Fourier difference map and refined isotropically. These data can be obtained free of charge from the Cambridge Crystallographic Data Centre via www.ccdc.cam.ac.uk/data_request/cif.

### CEST Measurements

CEST measurements were recorded on a Bruker AVIII 500 spectrometer equipped with a 5 mm probe and a standard temperature control unit. The CEST experiments were carried out at 298 and 310 K on the [Ni(CB-TE2AM)]^2+^ complex 7 mM at pH 7 in H_2_O with 10% D_2_O. The sample was irradiated with a presaturation RF pulse of length = 2 s and strengths of 2.5, 5, 10, 15, 20, and 25 μT in the frequency range between 100 and −100 ppm, in increments of 1.0, 0.5, and 0.2 ppm, depending on the frequency region. The recycle delay was set to 2.5 s. The signal intensity of bulk water recorded as a function of the presaturation frequency was normalized (M_*z*_/M_0_) and plotted against the saturation offset (ppm), relative to the bulk water resonance frequency, to obtain the CEST spectrum.

### Relaxometric Measurements

^1^H NMRD profiles were acquired in the ^1^H Larmor frequency ranging from 0.01 to 120 MHz using a fast field cycling Stelar SmarTracer relaxometer operating at low field strengths (9.97 × 10^–3^–10 MHz) and a high field Stelar relaxometer operating between 20 and 120 MHz. The fast-field cycling relaxometer is equipped with a silver magnet, while the high field relaxometer is equipped with an HTS-110 3T Metrology cryogen-free superconducting magnet. The temperature was controlled with a Stelar VTC-91 heater airflow equipped with a copper-constantan thermocouple (uncertainty of ±0.1 K). The measurements were performed using the standard inversion recovery sequence with a typical 90° RF pulse of 3.5 μs. The reproducibility of the data was within ±0.5%.

### Kinetic Experiments

Kinetic reducing reactions of the [Mn(CB-TE1A)(OH)]^+^ complex by ascorbate were investigated by following the complex absorption band (wavelength around 320 nm) using both the buffer (phosphate, ∼ 0.1 M, or tris-buffer, 0.05 M) and the ascorbate concentration (1 to 4 mM) in high excess relative to the complex concentration (∼10^–4^ M).

Slow reactions were monitored by a Kontron-Uvikon 942 UV–vis spectrophotometer at 25 °C and 1 cm path length quartz cuvettes, with the complex being the last reagent added to the sample reaction. The fast reactions were monitored on a Bio-Logic SFM-20 stopped-flow system interfaced with a computer and operated by Bio-Kine 32 (v4.51) software, which controls both the data acquisition and analysis. One syringe contained the complex at the same ionic strength and buffer concentration as that of the ascorbate introduced in the second syringe. Each experiment was taken in triplicate.

In every case, absorbance (*A*) versus time (*t*) curves were appropriately fitted by a first-order integrated rate eq 1, with *A*_o_, *A*_*t*_, and *A*_∞_ being the absorbance values at times zero, *t*, and at the end of the reaction, respectively, and *k*_0_ being the calculated pseudo-first-order rate constant.
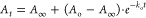
1

## Supporting Information Available

NMR spectra, absorption spectra, ^1^H–^1^H COSY spectra, ^1^H–^13^C HSQC and HMBC spectra, Z spectra, plots of the linear dependence of anodic and cathodic peak currents with the square root of the scan rate of the complexes, cyclic voltammograms, pseudo-first-order rate constants for the reaction of [Mn(CB-TE1A)(OH)]^+^ with ascorbate in phosphate buffer (0.1 M; *I* = 0.12 M NaCl) as a function of pH, EPR spectra, mass spectra, HPLC chromatograms, crystal data and structure refinement details of [Ni(CB-TE2AM)]Cl_2_·2H_2_O and [Mn(CB-TE1AM)(OH)](PF_6_)_2_, parameters of the three-pool system BM fit of [Ni(CB-TE2AM)]^2+^, and parameters of the tow-pool system BM fit of [Ni(CB-TE2AM)]^2+^ (PDF)
