# Supplementary material for: Endeavor toward Redox-Responsive Transition Metal Contrast Agents Based on the Cross-Bridge Cyclam Platform
Source: Inorg Chem. 2024 Jan 10;63(3):1575–88. doi: 10.1021/acs.inorgchem.3c03486 (PMC10806912; doi:10.1021/acs.inorgchem.3c03486)
Supplement: Supplementary file 1 — ic3c03486_si_001.pdf [file ic3c03486_si_001.pdf]

Supporting Information for:

Endeavor toward Redox-Responsive Transition Metal Contrast Agents Based on the Cross-Bridge Cyclam Platform

Rocío Uzal-Varela,[†] Aurora Rodríguez-Rodríguez,[†] Daniela Lalli,*[‡] Laura Valencia,[§] Marcelino Maneiro,[||] Mauro Botta,[‡] Emilia Iglesias,[†] David Esteban-Gómez,[†] Goran Angelovski[⊥] and Carlos Platas-Iglesias*[†]

[†] Universidade da Coruña, Centro Interdisciplinar de Química e Bioloxía (CICA) and Departamento de Química, Facultade de Ciencias, 15071, A Coruña, Galicia, Spain.

[‡] Universitá del Piemonte Orientale, Dipartimento di Scienze e Innovazione Tecnologica, Magnetic Resonance Platform (PRISMA-UPO), Viale T. Michel 11, 15121 Alessandria, Italy

[§] Departamento de Química Inorgánica, Facultad de Ciencias, Universidade de Vigo, As Lagoas, Marcosende, 36310 Pontevedra, Spain.

[||] Departamento de Química Inorgánica, Facultade de Ciencias, Campus Terra, Universidade de Santiago de Compostela 27002 Lugo, Galicia, Spain

[⊥] Laboratory of Molecular and Cellular Neuroimaging, International Center for Primate Brain Research (ICPBR), Center for Excellence in Brain Science and Intelligence Technology (CEBSIT), Chinese Academy of Sciences (CAS), Shanghai 201602, PR China

Email: carlos.platas.iglesias@udc.es
E-mail: daniela.lalli@uniupo.it

Summary

Figure S1. ^{13}C NMR spectrum of the $[Co(CB\text{-}TE2AM)]Cl_3$ synthesized in the presence of DIPEA (126 MHz, D_2O, pH 3.87, 298 K). The spectrum evidences the presence of two species in solution. 5

Figure S2. Blue line: Absorption spectrum of a 2.05 mM solution of the [Ni(CB-TE2A)] complex recorded at 298 K, pH 6.94. Purple line: Absorption spectrum of a 2.15 mM solution of the $[Ni(CB\text{-}TE2AM)]^{2+}$ complex recorded at 298 K, pH 7.23. 6

Figure S3. Blue line: Absorption spectrum of a 1.66 mM solution of the [Co(CB-TE2A)]Cl complex recorded at 298 K, pH 7.40. Purple line: Absorption spectrum of a 2.08 mM solution of the $[Co(CB\text{-}TE2AM)]Cl_3$ complex recorded at 298 K, pH 6.88. 7

Figure S4. Absorption spectra of a 2.19 mM solution of the $[Mn(CB\text{-}TE1AM)(OH)]^{2+}$ complex recorded in the pH range 3.06 - 5.57. 7

Figure S5. ^{1}H - ^{1}H COSY spectrum of $[Co(CB\text{-}TE2AM)]^{3+}$ (500 MHz, D_2O, pH 3.76, 298 K). 8

Figure S6. HSQC ^{1}H - ^{13}C spectrum of $[Co(CB\text{-}TE2AM)]^{3+}$ (500 MHz, D_2O, pH 3.76, 298 K). 8

Figure S7. HMBC ^{1}H - ^{13}C spectrum of $[Co(CB\text{-}TE2AM)]^{3+}$ (500 MHz, D_2O, pH 3.76, 298 K). 9

Figure S8. ^{1}H - ^{1}H COSY spectrum of $[Co(CB\text{-}TE2A)]^{+}$ (500 MHz, D_2O, pH 7.40, 298 K). 10

Figure S9. HSQC ^{1}H - ^{13}C spectrum of $[Co(CB\text{-}TE2A)]^{+}$ (500 MHz, D_2O, pH 7.40, 298 K). 10

Figure S10. HMBC ^{1}H - ^{13}C spectrum of $[Co(CB\text{-}TE2A)]^{+}$ (500 MHz, D_2O, pH 7.40, 298 K). 11

Figure S11. ^{1}H NMR spectra of the $[Ni(CB\text{-}TE2AM)]^{2+}$ complex dissolved in pure water (black trace) and in D_2O (red trace). The signals of the exchangeable sites appearing in pure water and absent in D_2O are indicated with an asterisk (*). 12

Figure S12. Z-spectra of $[Ni(CB\text{-}TE2AM)]^{2+}$ complex acquired at 298 K, recorded using different saturation powers B_1 (11.75 T, saturation time 2 s). Experimental points are presented with open circles, while the fits obtained from a 3-pool model using the Bloch-McConnell equations are presented with lines. 13

Figure S13. Z-spectra of $[Ni(CB\text{-}TE2AM)]^{2+}$ complex acquired at 310 K, recorded using different saturation powers B_1 (11.75 T, saturation time 2 s). Experimental points are presented with open circles, while the fits obtained from a 2-pool model using the Bloch-McConnell equations are presented with lines. 14

Figure S14. Plot of the linear dependence of anodic and cathodic peak currents with the square root of the scan rate of [Co(CB-TE2AM]$^{3+}$ (purple) and [Co(CB-TE2A]$^{+}$ (blue) complexes.. 15

Figure S15. Plot of the linear dependence of anodic and cathodic peak currents with the square root of the scan rate of [Ni(CB-TE2AM]$^{2+}$ (purple) and [Ni(CB-TE2A] (blue) complexes.. 15

Figure S16. Cyclic voltammogram of the [Mn(CB-TE1AM)(OH)]$^{2+}$ complex in aqueous solution in 0.15 M NaCl (2.19 mM, pH 7.01) recorded at 10, 50, 100, 250 and 500 mV·s^{-1}. 16

Figure S17. Red dashed line: absorption spectrum of a 1.25·10^{-3} M solution of the [Co(CB-TE2AM)]$^{3+}$ complex. Solid lines: absorption spectra of the complex in the presence of ascorbic acid (1.33·10^{-3} M). Dashed lines: absorption spectra of the complex in the presence of ascorbic acid (1.96·10^{-3} M). 17

Figure S18. Blue line: Absorption spectrum of a 1.15·10^{-4} M solution of the [Mn(CB-TE1A)(OH)]$^{+}$ complex recorded at 298 K, pH 7.4. Purple line: Absorption spectrum of a 9.31·10^{-5} M solution of the [Mn(CB-TE1A)(OH)]$^{+}$ complex in the presence of ascorbate recorded at 298 K, pH 7.4. 17

Figure S19. Pseudo-first-order rate constants for the reaction of [Mn(CB-TE1A)(OH)]$^{+}$ with ascorbate in phosphate buffer (0.1 M; *I* = 0.12 M NaCl) as a function of pH. The inset indicates the concentrations of ascorbate. 18

Figure S20. EPR spectrum (X-band) recorded for the [Mn(CB-TE1A)(OH_2)]$^{2+}$ complex at 298 K. 19

Figure S21. ^{1}H NMR spectrum of CB-TE1AM (400 MHz, D_2O, pH 10.73, 298 K). 20

Figure S22. ^{13}C NMR spectrum of CB-TE1AM (101 MHz, D_2O, pH 10.73, 298 K). 20

Figure S23. Experimental high resolution mass spectrum (ESI^{+}) of CB-TE1AM. 21

Figure S24. ^{1}H NMR spectrum of CB-TE2AM (300 MHz, D_2O, pH 10.13, 298 K). 22

Figure S25. ^{13}C NMR spectrum of CB-TE2AM (75 MHz, D_2O, pH 10.13, 298 K). 22

Figure S26. Experimental high resolution mass spectrum (ESI^{+}) of CB-TE2AM. 23

Figure S27. ^{1}H NMR spectrum of H_2CB-TE2A (300 MHz, D_2O, pH 0.61, 298 K). 24

Figure S28. ^{13}C NMR spectrum of H_2CB-TE2A (75 MHz, D_2O, pH 0.61, 298 K). 24

Figure S29. Experimental high resolution mass spectrum (ESI^{+}) of H_2CB-TE2A. 25

Figure S30. Experimental high resolution mass spectrum (ESI^{+}) of [Mn(CB-TE1AM)(OH)]Cl_2. 26

Figure S31. HPLC chromatogram of [Mn(CB-TE1AM)(OH)]Cl_2 at 400 nm. 26

Figure S32. Experimental high resolution mass spectrum (ESI^+) of [Mn(CB-TE1A)(OH)]Cl. 27

Figure S33. HPLC chromatogram of [Mn(CB-TE1A)(OH)]Cl at 400 nm........ 27

Figure S34. Experimental high resolution mass spectrum (ESI^+) of [Ni(CB-TE2AM)]Cl_2 28

Figure S35. HPLC chromatogram of [Ni(CB-TE2AM)]Cl_2 at 400 nm. 28

Figure S36. Experimental high resolution mass spectrum (ESI^+) of [Ni(CB-TE2A)]. 29

Figure S37. HPLC chromatogram of [Ni(CB-TE2A)] at 517 nm. 29

Figure S38. ^1H NMR spectrum of [Co(CB-TE2AM)]Cl_3 (500 MHz, D_2O, pH 3.76, 298 K). 30

Figure S39. ^{13}C NMR spectrum of [Co(CB-TE2AM)]Cl_3 (101 MHz, D_2O, pH 3.76, 298 K). 30

Figure S40. Experimental high resolution mass spectrum (ESI^+) of [Co(CB-TE2AM)]Cl_3. 31

Figure S41. HPLC chromatogram of [Co(CB-TE2AM)]Cl_3 at 352 nm........ 31

Figure S42. ^1H NMR spectrum of [Co(CB-TE2A)]Cl (500 MHz, D_2O, pH 7.40, 298 K). 32

Figure S43. ^{13}C NMR spectrum of [Co(CB-TE2A)]Cl (101 MHz, D_2O, pH 7.40, 298 K). ... 32

Figure S44. Experimental high resolution mass spectrum (ESI^+) of [Co(CB-TE2A)]Cl. ... 33

Figure S45. HPLC chromatogram of [Co(CB-TE2A)]Cl at 355 nm. 33

Table S1. Assignment of the ^1H and ^{13}C spectra of the $[Co(CB\text{-}TE2AM)]^{3+}$ complex. 34

Table S2. Assignment of the ^1H and ^{13}C spectra of the $[Co(CB\text{-}TE2A)]^{+}$ complex. 34

Table S3. HPLC analytical method. 35

Table S4. Crystal data and structure refinement details of [Ni(CB-TE2AM)]$Cl_2{\cdot}2H_2O$... 35

Table S5. Crystal data and structure refinement details of [Mn(CB-TE1AM)(OH)]$(PF_6)_2$. 36

Table S6. Parameters of the three-pool system BM fit of $[Ni(CB\text{-}TE2AM)]^{2+}$ at 298 K.[a] 36

Table S7. Parameters of the two-pool system BM fit of $[Ni(CB\text{-}TE2AM)]^{2+}$ at 310 K.[a] .. 37

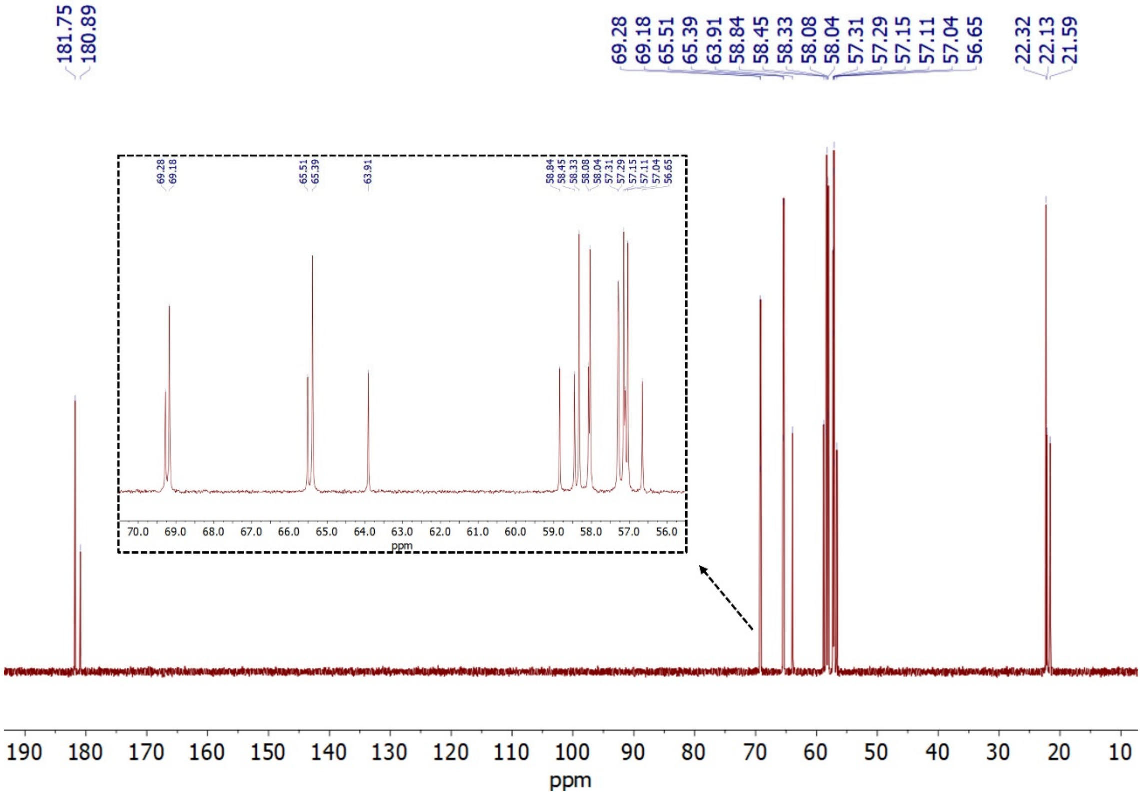

Figure S1. ^{13}C NMR spectrum of the $[Co(CB\text{-}TE2AM)]Cl_3$ synthesized in the presence of DIPEA (126 MHz, D_2O, pH 3.87, 298 K). The spectrum evidences the presence of two species in solution.

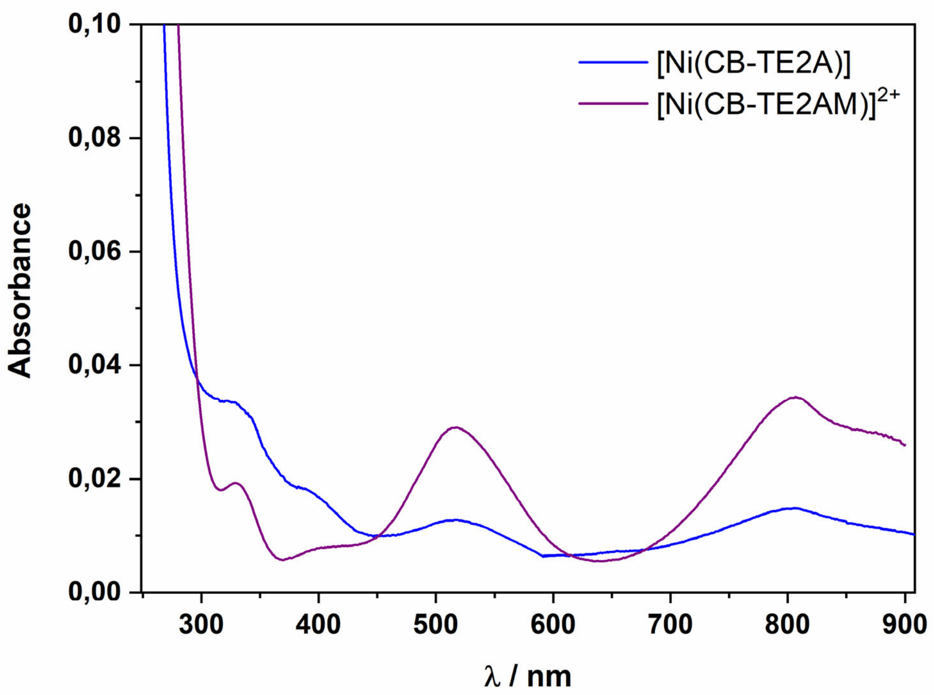

Figure S2. Blue line: Absorption spectrum of a 2.05 mM solution of the [Ni(CB-TE2A)] complex recorded at 298 K, pH 6.94. Purple line: Absorption spectrum of a 2.15 mM solution of the $[Ni(CB\text{-}TE2AM)]^{2+}$ complex recorded at 298 K, pH 7.23.

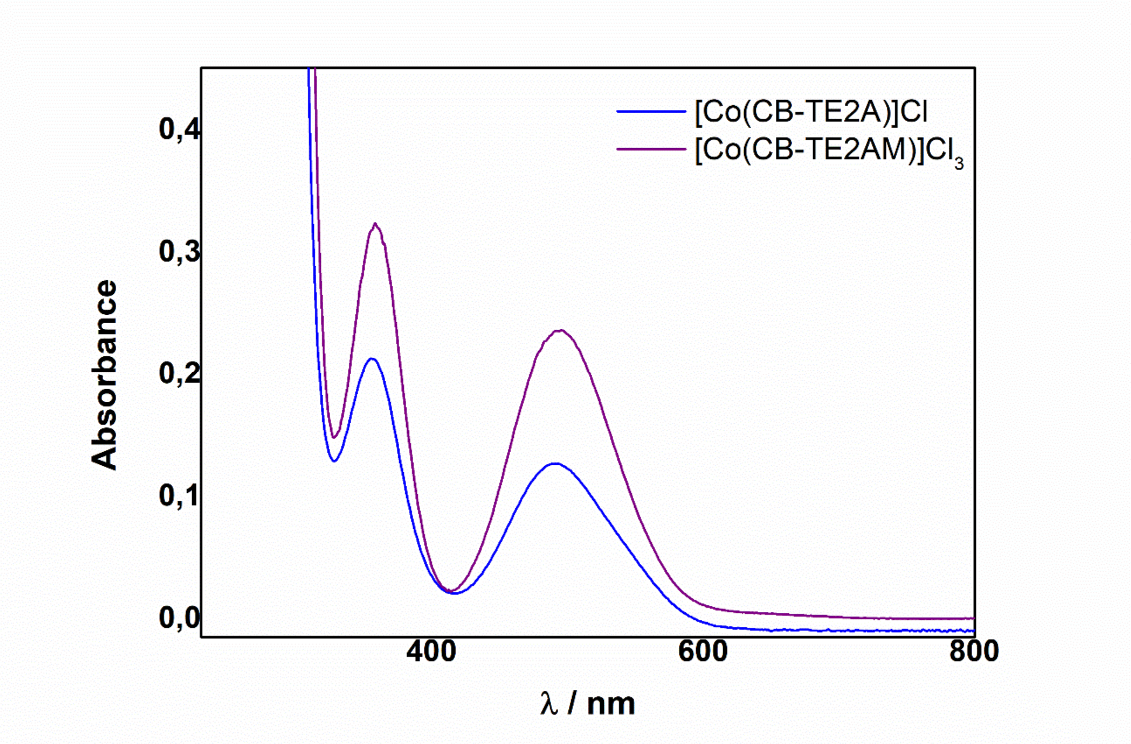

Figure S3. Blue line: Absorption spectrum of a 1.66 mM solution of the [Co(CB-TE2A)]Cl complex recorded at 298 K, pH 7.40. Purple line: Absorption spectrum of a 2.08 mM solution of the [Co(CB-TE2AM)]Cl_3 complex recorded at 298 K, pH 6.88.

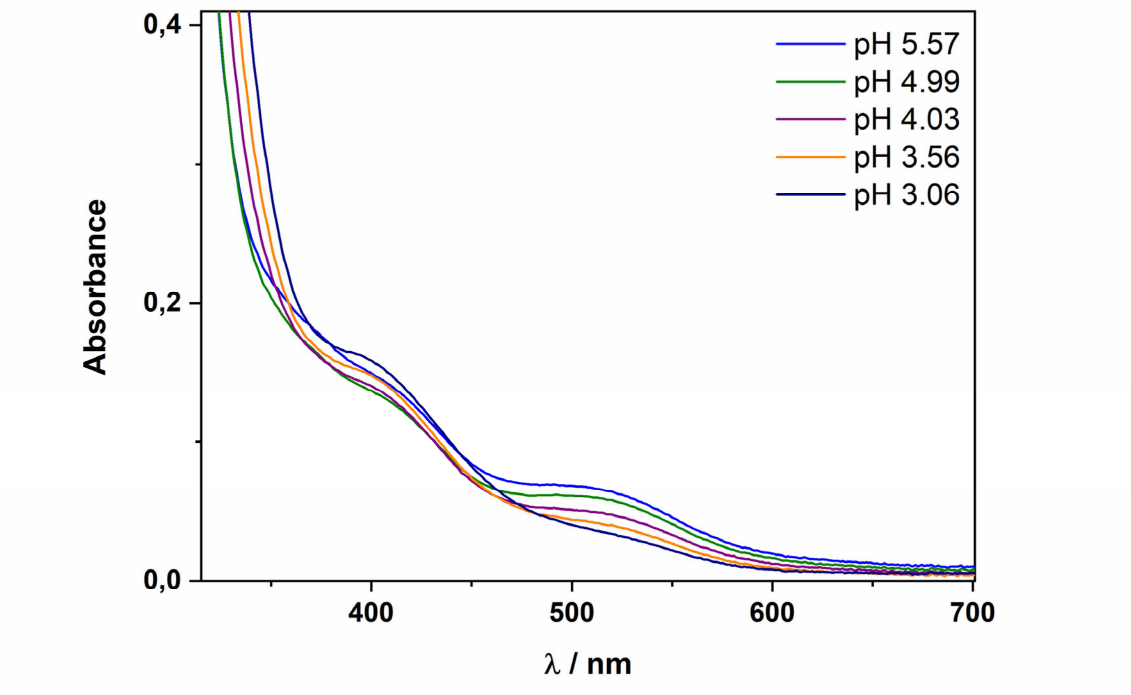

Figure S4. Absorption spectra of a 2.19 mM solution of the $[Mn(CB-TE1AM)(OH)]^{2+}$ complex recorded in the pH range 3.06 - 5.57.

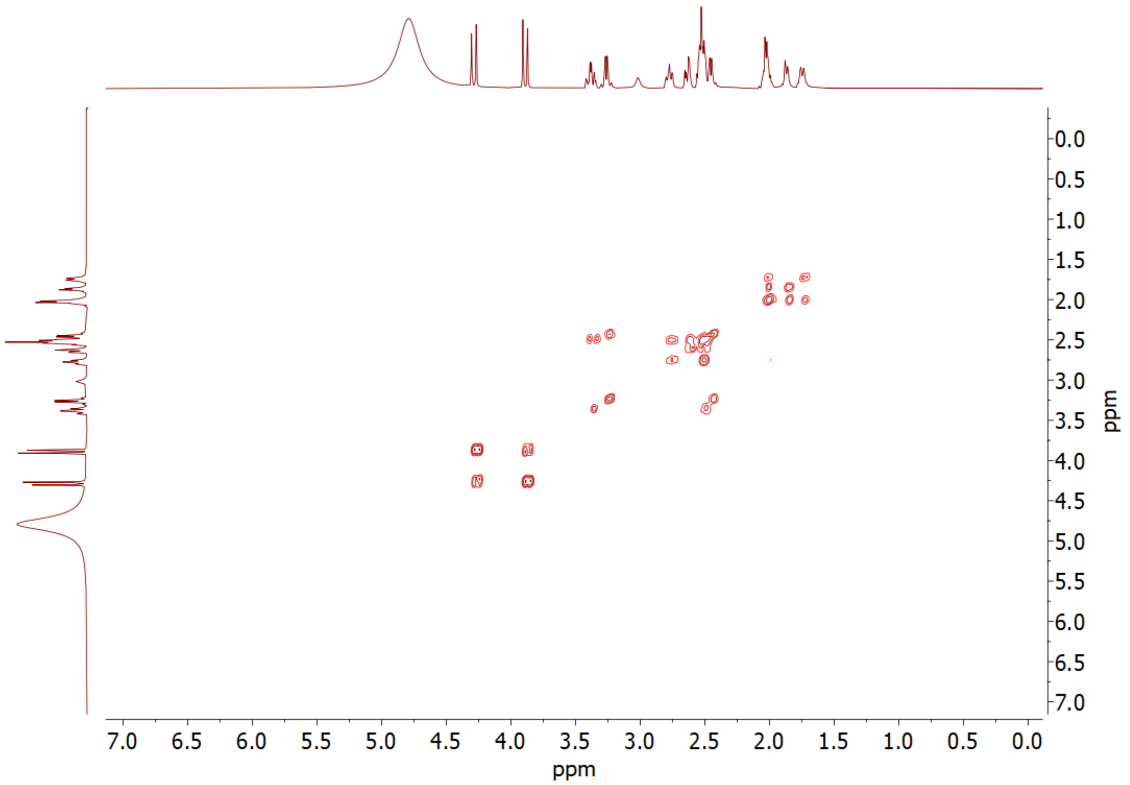

Figure S5. ^{1}H - ^{1}H COSY spectrum of $[Co(CB\text{-}TE2AM)]^{3+}$ (500 MHz, D_2O, pH 3.76, 298 K).

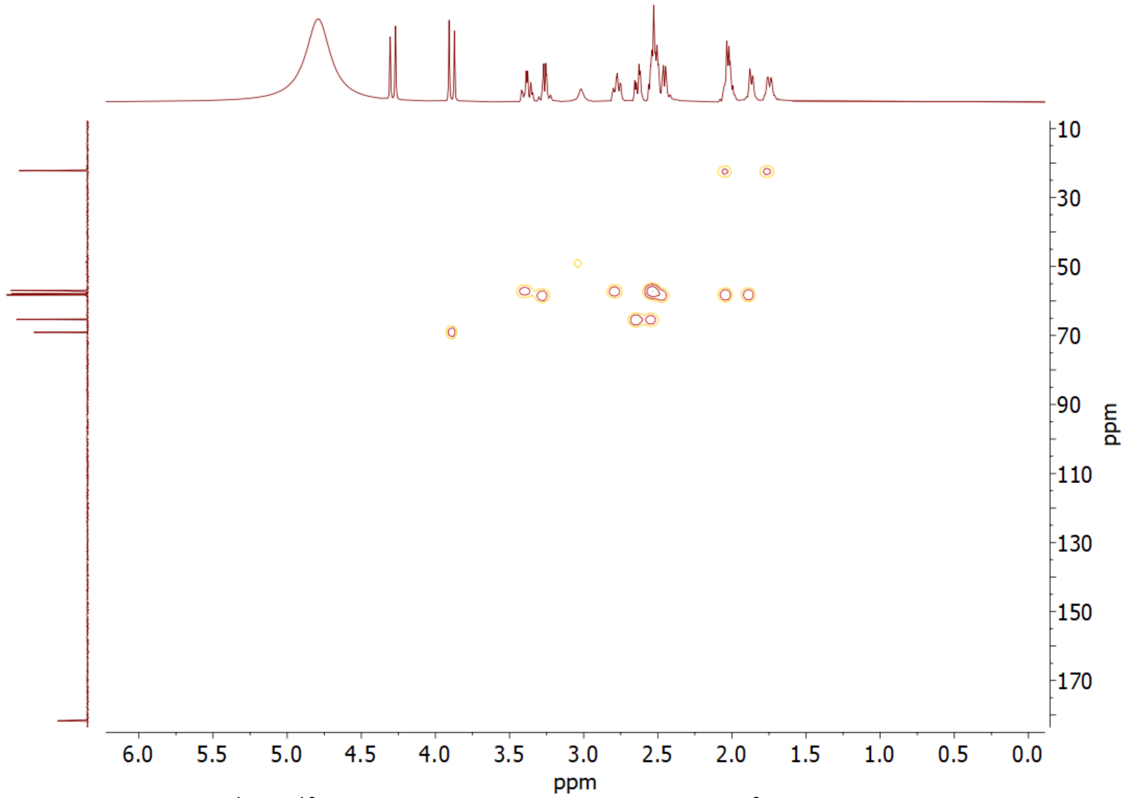

Figure S6. HSQC ^{1}H - ^{13}C spectrum of $[Co(CB\text{-}TE2AM)]^{3+}$ (500 MHz, D_2O, pH 3.76, 298 K).

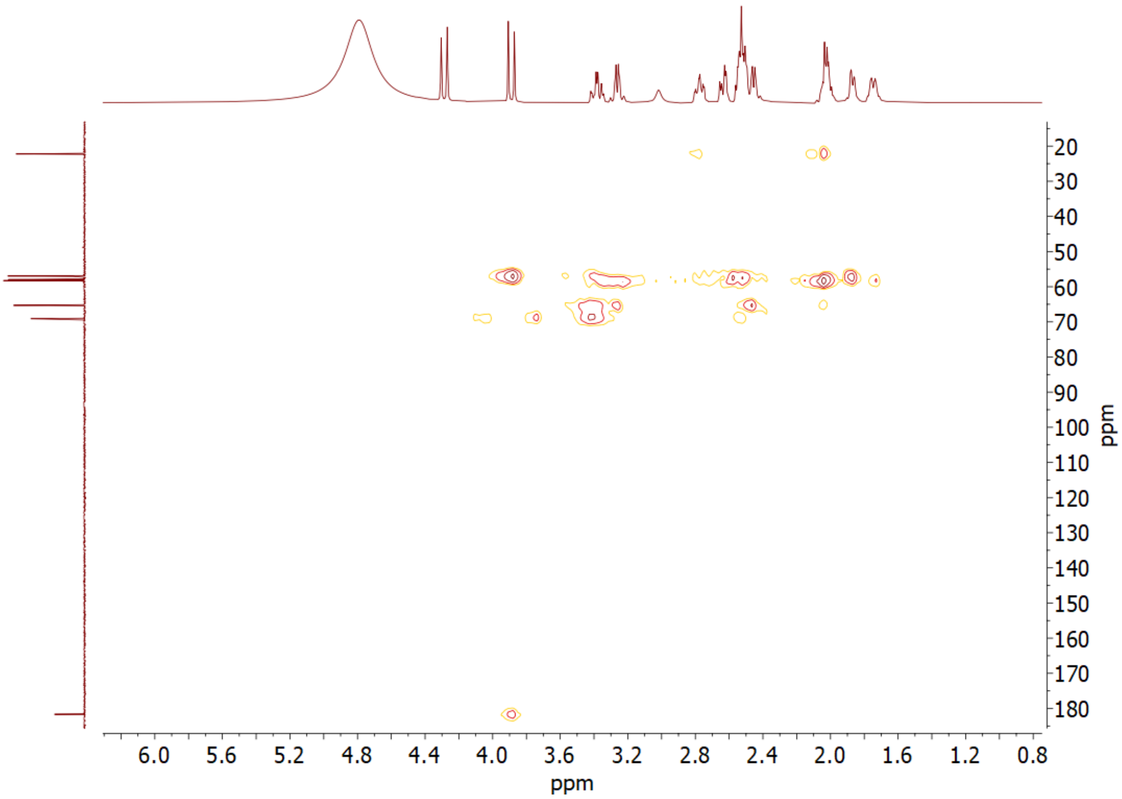

Figure S7. HMBC ^{1}H - ^{13}C spectrum of $[Co(CB\text{-}TE2AM)]^{3+}$ (500 MHz, D_2O, pH 3.76, 298 K).

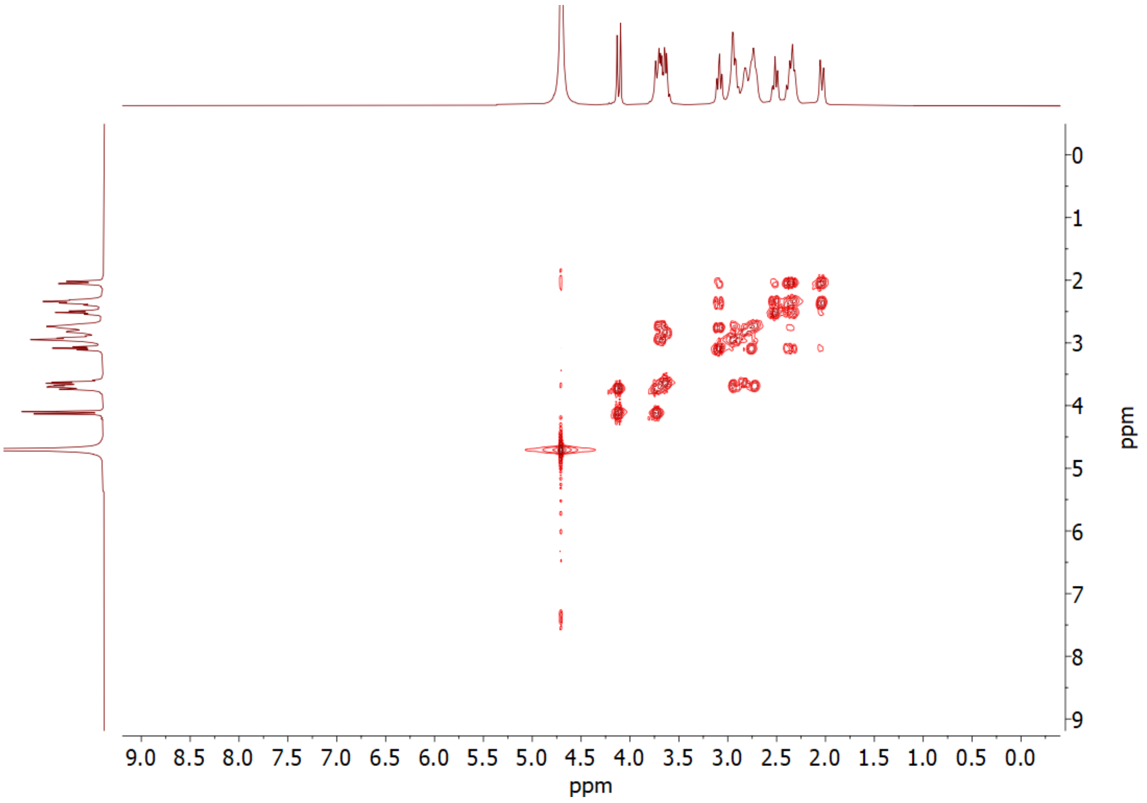

Figure S8. ^{1}H - ^{1}H COSY spectrum of $[Co(CB\text{-}TE2A)]^{+}$ (500 MHz, D_2O, pH 7.40, 298 K).

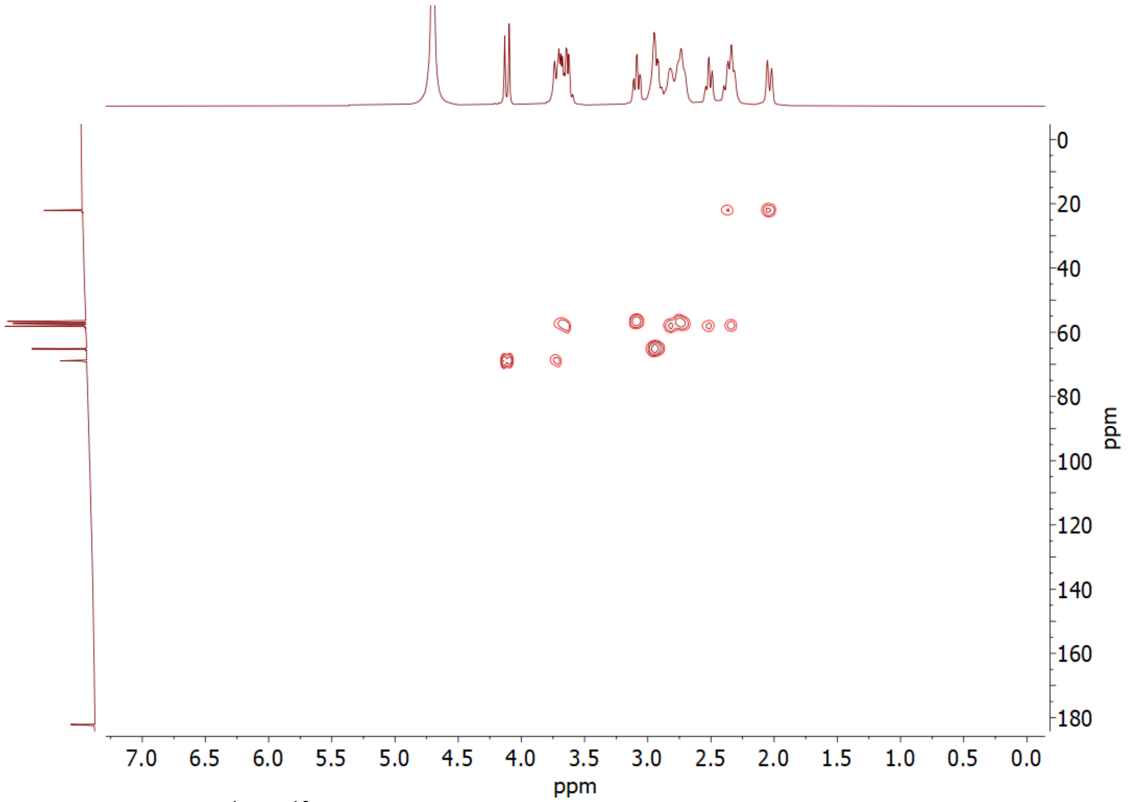

Figure S9. HSQC ^{1}H - ^{13}C spectrum of $[Co(CB\text{-}TE2A)]^{+}$ (500 MHz, D_2O, pH 7.40, 298 K).

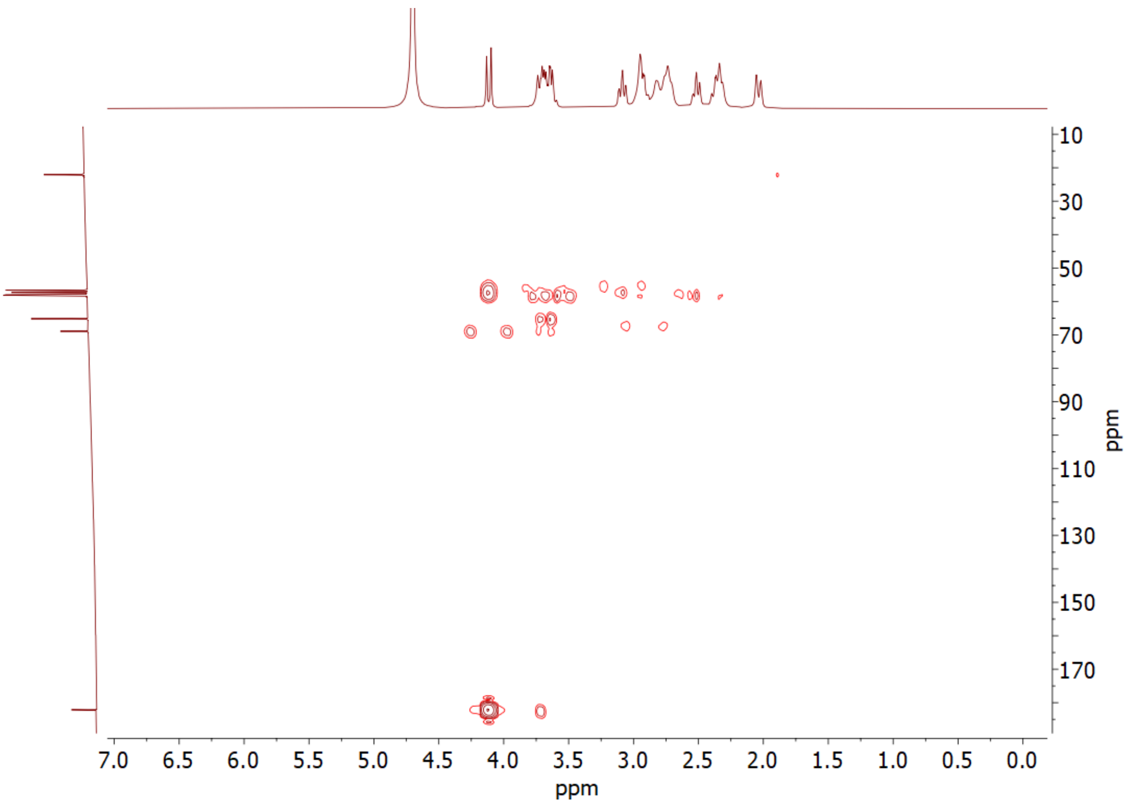

Figure S10. HMBC ^{1}H - ^{13}C spectrum of $[Co(CB\text{-}TE2A)]^{+}$ (500 MHz, D_2O, pH 7.40, 298 K).

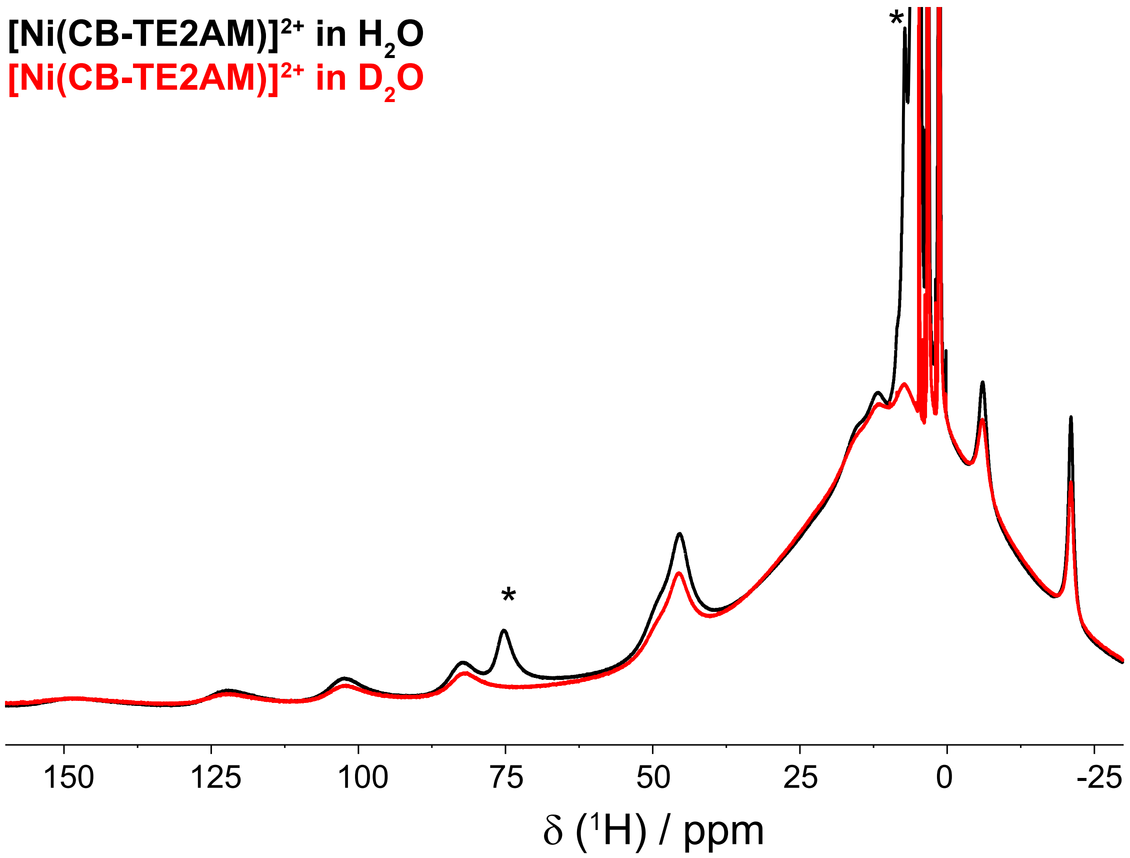

Figure S11. ^{1}H NMR spectra of the $[Ni(CB\text{-}TE2AM)]^{2+}$ complex dissolved in pure water (black trace) and in D_2O (red trace). The signals of the exchangeable sites appearing in pure water and absent in D_2O are indicated with an asterisk (*).

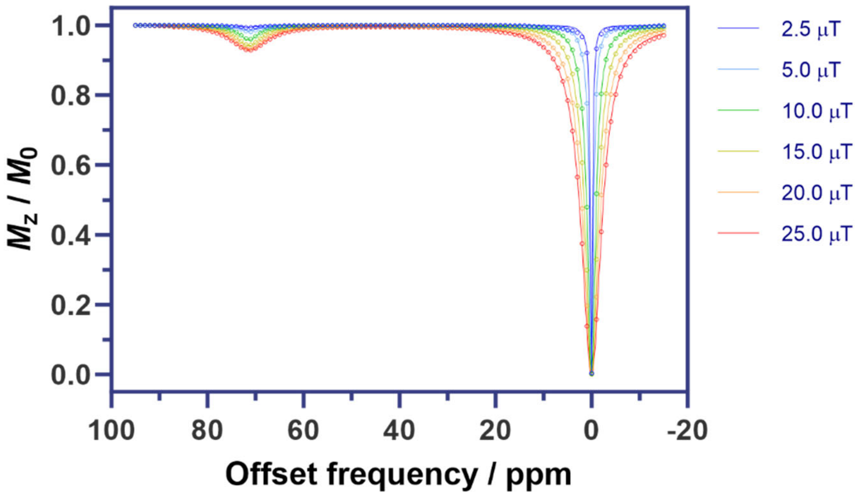

Figure S12. Z-spectra of $[Ni(CB\text{-}TE2AM)]^{2+}$ complex acquired at 298 K, recorded using different saturation powers B_1 (11.75 T, saturation time 2 s). Experimental points are presented with open circles, while the fits obtained from a 3-pool model using the Bloch-McConnell equations are presented with lines.

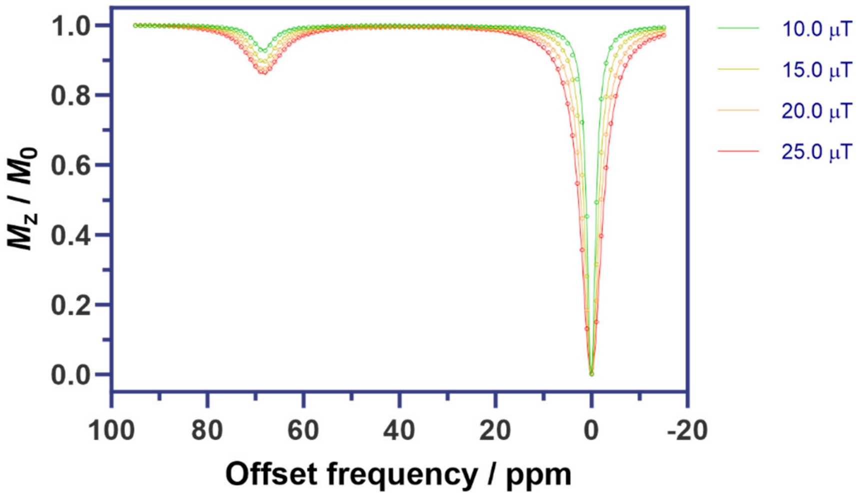

Figure S13. Z-spectra of $[Ni(CB\text{-}TE2AM)]^{2+}$ complex acquired at 310 K, recorded using different saturation powers B_1 (11.75 T, saturation time 2 s). Experimental points are presented with open circles, while the fits obtained from a 2-pool model using the Bloch-McConnell equations are presented with lines.

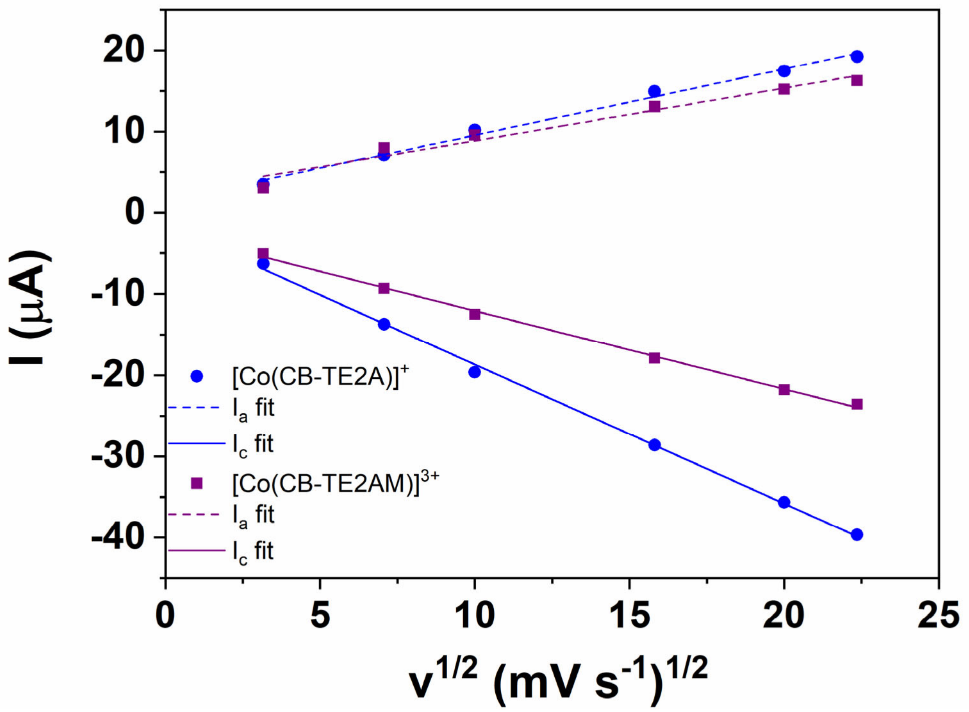

Figure S14. Plot of the linear dependence of anodic and cathodic peak currents with the square root of the scan rate of $[Co(CB\text{-}TE2AM]^{3+}$ (purple) and $[Co(CB\text{-}TE2A]^{+}$ (blue) complexes.

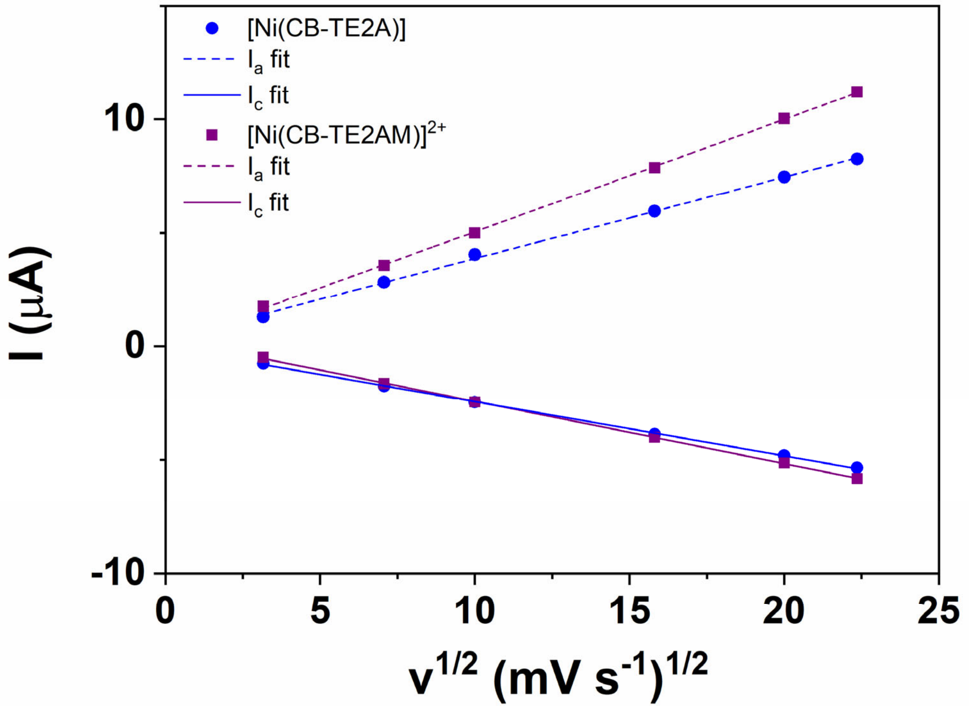

Figure S15. Plot of the linear dependence of anodic and cathodic peak currents with the square root of the scan rate of $[Ni(CB\text{-}TE2AM]^{2+}$ (purple) and [Ni(CB-TE2A] (blue) complexes.

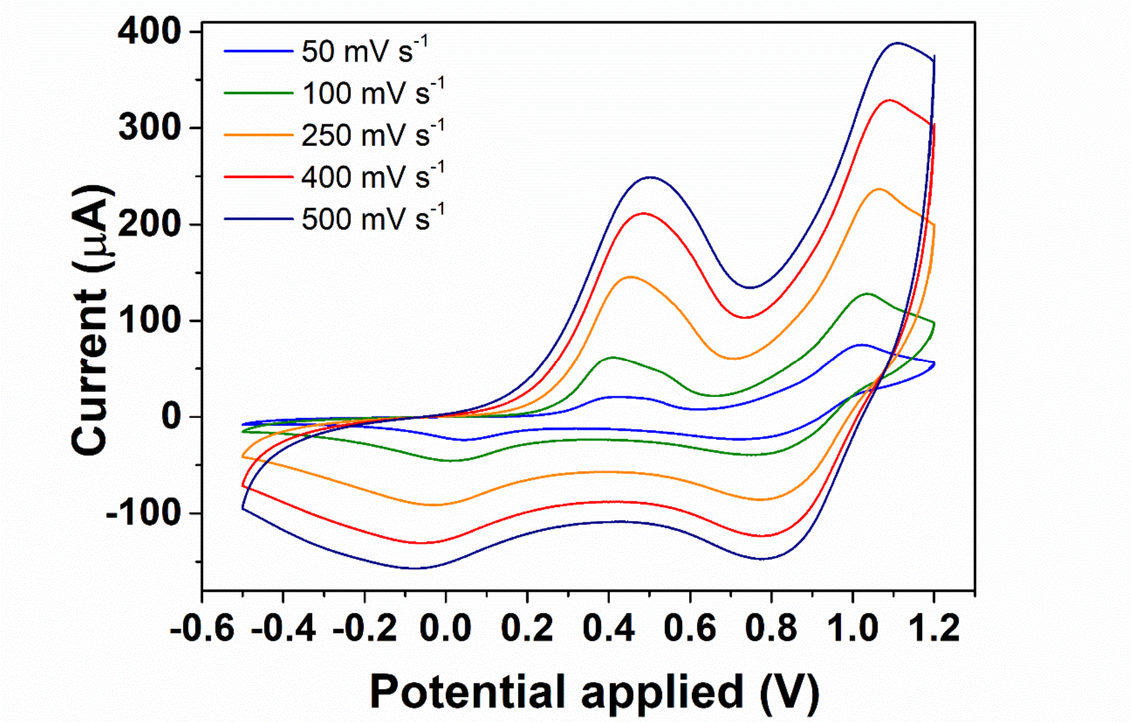

Figure S16. Cyclic voltammogram of the $[Mn(CB\text{-}TE1AM)(OH)]^{2+}$ complex in aqueous solution in 0.15 M NaCl (2.19 mM, pH 7.01) recorded at 10, 50, 100, 250 and 500 mV·s^{-1}.

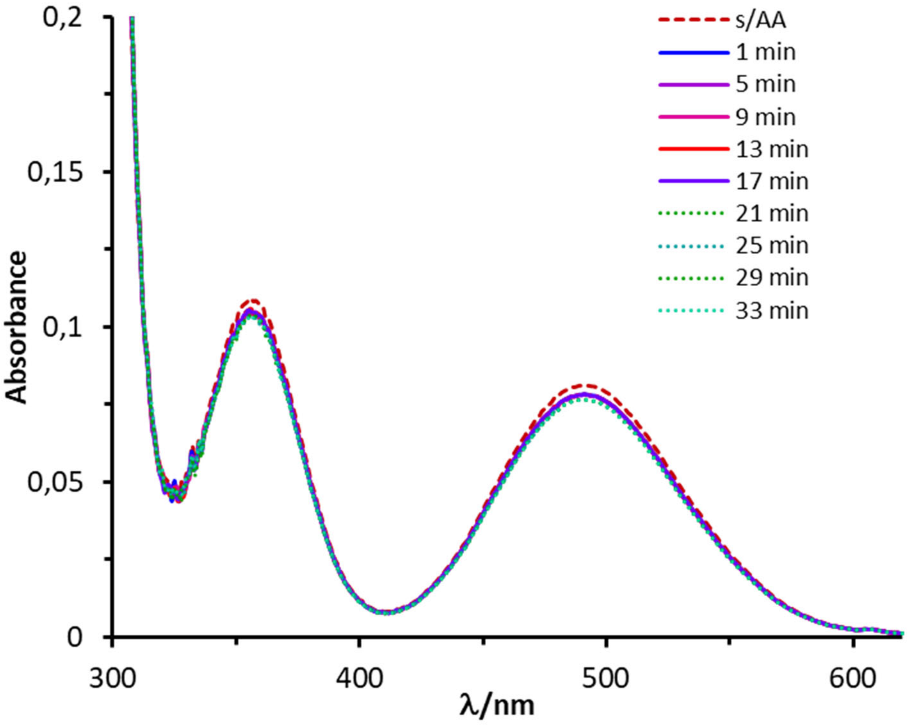

Figure S17. Red dashed line: absorption spectrum of a $1.25 \cdot 10^{-3}$ M solution of the $[Co(CB\text{-}TE2AM)]^{3+}$ complex. Solid lines: absorption spectra of the complex in the presence of ascorbic acid ($1.33 \cdot 10^{-3}$ M). Dashed lines: absorption spectra of the complex in the presence of ascorbic acid ($1.96 \cdot 10^{-3}$ M).

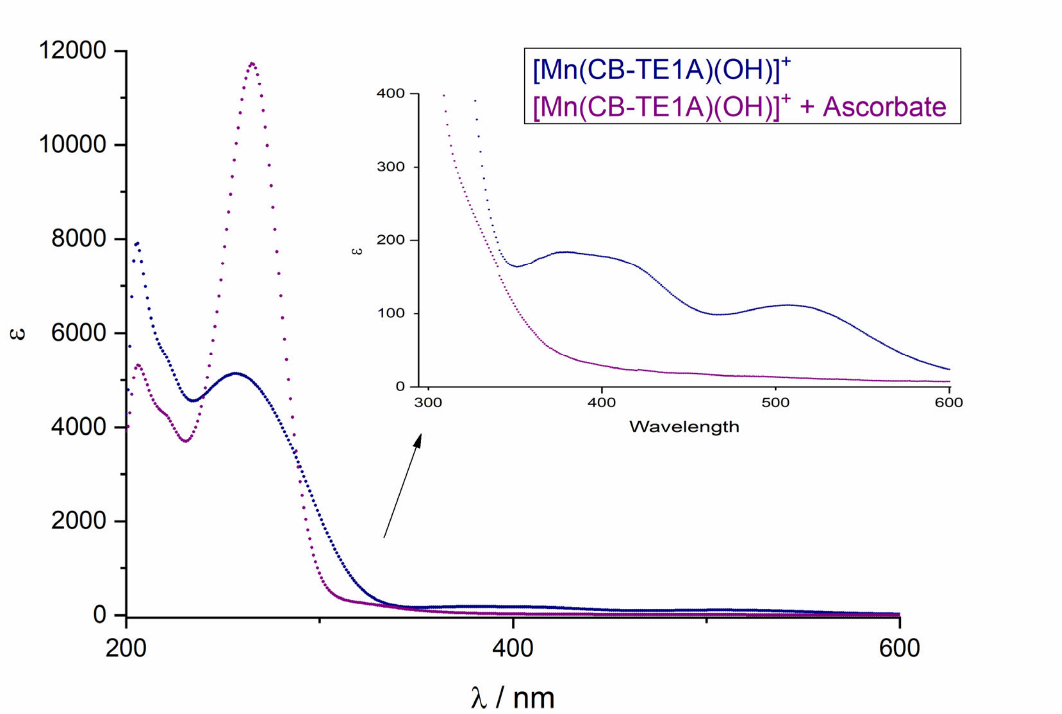

Figure S18. Blue line: Absorption spectrum of a $1.15 \cdot 10^{-4}$ M solution of the $[Mn(CB\text{-}TE1A)(OH)]^{+}$ complex recorded at 298 K, pH 7.4. Purple line: Absorption spectrum of a $9.31 \cdot 10^{-5}$ M solution of the $[Mn(CB\text{-}TE1A)(OH)]^{+}$ complex in the presence of ascorbate recorded at 298 K, pH 7.4.

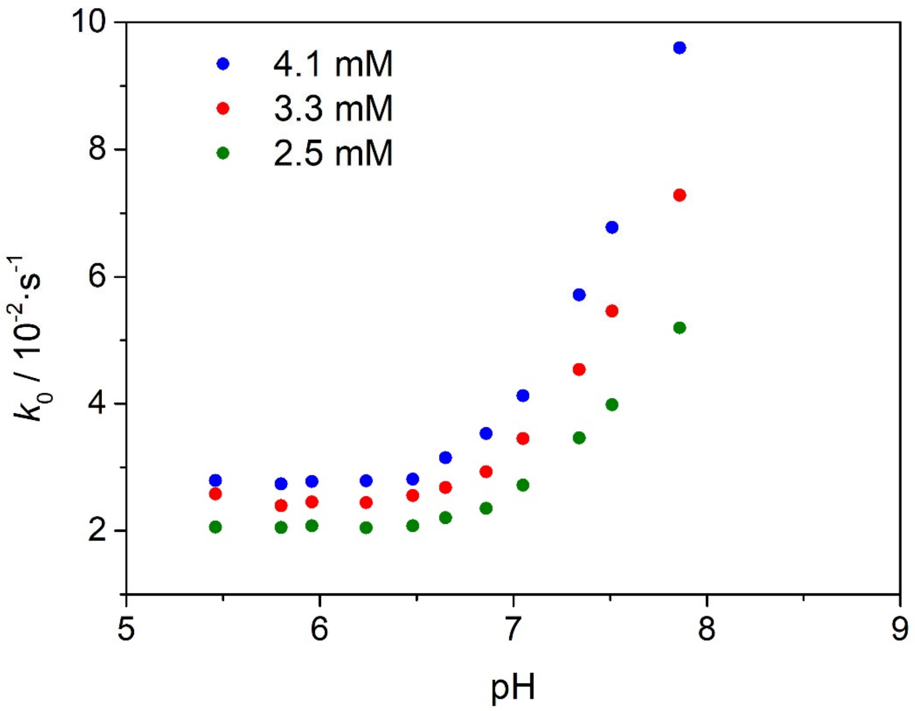

Figure S19. Pseudo-first-order rate constants for the reaction of $[Mn(CB\text{-}TE1A)(OH)]^+$ with ascorbate in phosphate buffer (0.1 M; I = 0.12 M NaCl) as a function of pH. The inset indicates the concentrations of ascorbate.

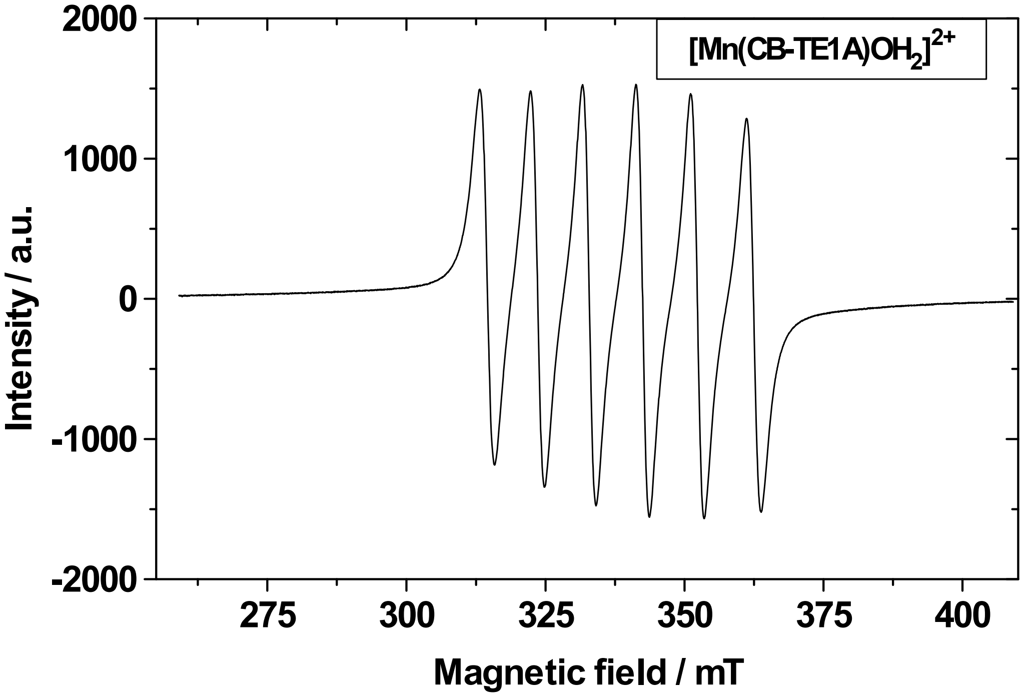

Figure S20. EPR spectrum (X-band) recorded for the $[Mn(CB\text{-}TE1A)(OH_2)]^{2+}$ complex at 298 K.

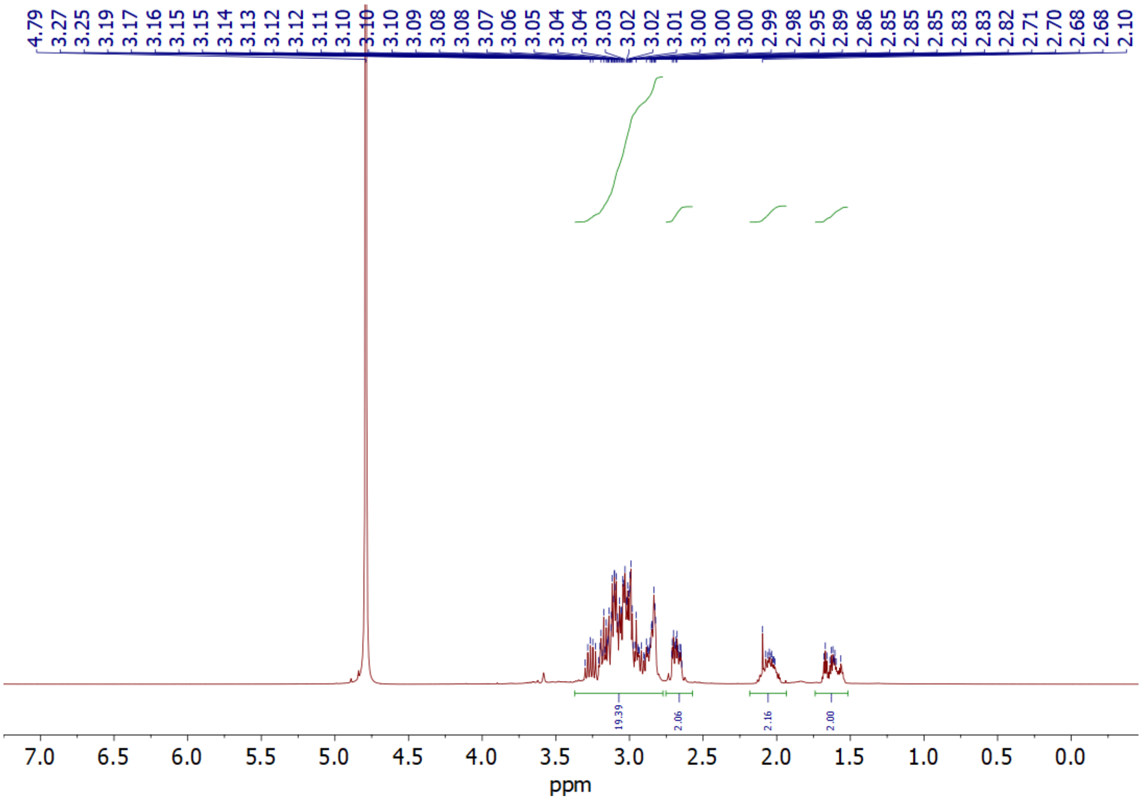

Figure S21. ^{1}H NMR spectrum of CB-TE1AM (400 MHz, D_2O, pH 10.73, 298 K).

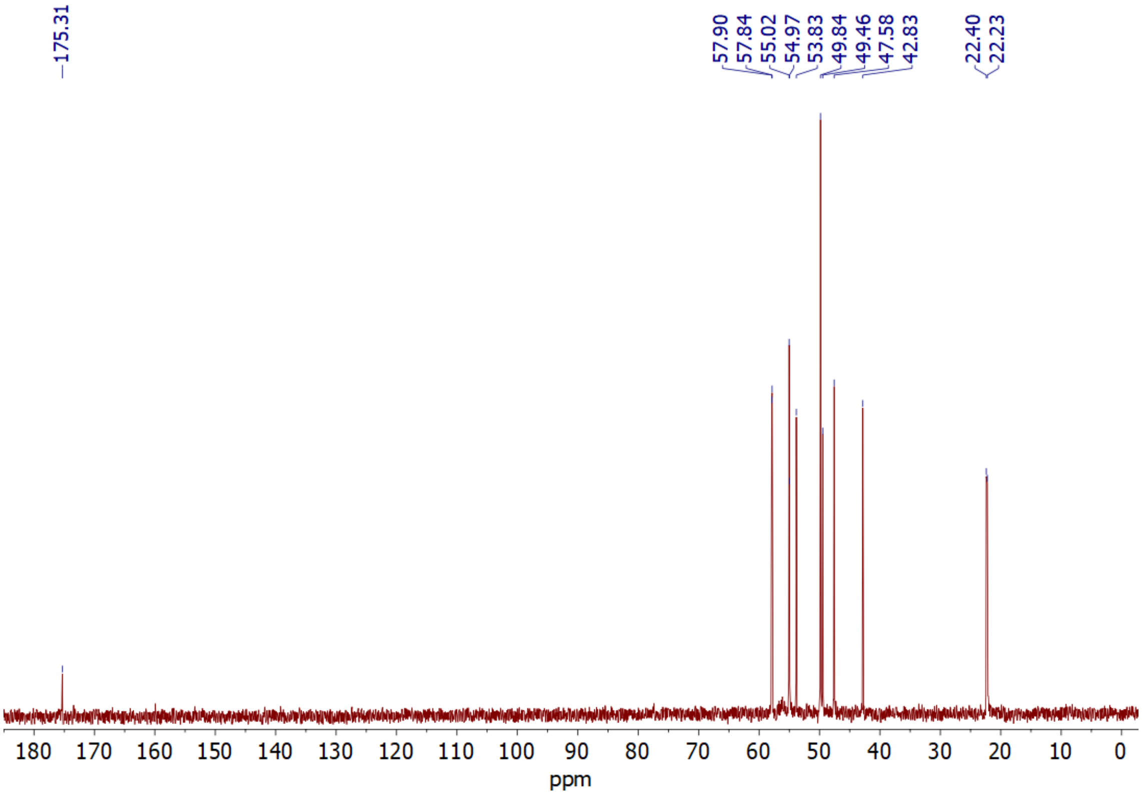

Figure S22. ^{13}C NMR spectrum of CB-TE1AM (101 MHz, D_2O, pH 10.73, 298 K).

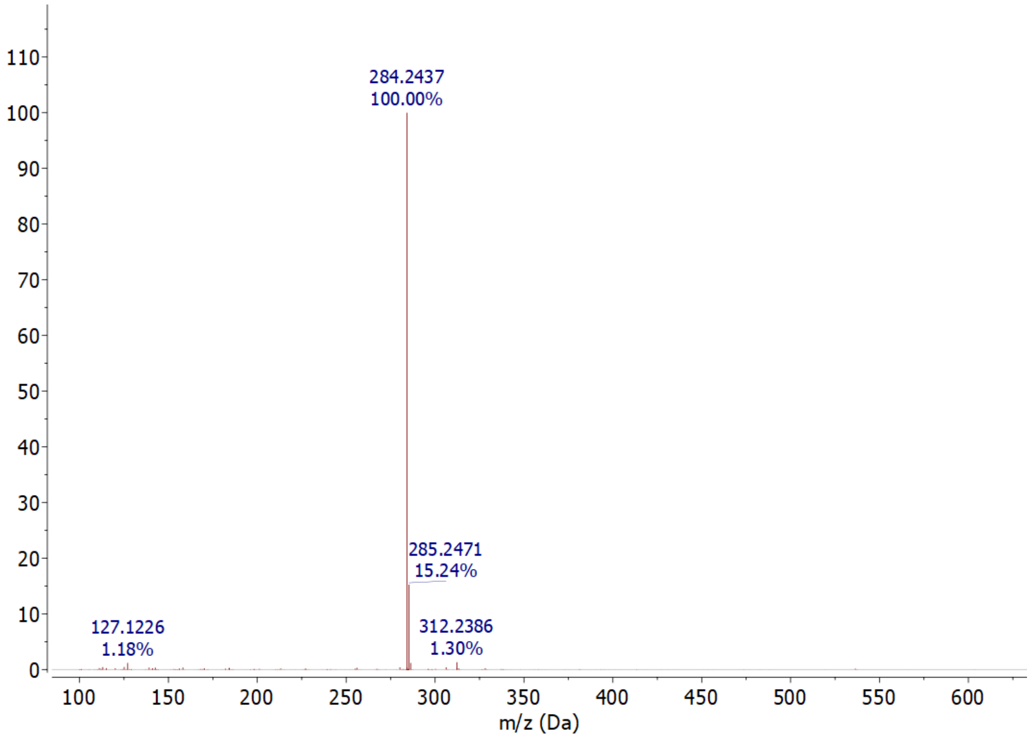

Figure S23. Experimental high resolution mass spectrum (ESI^+) of CB-TE1AM.

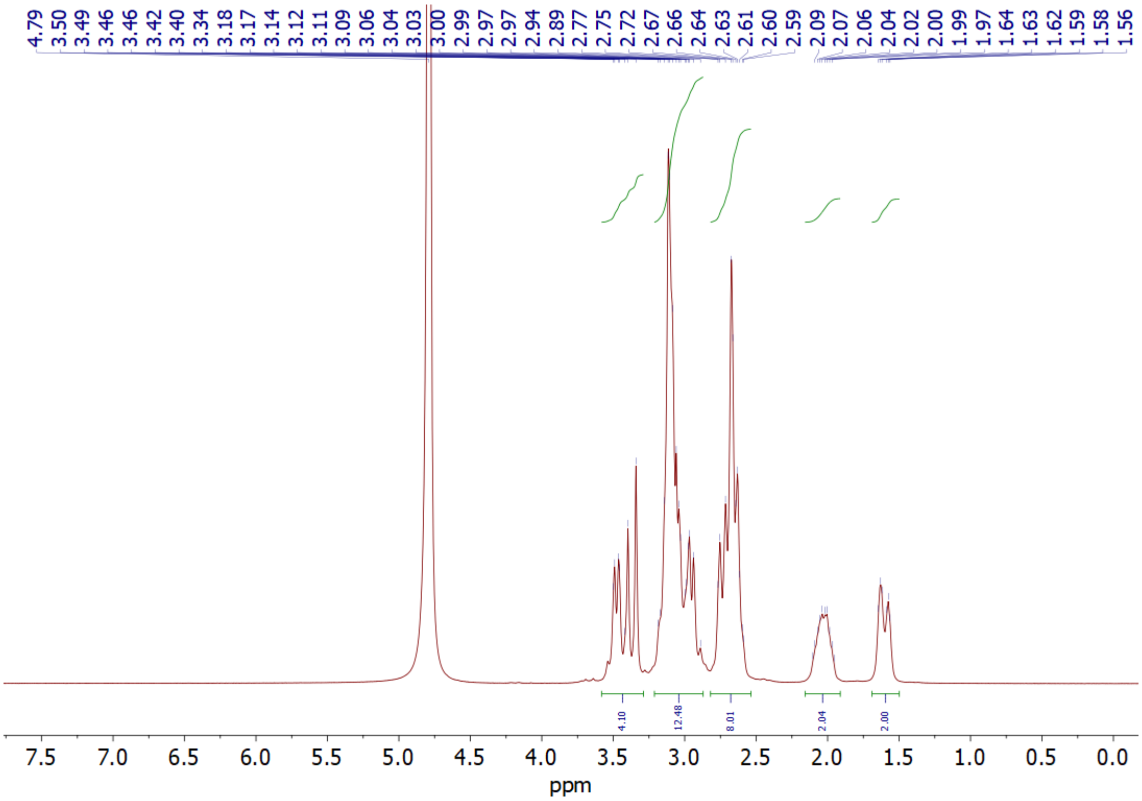

Figure S24. ^{1}H NMR spectrum of CB-TE2AM (300 MHz, D_2O, pH 10.13, 298 K).

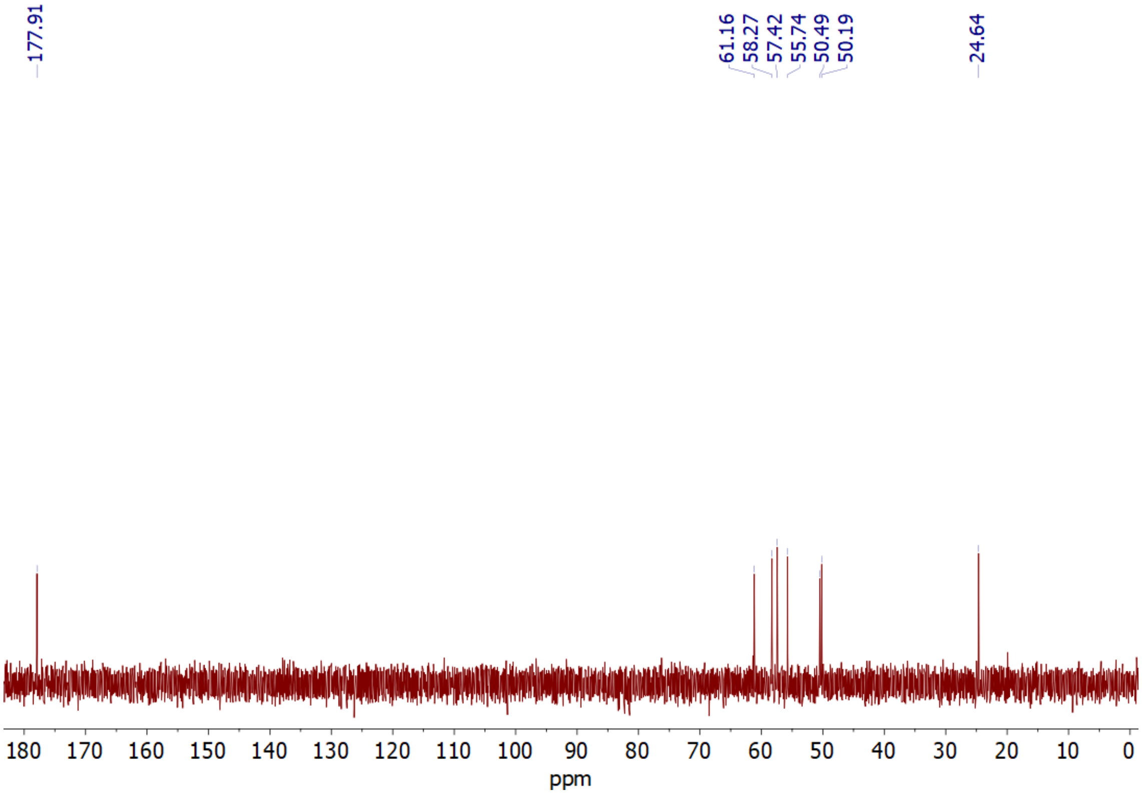

Figure S25. ^{13}C NMR spectrum of CB-TE2AM (75 MHz, D_2O, pH 10.13, 298 K).

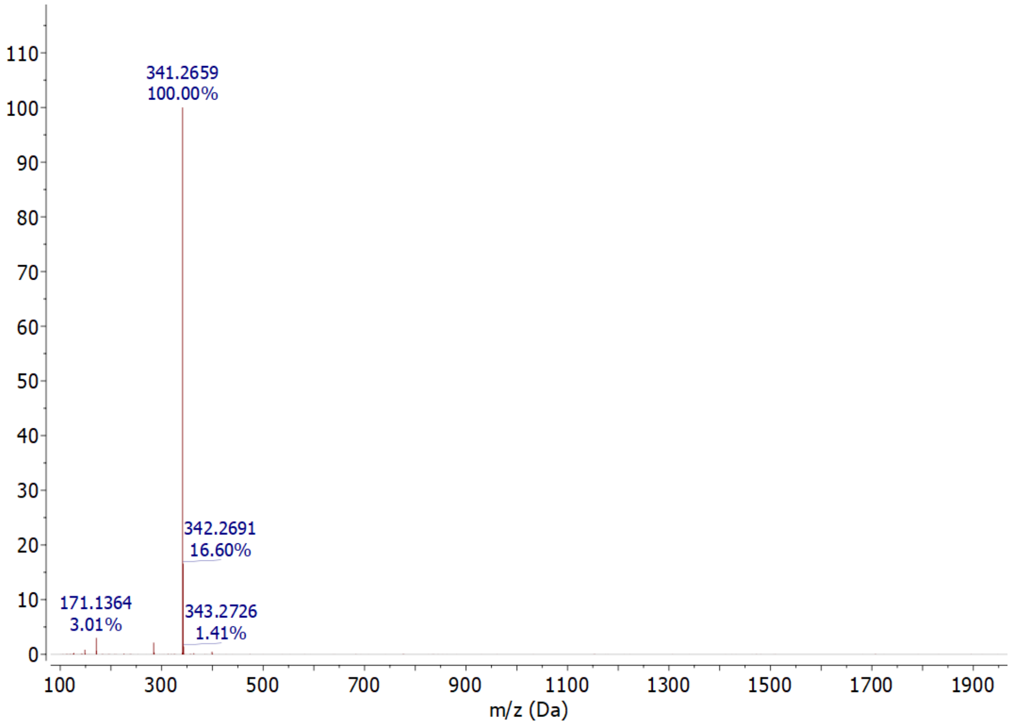

Figure S26. Experimental high resolution mass spectrum (ESI^+) of CB-TE2AM.

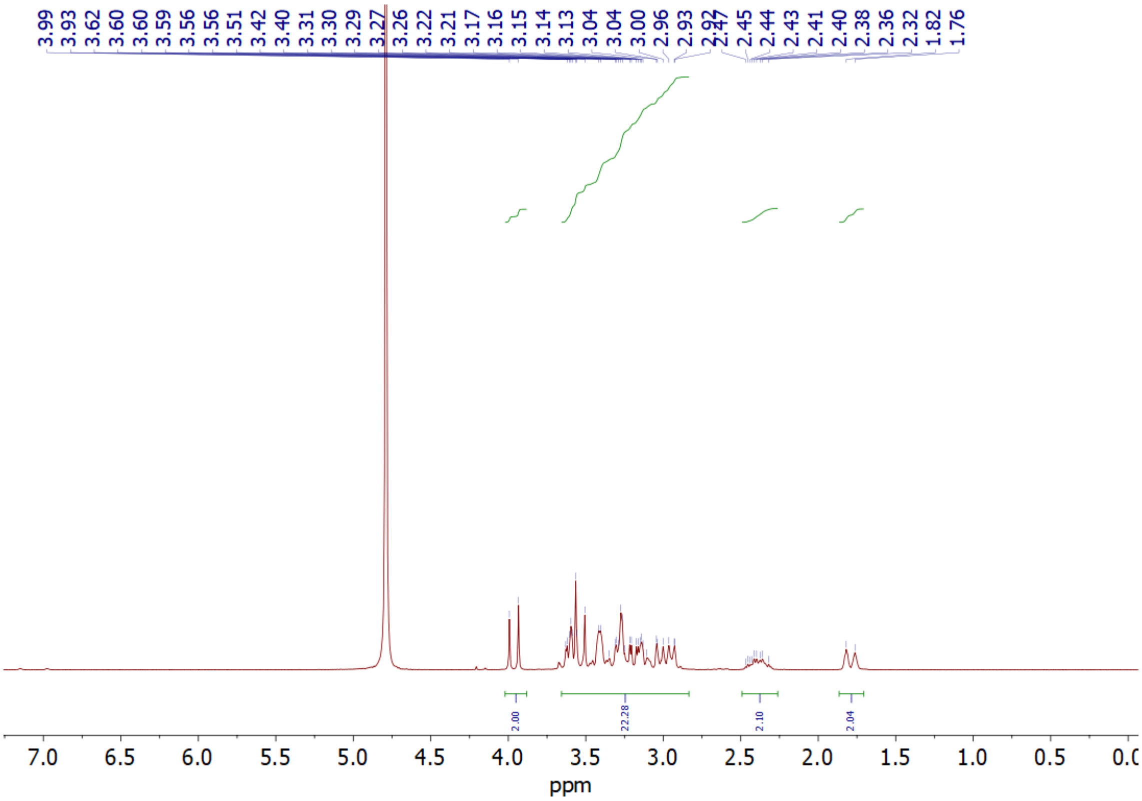

Figure S27. ^{1}H NMR spectrum of H_2CB-TE2A (300 MHz, D_2O, pH 0.61, 298 K).

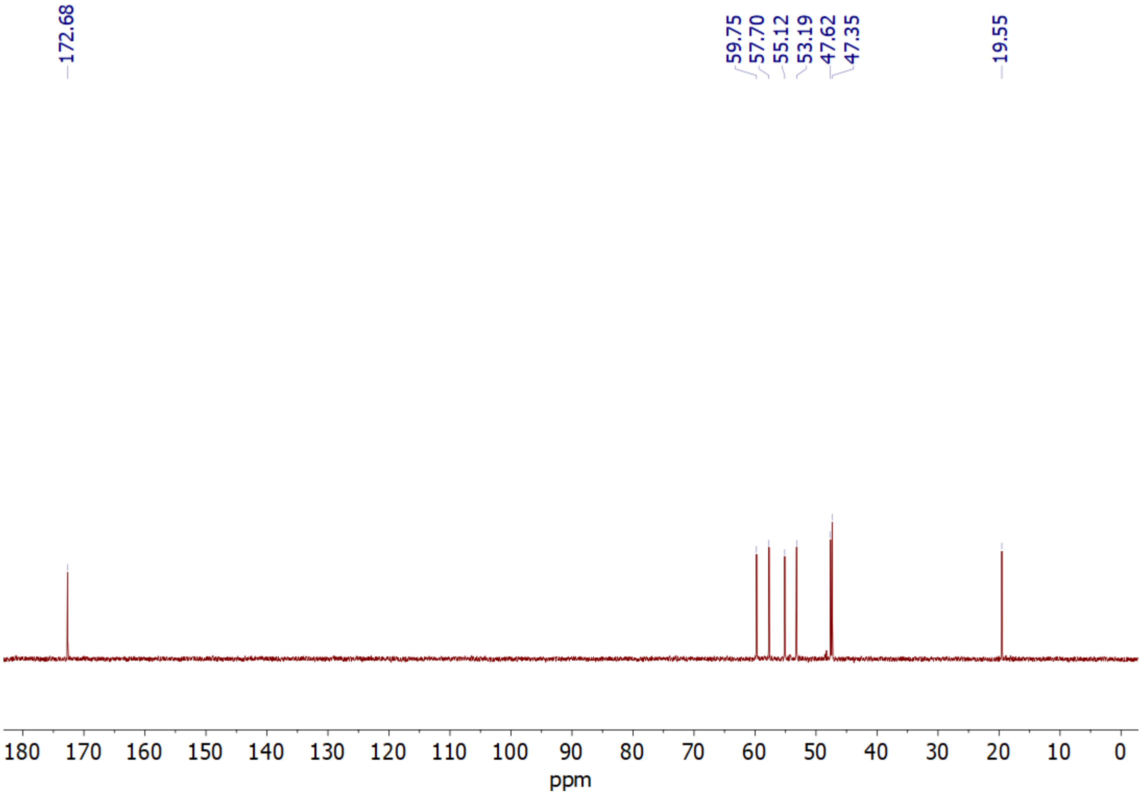

Figure S28. ^{13}C NMR spectrum of H_2CB-TE2A (75 MHz, D_2O, pH 0.61, 298 K).

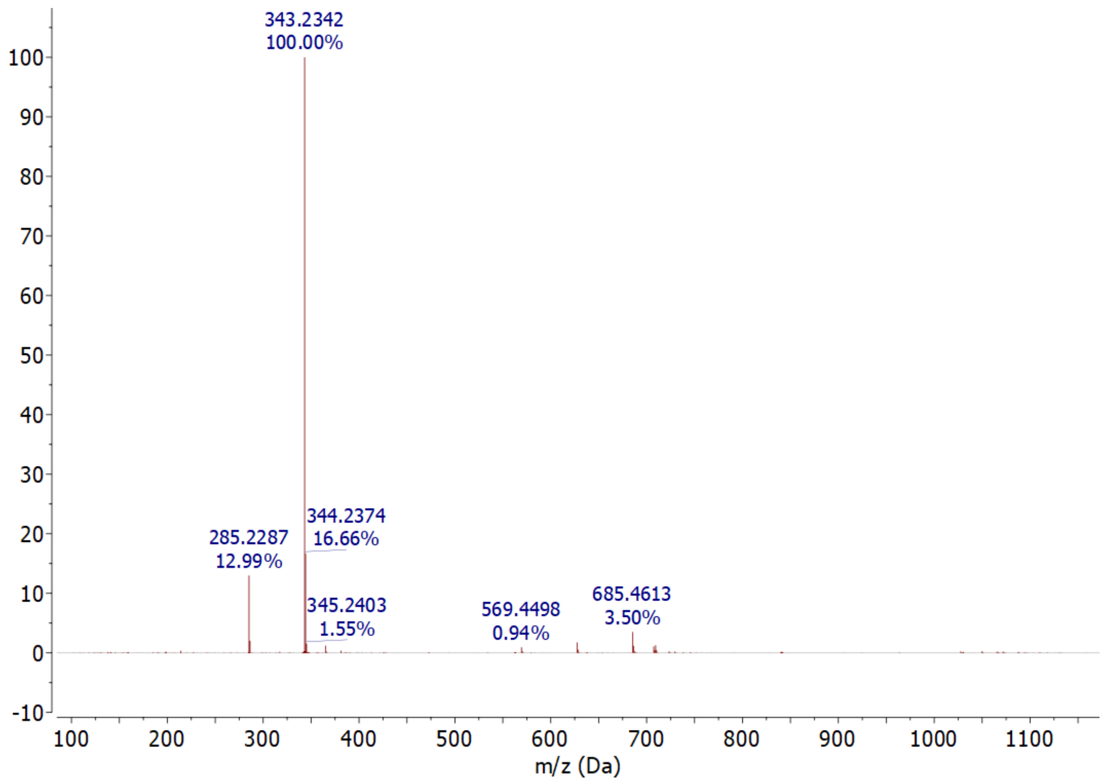

Figure S29. Experimental high resolution mass spectrum (ESI^+) of H_2CB-TE2A.

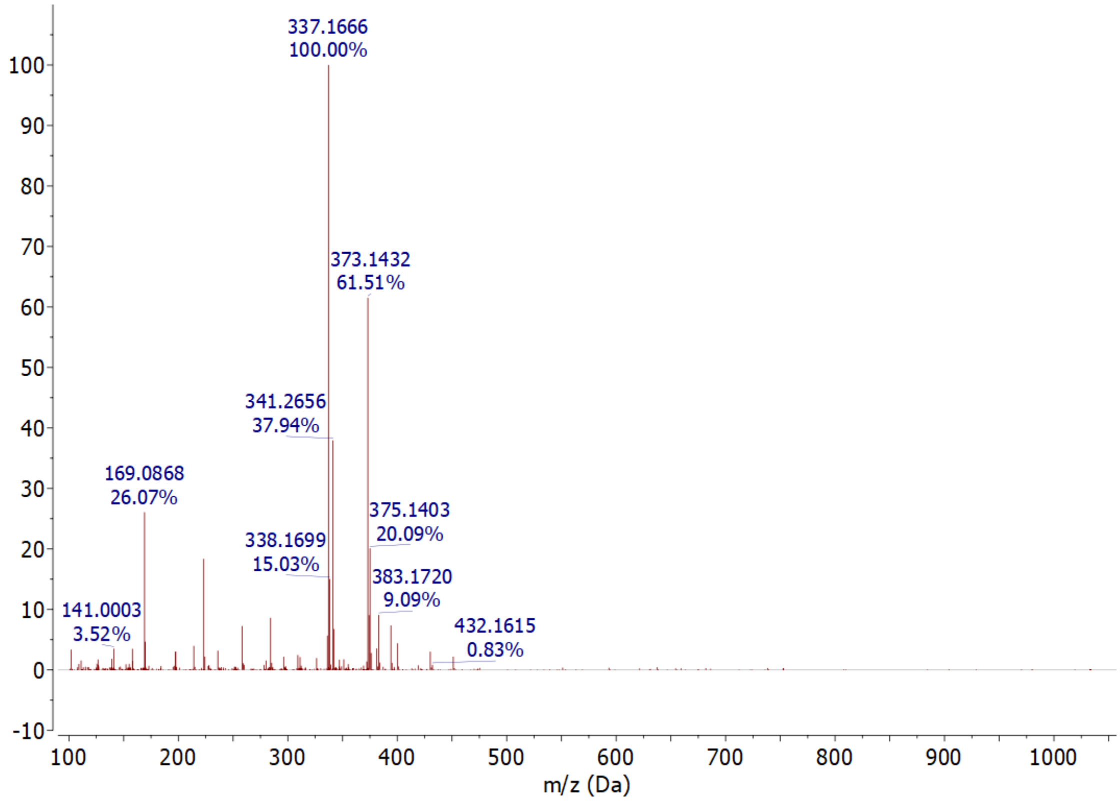

Figure S30. Experimental high resolution mass spectrum (ESI$^+$) of $[Mn(CB\text{-}TE1AM)(OH)]Cl_2$.

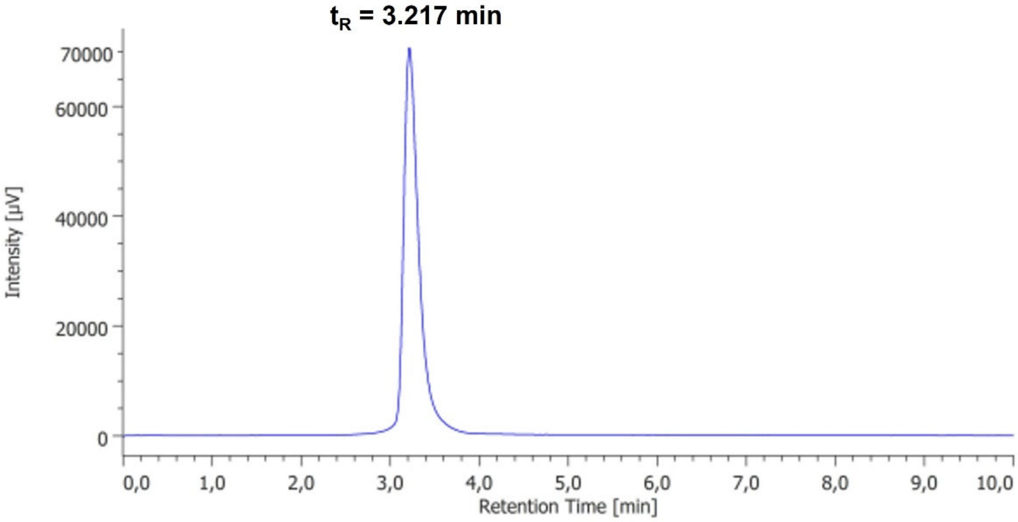

Figure S31. HPLC chromatogram of $[Mn(CB\text{-}TE1AM)(OH)]Cl_2$ at 400 nm.

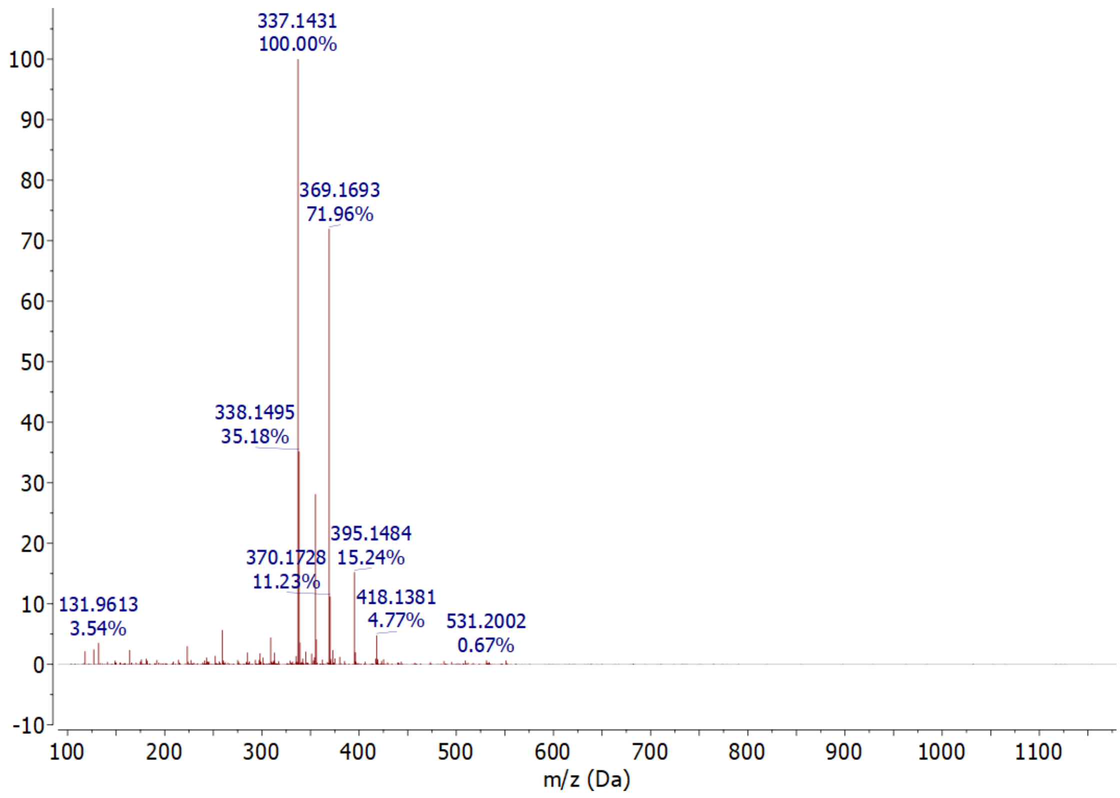

Figure S32. Experimental high resolution mass spectrum (ESI^+) of [Mn(CB-TE1A)(OH)]Cl.

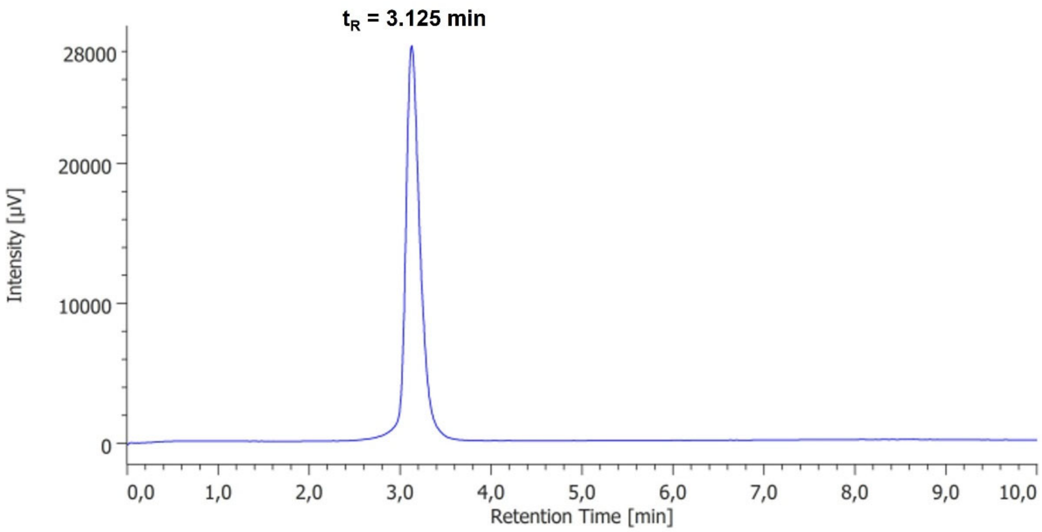

Figure S33. HPLC chromatogram of [Mn(CB-TE1A)(OH)]Cl at 400 nm.

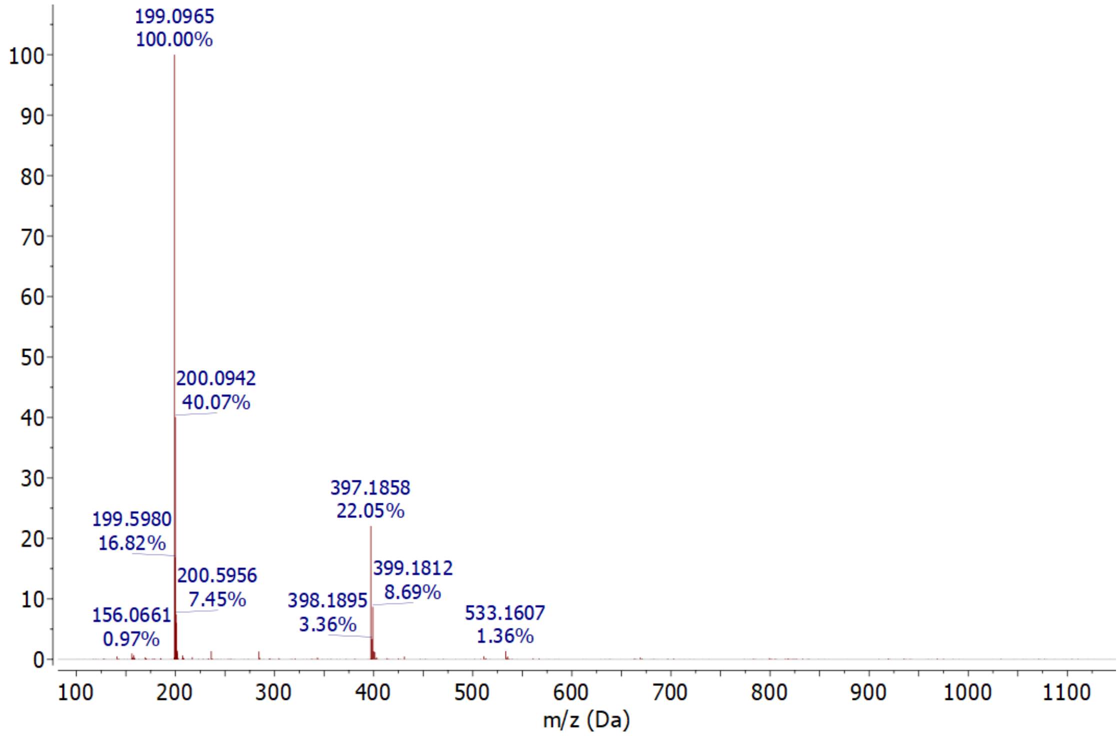

Figure S34. Experimental high resolution mass spectrum (ESI$^+$) of [Ni(CB-TE2AM)]Cl_2

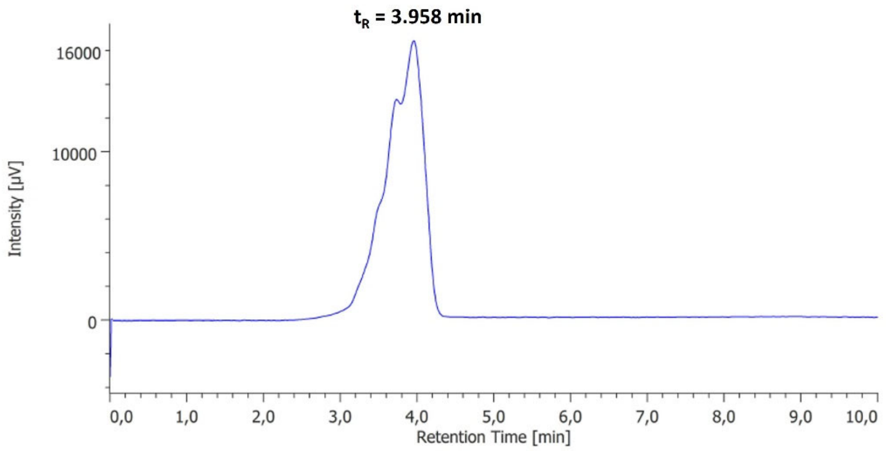

Figure S35. HPLC chromatogram of [Ni(CB-TE2AM)]Cl_2 at 400 nm.

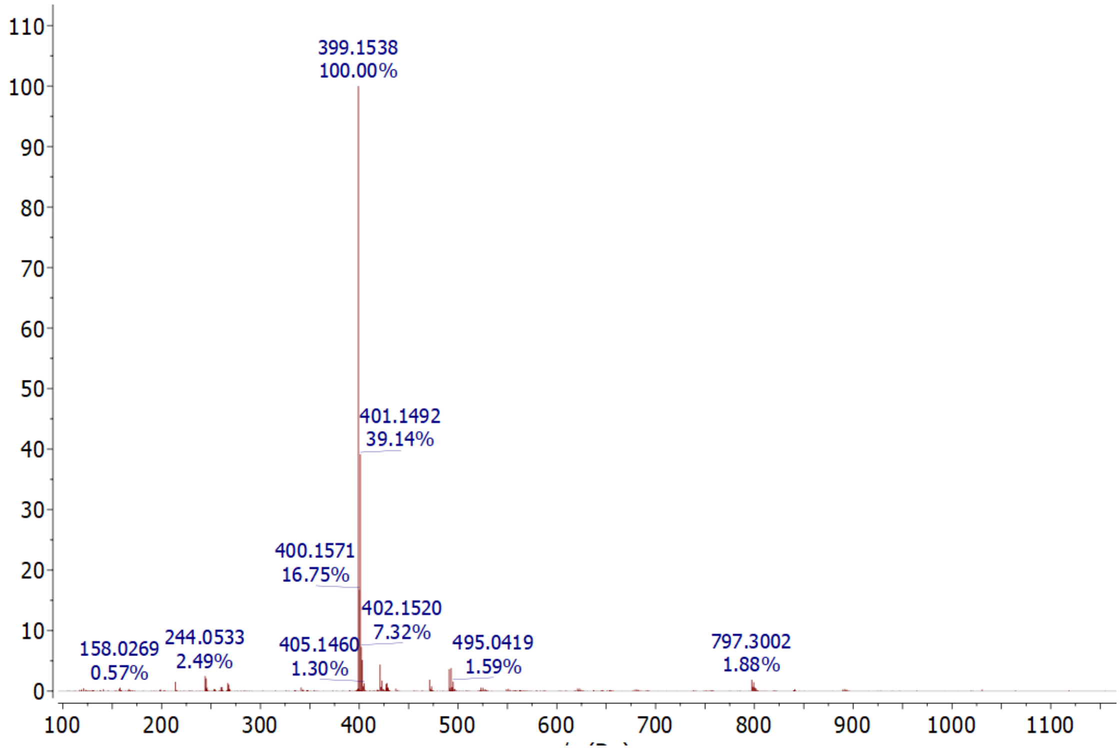

Figure S36. Experimental high resolution mass spectrum (ESI$^+$) of [Ni(CB-TE2A)].

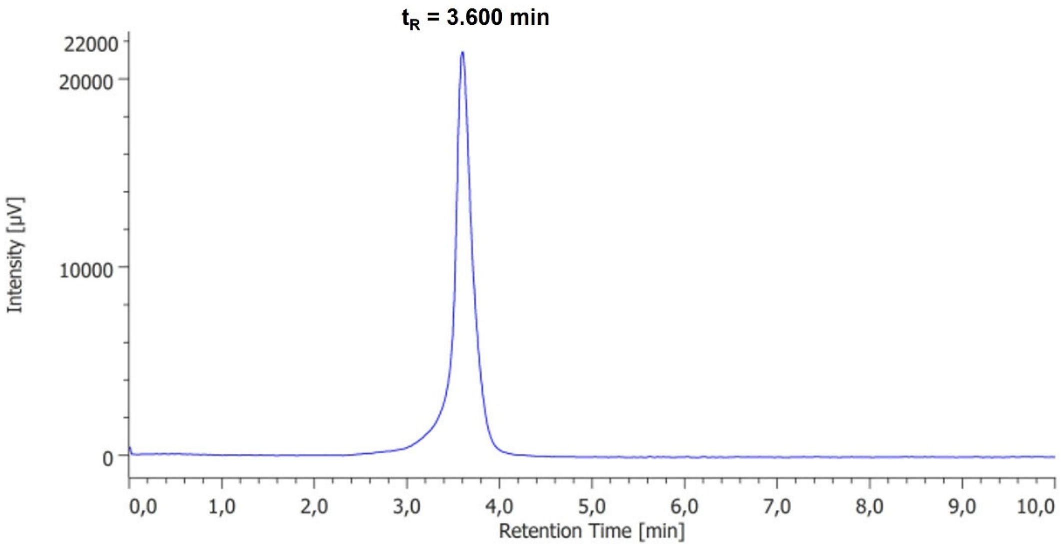

Figure S37. HPLC chromatogram of [Ni(CB-TE2A)] at 517 nm.

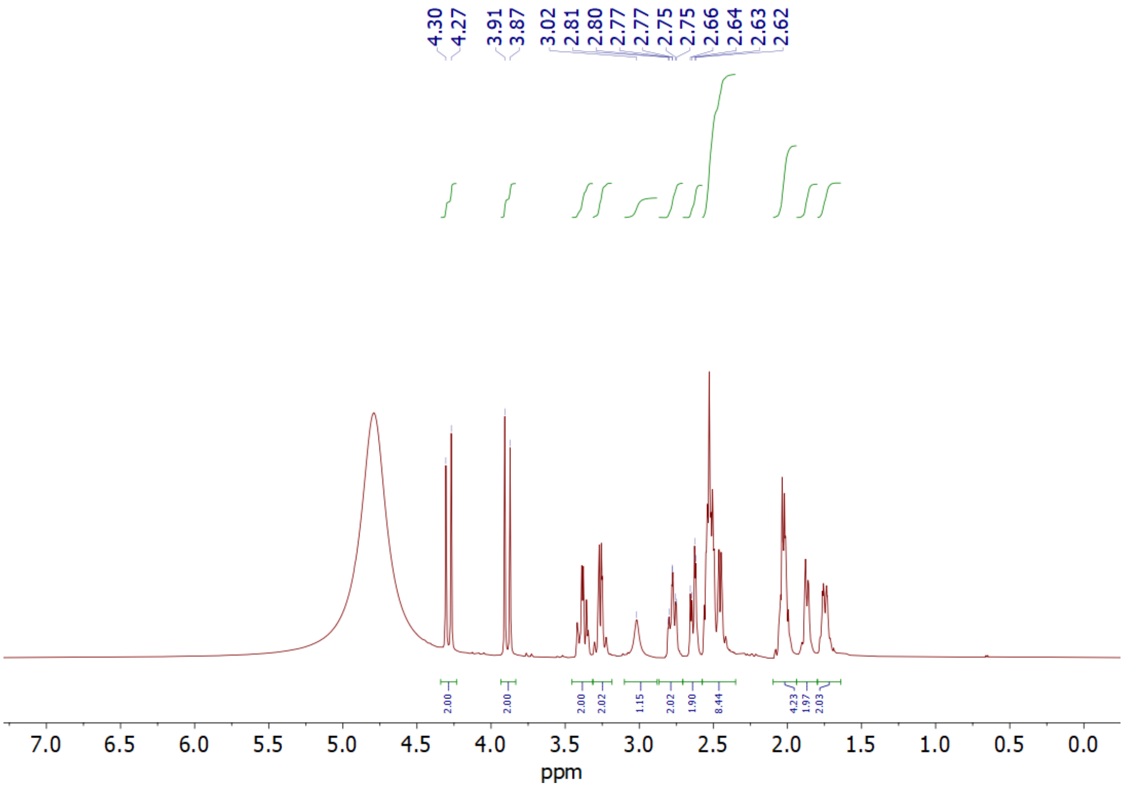

Figure S38. ^{1}H NMR spectrum of [Co(CB-TE2AM)]Cl_3 (500 MHz, D_2O, pH 3.76, 298 K).

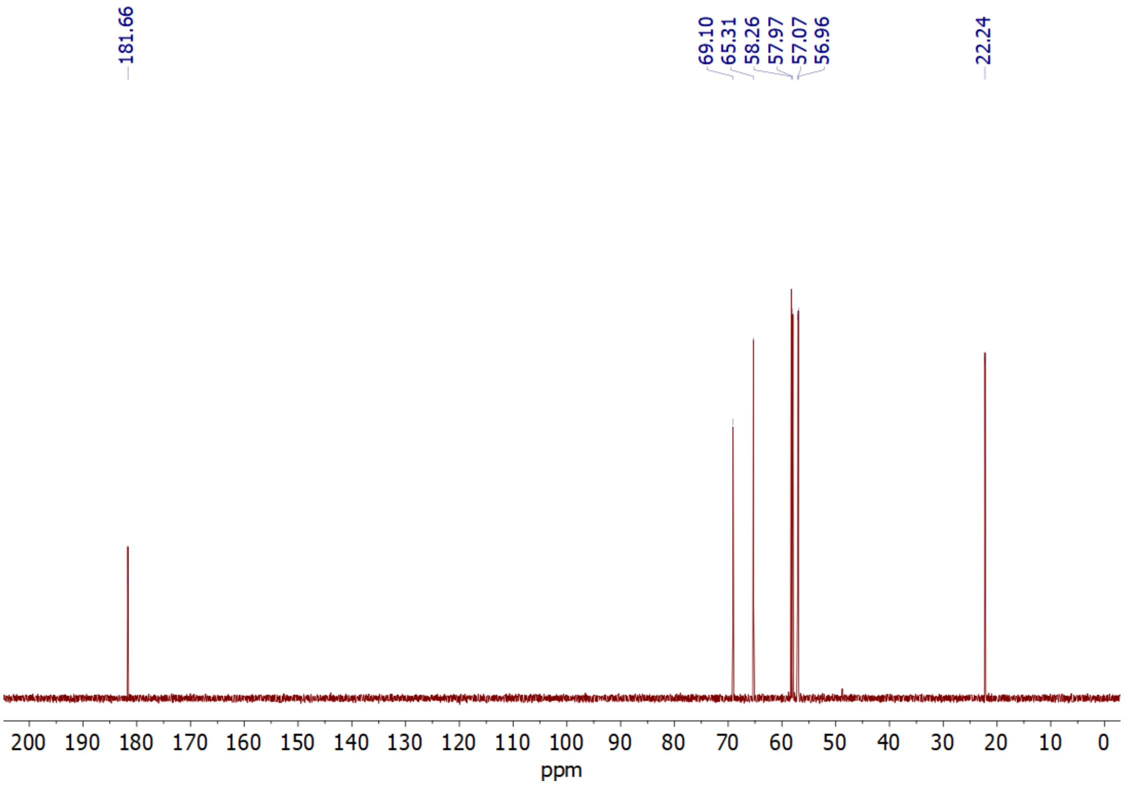

Figure S39. ^{13}C NMR spectrum of [Co(CB-TE2AM)]Cl_3 (101 MHz, D_2O, pH 3.76, 298 K).

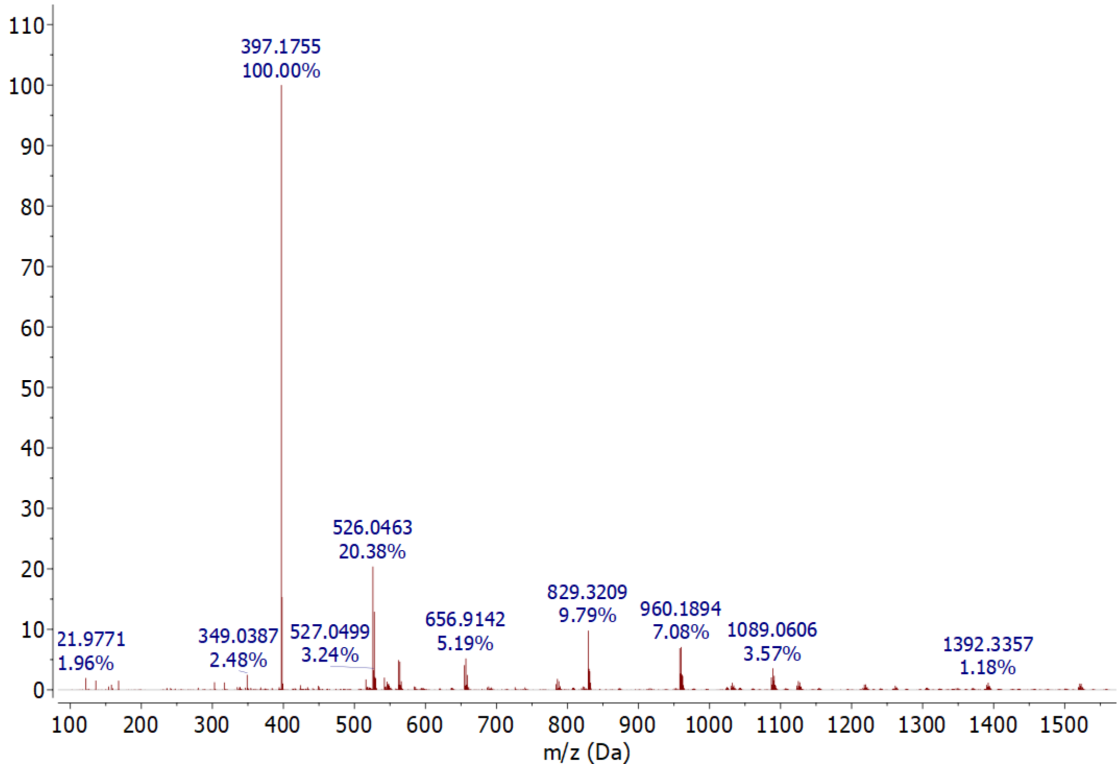

Figure S40. Experimental high resolution mass spectrum (ESI$^+$) of [Co(CB-TE2AM)]Cl$_3$.

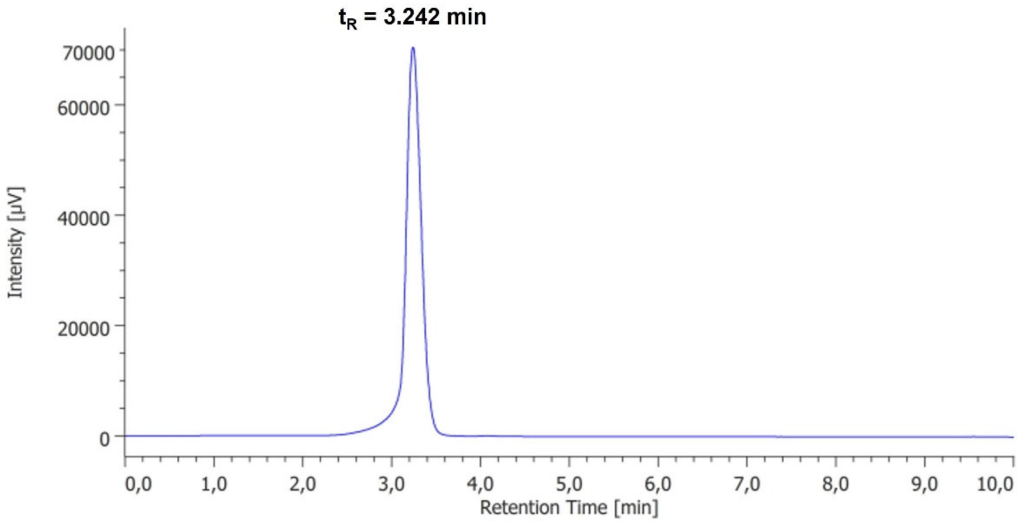

Figure S41. HPLC chromatogram of [Co(CB-TE2AM)]Cl$_3$ at 352 nm.

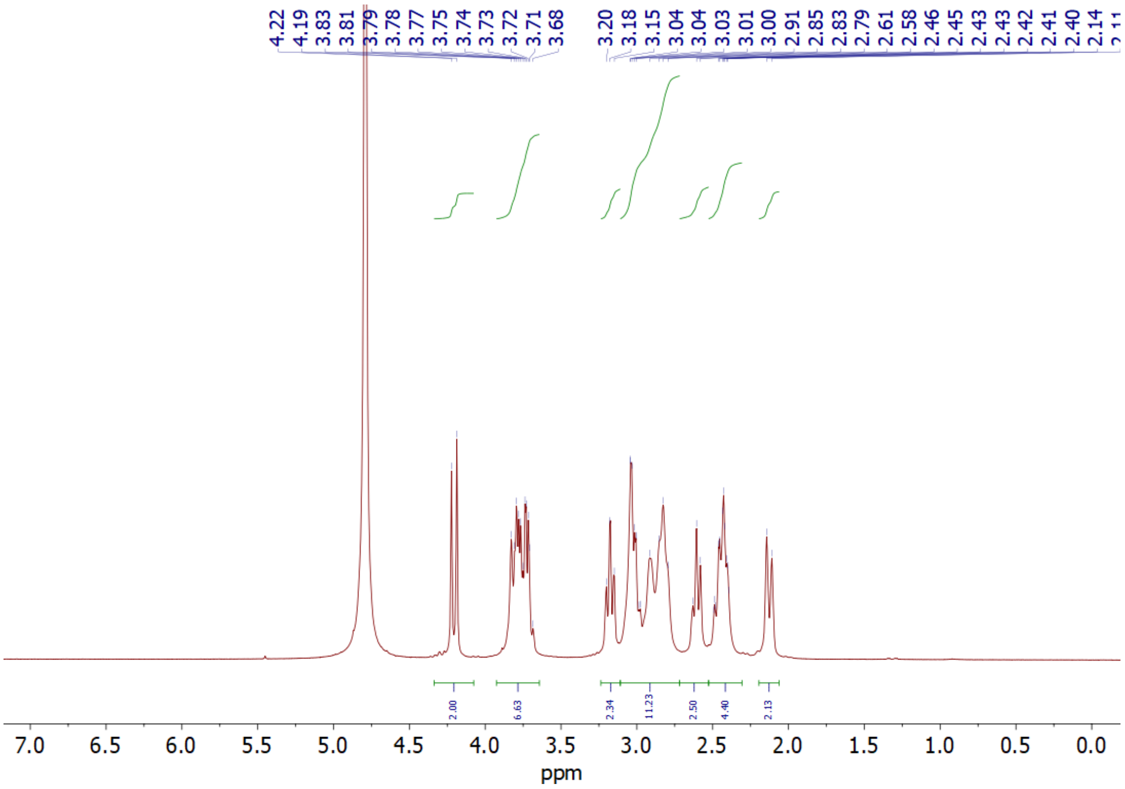

Figure S42. ^{1}H NMR spectrum of [Co(CB-TE2A)]Cl (500 MHz, D_2O, pH 7.40, 298 K).

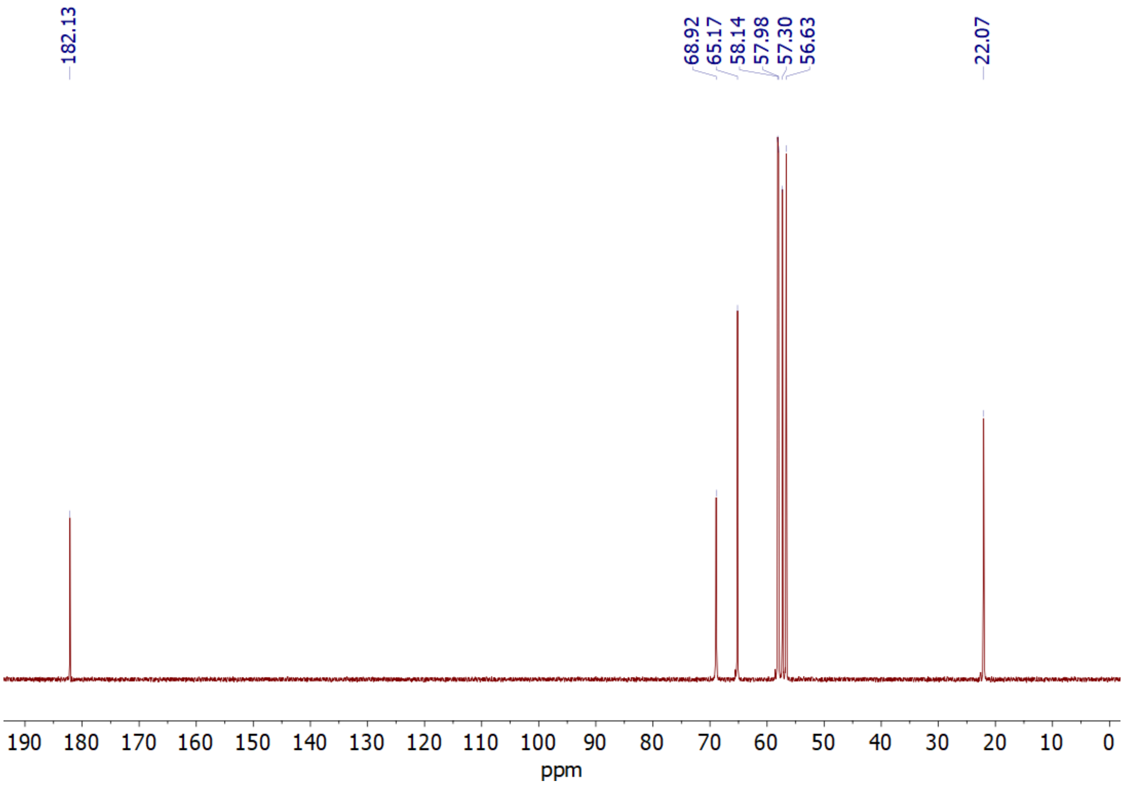

Figure S43. ^{13}C NMR spectrum of [Co(CB-TE2A)]Cl (101 MHz, D_2O, pH 7.40, 298 K).

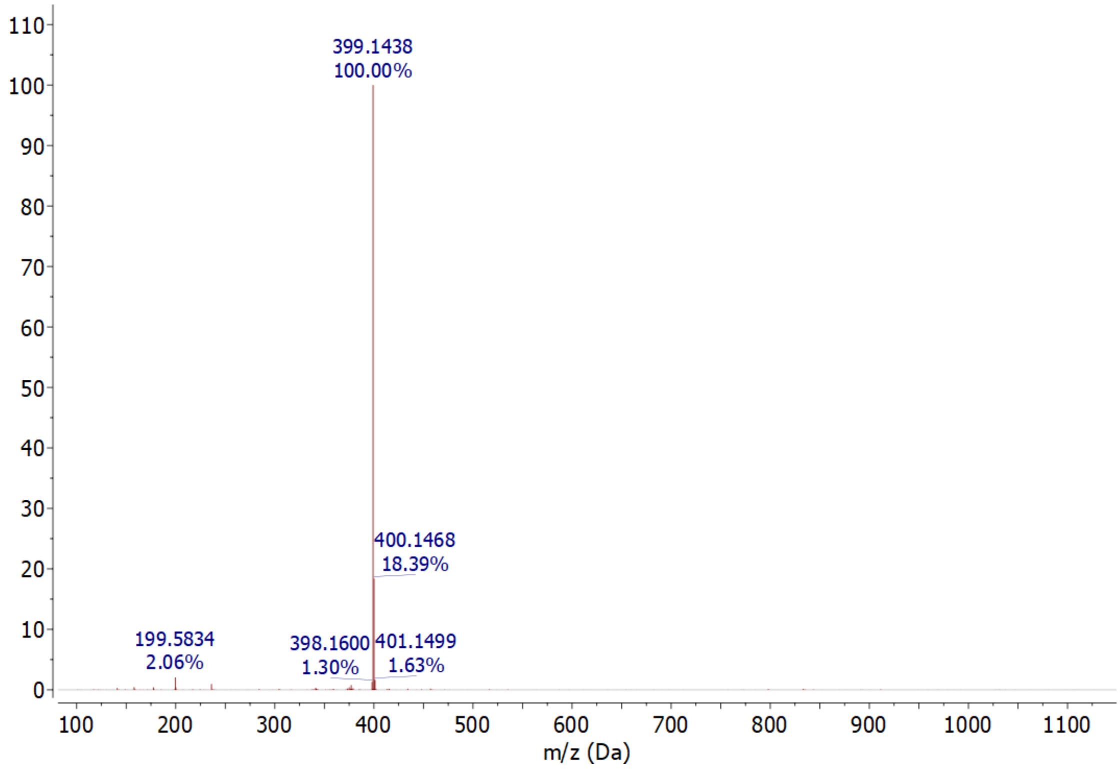

Figure S44. Experimental high resolution mass spectrum (ESI^+) of [Co(CB-TE2A)]Cl.

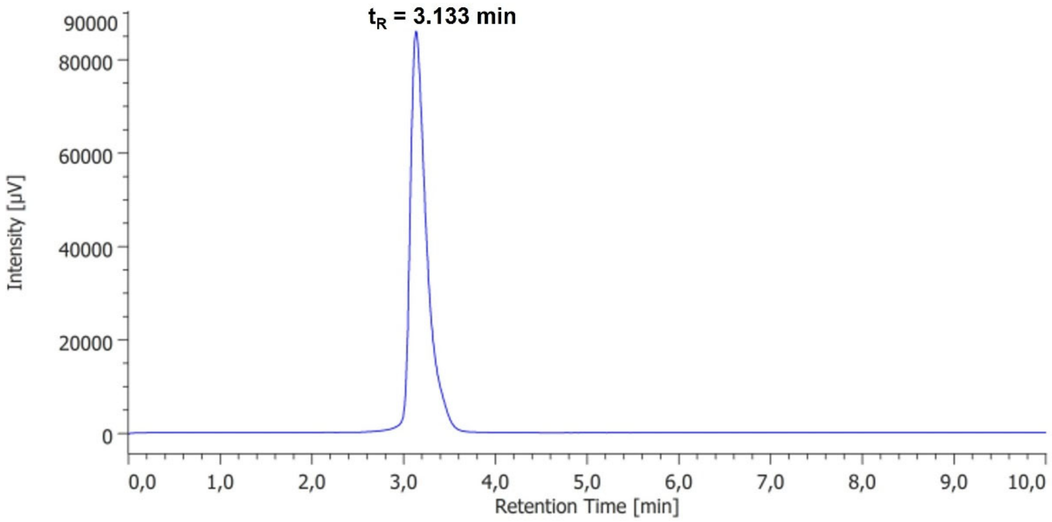

Figure S45. HPLC chromatogram of [Co(CB-TE2A)]Cl at 355 nm.

Table S1. Assignment of the ^1H and ^{13}C spectra of the $[Co(CB\text{-}TE2AM)]^{3+}$ complex.

^{13}C	δ_C (ppm)	^1H	δ_H (ppm)
1	181.66	H_{1ax}	--
		H_{1eq}	--
2	69.10	H_{2ax}	3.89
		H_{2eq}	4.29
3	57.07	H_{3ax}	2.75
		H_{3eq}	2.51
4	22.24	H_{4ax}	1.74
		H_{4eq}	2.04
5	57.97	H_{5ax}	2.03
		H_{5eq}	1.87
6	65.31	H_{6ax}	2.55
		H_{6eq}	2.64
7	56.96	H_{7ax}	3.38
		H_{7eq}	2.49
8	58.26	H_{8ax}	2.45
		H_{8eq}	3.26

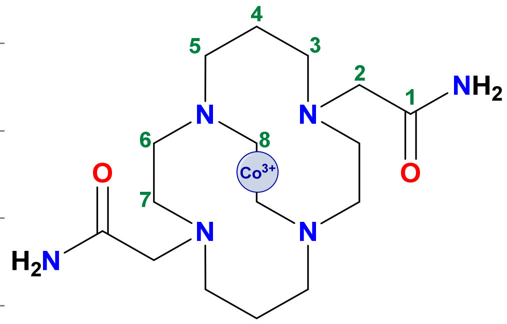

Table S2. Assignment of the ^1H and ^{13}C spectra of the $[Co(CB\text{-}TE2A)]^{+}$ complex.

^{13}C	δ_C (ppm)	^1H	δ_H (ppm)
1	182.13	H_{1ax}	--
		H_{1eq}	--
2	68.92	H_{2ax}	3.73
		H_{2eq}	4.12
3	57.98	H_{3ax}	2.52
		H_{3eq}	2.34
4	22.07	H_{4ax}	2.38
		H_{4eq}	2.04
5	56.63	H_{5ax}	3.08
		H_{5eq}	2.76
6	65.17	H_{6ax}	2.94
		H_{6eq}	2.94
7	57.30	H_{7ax}	3.68
		H_{7eq}	2.72
8	58.14	H_{8ax}	2.83
		H_{8eq}	3.64

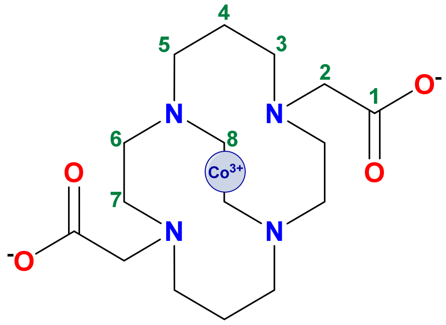

Table S3. HPLC analytical method.

Time (min)	Composition (%)	
	H_2O	CH_3CN
1	100	0
4	100	0
8	5	95
10	5	95

Table S4. Crystal data and structure refinement details of $[Ni(CB\text{-}TE2AM)]Cl_2{\cdot}2H_2O$.

**Parameter**	**Value**
Formula	$C_{16}H_{32}Cl_2N_6NiO_4$
Molecular weight, MW	502.08
Crystal system	Monoclinic
Space group	$P2_1/c$
a	9.0398(10) Å
b	14.7690(16) Å
c	17.0424(18) Å
V	2227.8(4) Å^3
F(000)	1056
Z	4
D_{calc}	1.497 g cm^{-3}
μ	1.145
θ range	2.30º-28.33º
R_{int}	0.0387
Measured reflections	42599[a]
Goodness of fit, GOF on F^2	1.033
R1	0.0277
wR2 (all data)	0.0682
Larg. Diff. peak and hole (eÅ^{-3})	0.53 and -0.28

[a] Of which 5544 were independent and 4695 were unique, with I >2σ (I).

Table S5. Crystal data and structure refinement details of $[Mn(CB\text{-}TE1AM)(OH)](PF_6)_2$.

**Parameter**	**Value**
Formula	$C_{14}H_{32}F_{12}MnN_5O_3P_2$
Molecular weight, MW	663.32
Crystal system	Monoclinic
Space group	$P2_1/c$
a	10.2279(6) Å
b	19.1955(10) Å
c	12.7128(6) Å
V	2444.7(2) Å^3
F(000)	1352
Z	4
D_{calc}	1.802 g cm^{-3}
μ	0.791
θ range	2.29°-28.34°
R_{int}	0.0357
Measured reflections	86402[a]
Goodness of fit, GOF on F^2	1.088
R1	0.0422
wR2 (all data)	0.0994
Larg. Diff. peak and hole (eÅ^{-3})	0.92 and -0.54

[a] Of which 6090 were independent and 5519 were unique, with I >2σ (I).

Table S6. Parameters of the three-pool system BM fit of $[Ni(CB\text{-}TE2AM)]^{2+}$ at 298 K.[a]

Parameter	Start value	Lower bound	Upper bound	Fitted value
$\delta\omega_A$[b] / ppm	0	-1	1	0.014 ± 0.002
$R_{2,A}$ / s^{-1}	2	0.2	40000	9.47 ± 0.09
$\delta\omega_B$ / ppm	70	65	75	71.4 ± 0.1
χ_B[c]	0.000126	0.000025	0.000630	0.00051 ± 0.00003
$k_{ex,B}$ / s^{-1}	2000	200	20000	1460 ± 130
$R_{2,B}$ / s^{-1}	50	0	5000	4002 ± 260
$\delta\omega_C$ / ppm	4	-1	9	2.0 ± 0.2
χ_C	0.000126	0.000025	0.000630	0.00063 ± 0.00006
$k_{ex,C}$ / s^{-1}	1000	100	10000	1384 ± 151
$R_{2,C}$ / s^{-1}	50	0	5000	2006 ± 161

[a] Pool A – bulk water, pools B and C – *trans* and *cis* amide protons, respectively. [b] Chemical shift of the exchanging pool. [c] Fractional concentration of the exchanging pool, representing ratio of concentration of exchanging protons with total proton concentration of bulk water, i.e. χ_B=χ_C=(2×7×10^{-3} M)/(111 M)=0.000126.

Table S7. Parameters of the two-pool system BM fit of $[Ni(CB\text{-}TE2AM)]^{2+}$ at 310 K.[a]

Parameter	Start value	Lower bound	Upper bound	Fitted value
$\delta\omega_A$[b] / ppm	0	-1	1	0.064 ± 0.006
$R_{2,A}$ / s^{-1}	2	0.2	40000	6.76 ± 0.09
$\delta\omega_B$ / ppm	70	65	75	68.4 ± 0.1
χ_B[c]	0.000126	0.000025	0.000630	0.00050 ± 0.00004
$k_{ex,B}$ / s^{-1}	2000	200	20000	2064 ± 295
$R_{2,B}$ / s^{-1}	50	0	5000	3740 ± 348

[a] Pool A – bulk water, pools B – *trans* amide protons. [b] Chemical shift of the exchanging pool. [c] Fractional concentration of the exchanging pool, representing ratio of concentration of exchanging protons with total proton concentration of bulk water, i.e. $\chi_B=\chi_C=(2\times7\times10^{-3}\ M)/(111\ M)=0.000126$.
